# Supplementary material for: Topical phage therapy in a mouse model of Cutibacterium acnes-induced acne-like lesions
Source: Nat Commun. 2023 Feb 22;14:1005. doi: 10.1038/s41467-023-36694-8 (PMC9947178; doi:10.1038/s41467-023-36694-8)
Supplement: Supplementary file 1 — Supplementary Information [file 41467_2023_36694_MOESM1_ESM.pdf]

Supplementary Information

Topical phage therapy in a mouse model of *Cutibacterium acn*es-induced acne-like lesions

Rimon *et al.*

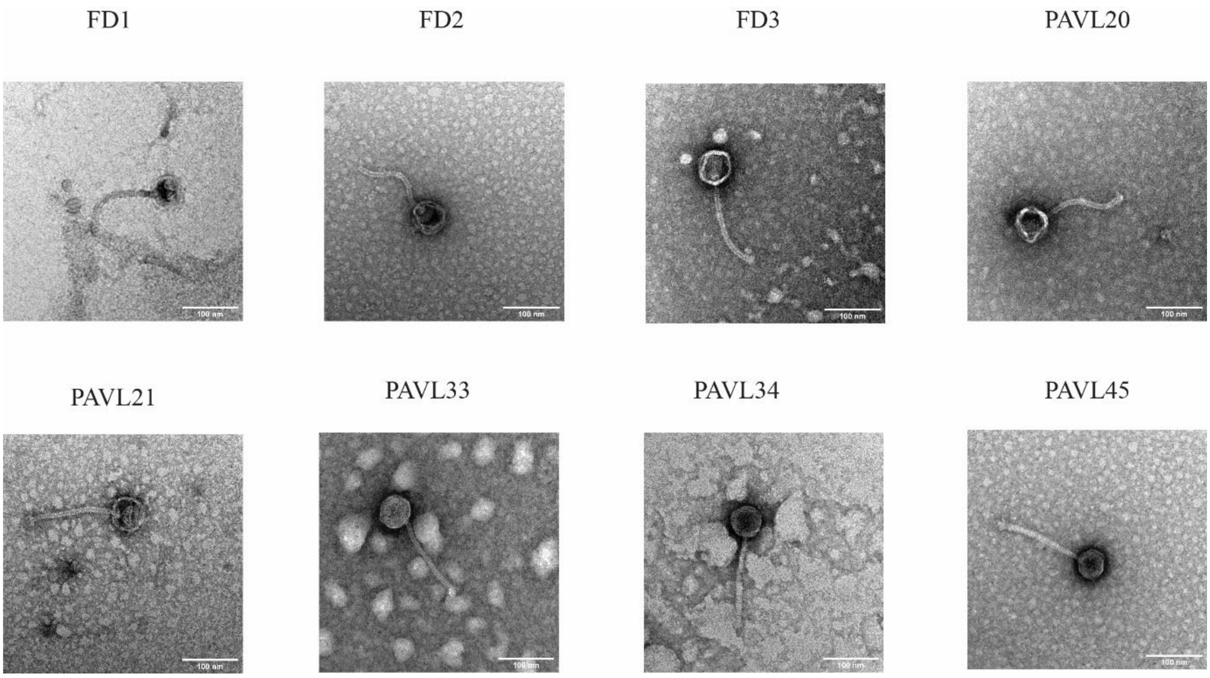

Fig. S1. Transmission electron microscopy (TEM) at ×40,000 magnification of the phages isolated in this work. Tail length and capsid diameter measurements are presented in Table 2. This experiment was done twice.

Fig. S2.

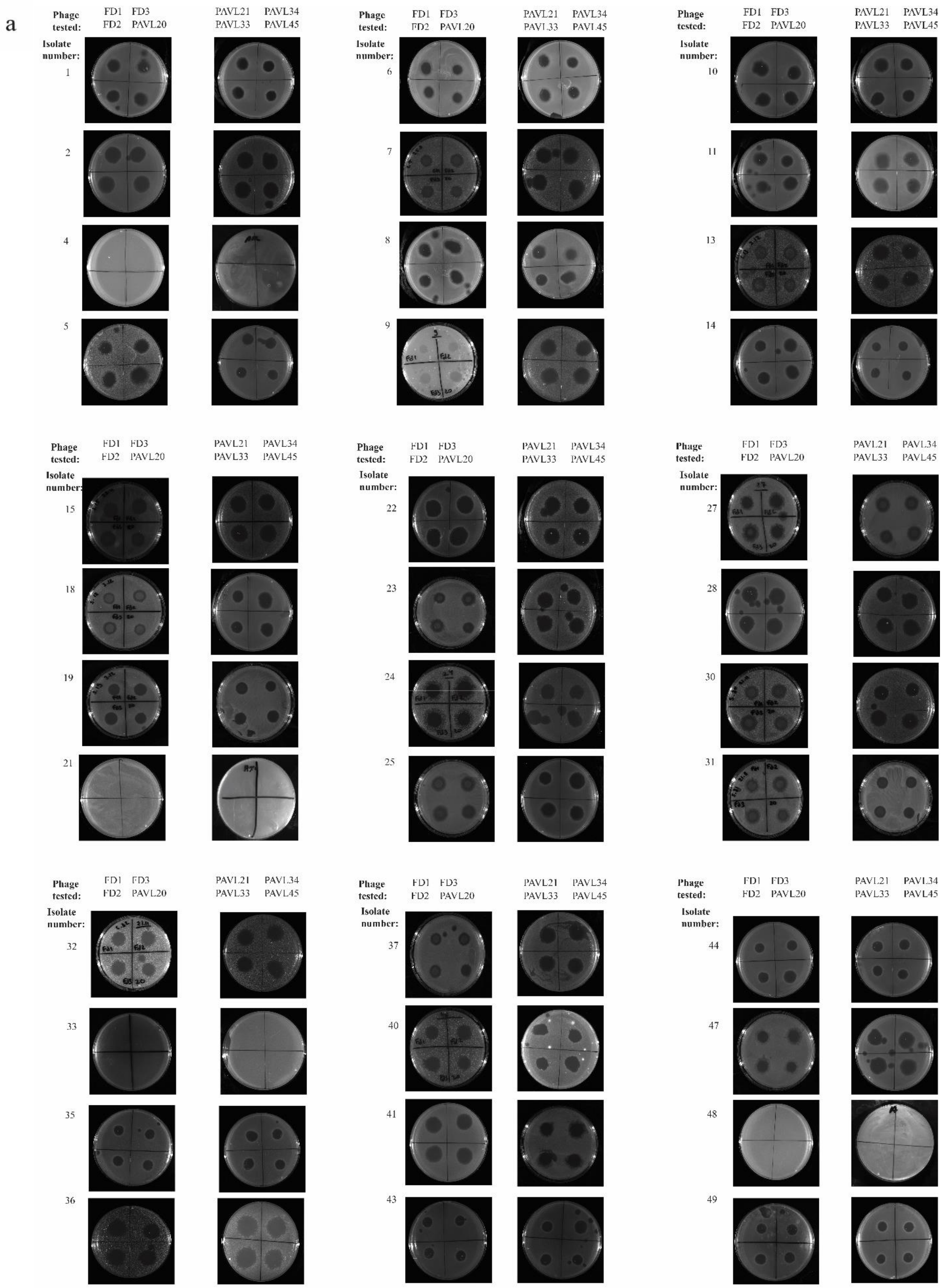
a
Phage tested:
FD1 FD3
FD2 PAVL20
PAVL21 PAVL34
PAVL33 PAVL45
Isolate number:
1
2
4
5
6
7
8
9
10
11
13
14
15
18
19
21
22
23
24
25
27
28
30
31
32
33
35
36
37
40
41
43
44
47
48
49

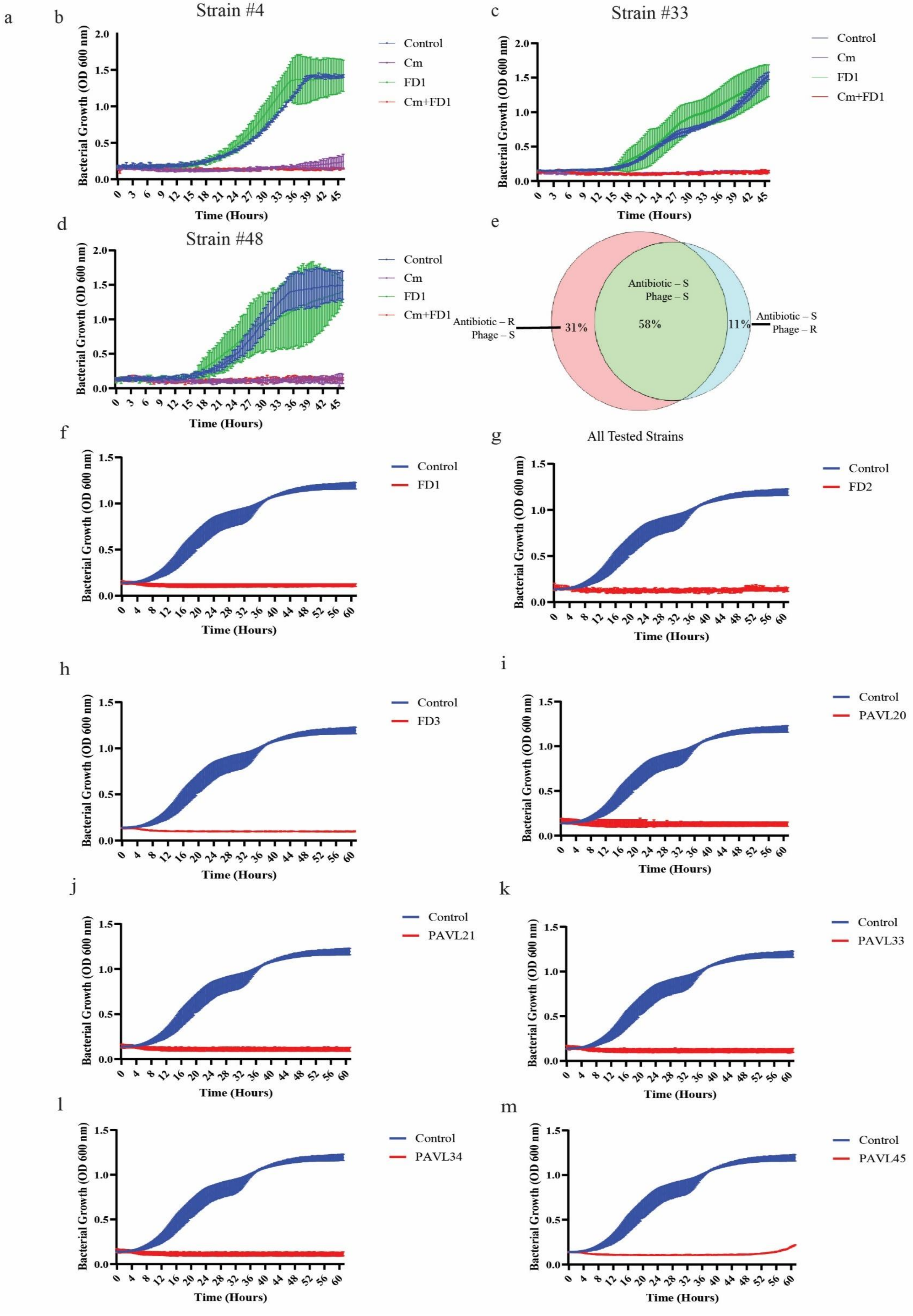
a
b
Strain #4
Bacterial Growth (OD 600 nm)
Time (Hours)
Control
Cm
FD1
Cm+FD1
c
Strain #33
Bacterial Growth (OD 600 nm)
Time (Hours)
Control
Cm
FD1
Cm+FD1
d
Strain #48
Bacterial Growth (OD 600 nm)
Time (Hours)
Control
Cm
FD1
Cm+FD1
e
Antibiotic – S
Phage – S
58%
Antibiotic – R
Phage – S
31%
Antibiotic – S
Phage – R
11%
f
Bacterial Growth (OD 600 nm)
Time (Hours)
Control
FD1
g
All Tested Strains
Bacterial Growth (OD 600 nm)
Time (Hours)
Control
FD2
h
Bacterial Growth (OD 600 nm)
Time (Hours)
Control
FD3
i
Bacterial Growth (OD 600 nm)
Time (Hours)
Control
PAVL20
j
Bacterial Growth (OD 600 nm)
Time (Hours)
Control
PAVL21
k
Bacterial Growth (OD 600 nm)
Time (Hours)
Control
PAVL33
l
Bacterial Growth (OD 600 nm)
Time (Hours)
Control
PAVL34
m
Bacterial Growth (OD 600 nm)
Time (Hours)
Control
PAVL45

Fig S2. Determination of phage sensitivity. (a) The eight phages were spotted on lawns of 36 *C. acnes* isolates (4 phages per plate). (b-d) Kinetics of the phage-resistant strains in the presence of a representative phage (FD1) and antibiotic (Clindamycin, CM) and their combination. (e) Venn diagram of the phage and antibiotic susceptibility. S – susceptible, R – resistant. Note that no strain was resistant to both antibiotics and phages. (F-M) Kinetics of each of the phages grown with a selected sensitive *C. acnes* strain (#27) the strain that was used for the animal experiments. The results are the average of triplicates, presented as mean ± SD. This experiment was done twice. Source data are provided as a Source Data file.

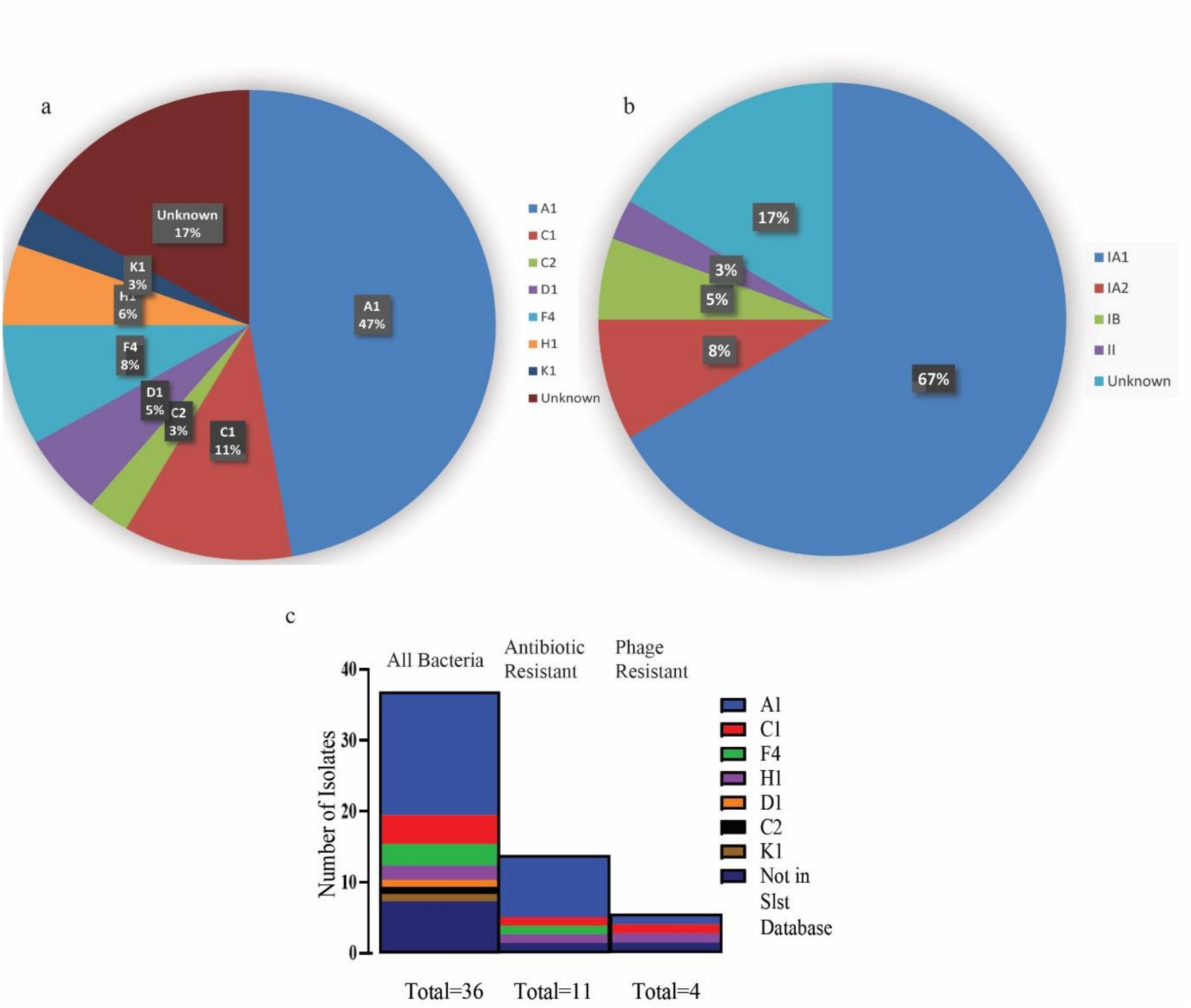

Fig. S3. *C. acnes* Typing. Distribution of the 36 clinical *C. acnes* isolates by (a) SLST types and (b) Phylotypes (traditional typing). (c) SLST type distribution by antibiotic and phage resistance phenotype. data are provided in Supplementary Table S3.

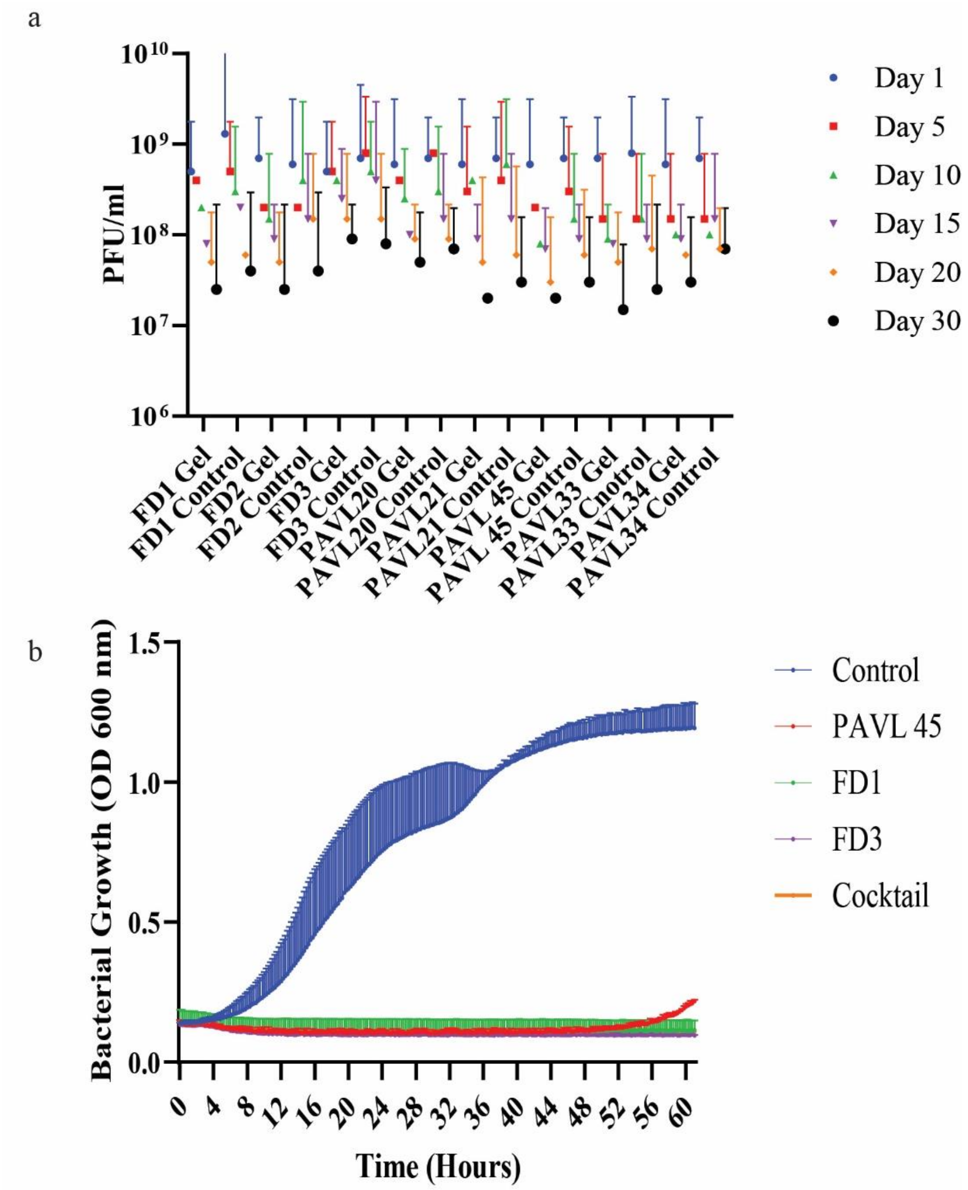

Fig. S4. Phage selection for *in-vivo* experiments phage stability and efficacy were assessed to select one phage, or a phage cocktail for *in-vivo Cutibacterium acnes*-induced acne-like lesions model. (a) Stability in the gel. The eight phages' activity was assessed following incubation in Carbopol gel for 30 days, with samples taken on days 1, 5, 10, 15, 20, and 30. Each day, PFU was calculated from a gel tube and a matching control tube containing a phage in Wilkins medium. (b) Comparison of a single phage activity using FD1, FD3, and PAVL45 vs. a cocktail containing all three phages on *C. acnes* strain #27. Based on these experiments, FD3 was selected as a single phage for the *in-vivo* experiments. Data are presented as mean ± standard deviation (SD). This experiment was done twice. Source data are provided as a Source Data file.

Fig. S5

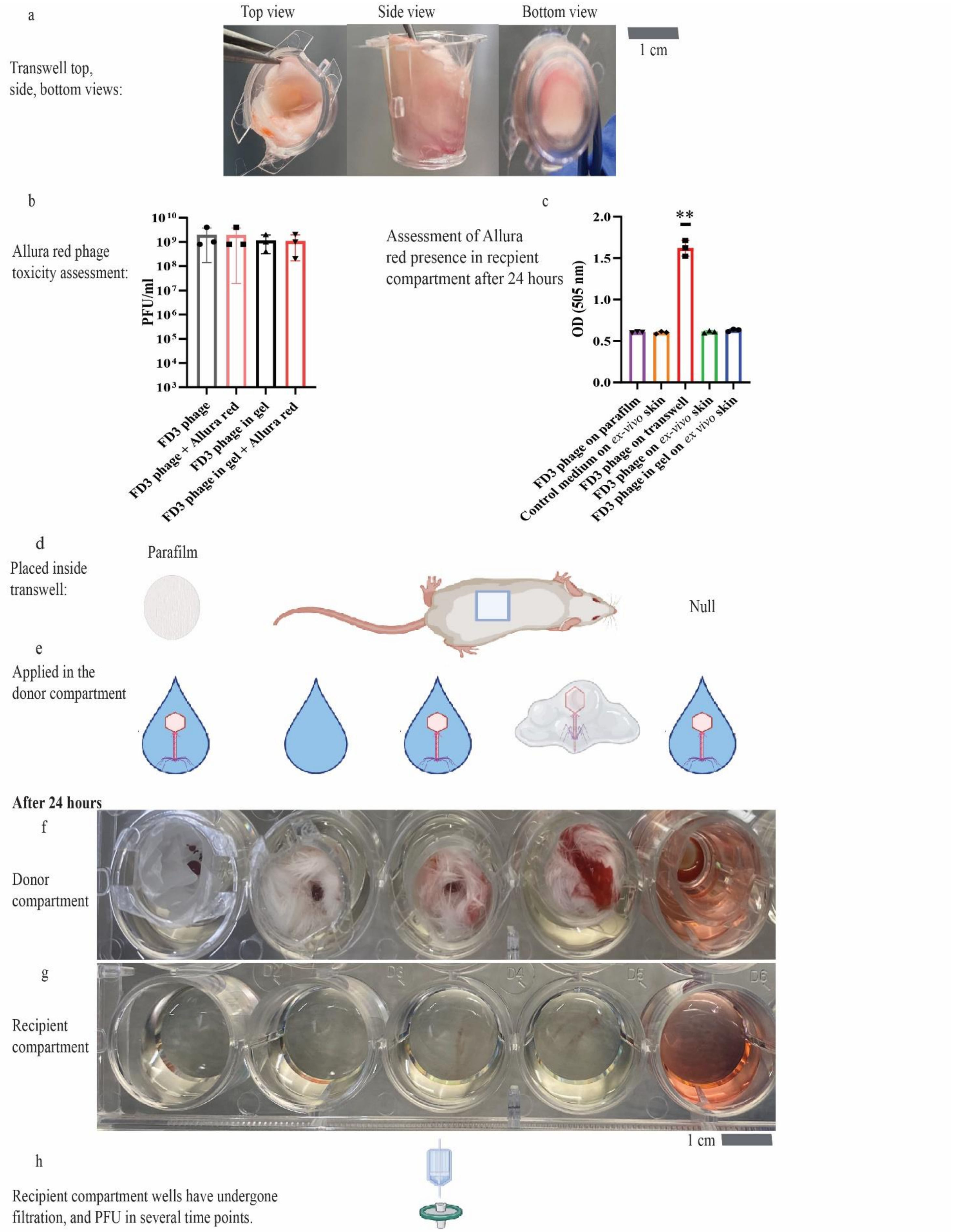
a
Top view
Side view
Bottom view
1 cm
Transwell top, side, bottom views:
b
Allura red phage toxicity assessment:
PFU/ml
FD3 phage
FD3 phage + Allura red
FD3 phage in gel
FD3 phage in gel + Allura red
c
Assessment of Allura red presence in recpient compartment after 24 hours
OD (505 nm)
**
FD3 phage on parafilm
Control medium on ex-vivo skin
FD3 phage on transwell
FD3 phage on ex-vivo skin
FD3 phage in gel on ex vivo skin
d
Placed inside transwell:
Parafilm
Null
e
Applied in the donor compartment
After 24 hours
f
Donor compartment
g
Recipient compartment
1 cm
h
Recipient compartment wells have undergone filtration, and PFU in several time points.

i

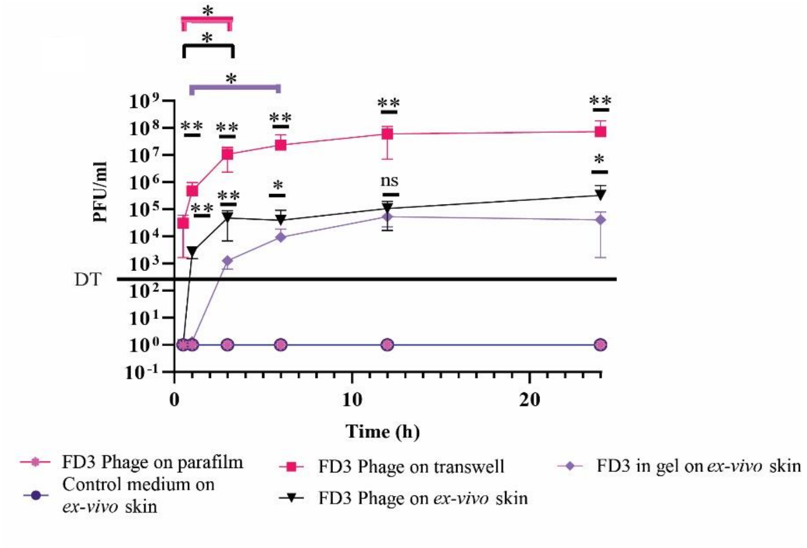

Fig. S5. Assessment of FD3 phages' ability to penetrate intact skin of mice. Phage penetration assessment was done based on the previously described transwell system a version of Franz Diffusion Cell method [1] (a) Healthy full-thickness skin fragments were harvested from mice and put inside 0.4µm transwells. (b) FD3 phage in Wilkins medium, FD3 phage in Carbapol gel, or control Wilkins medium were all supplied with 15 mg/ml Allura Red dye and toxicity was evaluated by comparing PFU counts after 24 hours. *p*-value of FD3 phage in comparison FD3 phage + Allura red = 0.96, and *p*-value of FD3 phage in gel in comparison FD3 phage in gel + Allura red = 0.92 (c) After 24 hours, optical density (OD) from the medium of the recipient compartment was measured. *p*-value of FD3 phage on transwell is < 0.001 in comparison to all other groups. (d) Parafilm/null were put in the control wells. (e-f) 10µl of phage, control or gel were applied in the center of the donor compartment. (g-i) The liquid medium from the recipient compartment was filtered through a 0.22 µm filter, and PFU was assessed at several time points (0.5, 1, 3, 6, 12, and 24 hours). The curves of "FD3 Phage on parafilm" and "Control medium on *ex-vivo* skin" overlap. The results are the average of triplicates, presented as mean ± SD. Student's t-test two-tailed unpaired was used DT stands for Detection Threshold. After 24 hours, *p*-value of FD3 phage on *ex vivo* skin was <0.001 in comparison to phage on either parafilm or transwell, and = 0.004 in comparison to FD3 phage on *ex vivo* skin. * denotes *p*-value< 0.05, and ** denotes *p*-value< 0.001. This experiment was done twice. Source data are provided as a Source Data file. (This figure was prepared using biorender.com)

Characteristic	Value	Score (AU)	Example
Diameter	0-1.9 mm	0	
	2-3.9 mm	1	
	4-5.9 mm	2	
	6-8 mm = 3	3	
Elevation (Double Blinded)	Flat	0	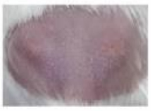
	Small	1	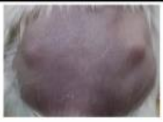
	Large	2	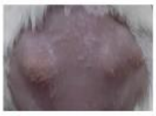
Eschar (Double Blinded)	No	0	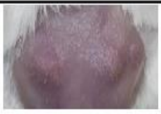
	Mild	1	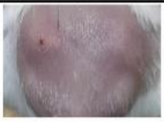
	Marked	2	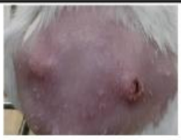
Histology (Based on pathologists' analysis)	Within Normal Range	0	See Supplemental Fig. S7
	Mild	1	
	Moderate	2	
	Severe (Central Necrosis)	3	

Fig. S6. The clinical scoring of mice that was used in this work. Diameter, elevation, and eschar were used to score lesion's severity. The total clinical score of the lesions was calculated by summing up the clinical parameters on a scale between 0-7 (7 most severe). The overall combined histological and clinical score was calculated as the sum of the average histological score (Supplemental Fig. S7) and the total clinical score. For more details, please refer to Fig. 3, Supplemental Table S4, and the phage clinical efficacy subsection assessment in the Materials and Methods.

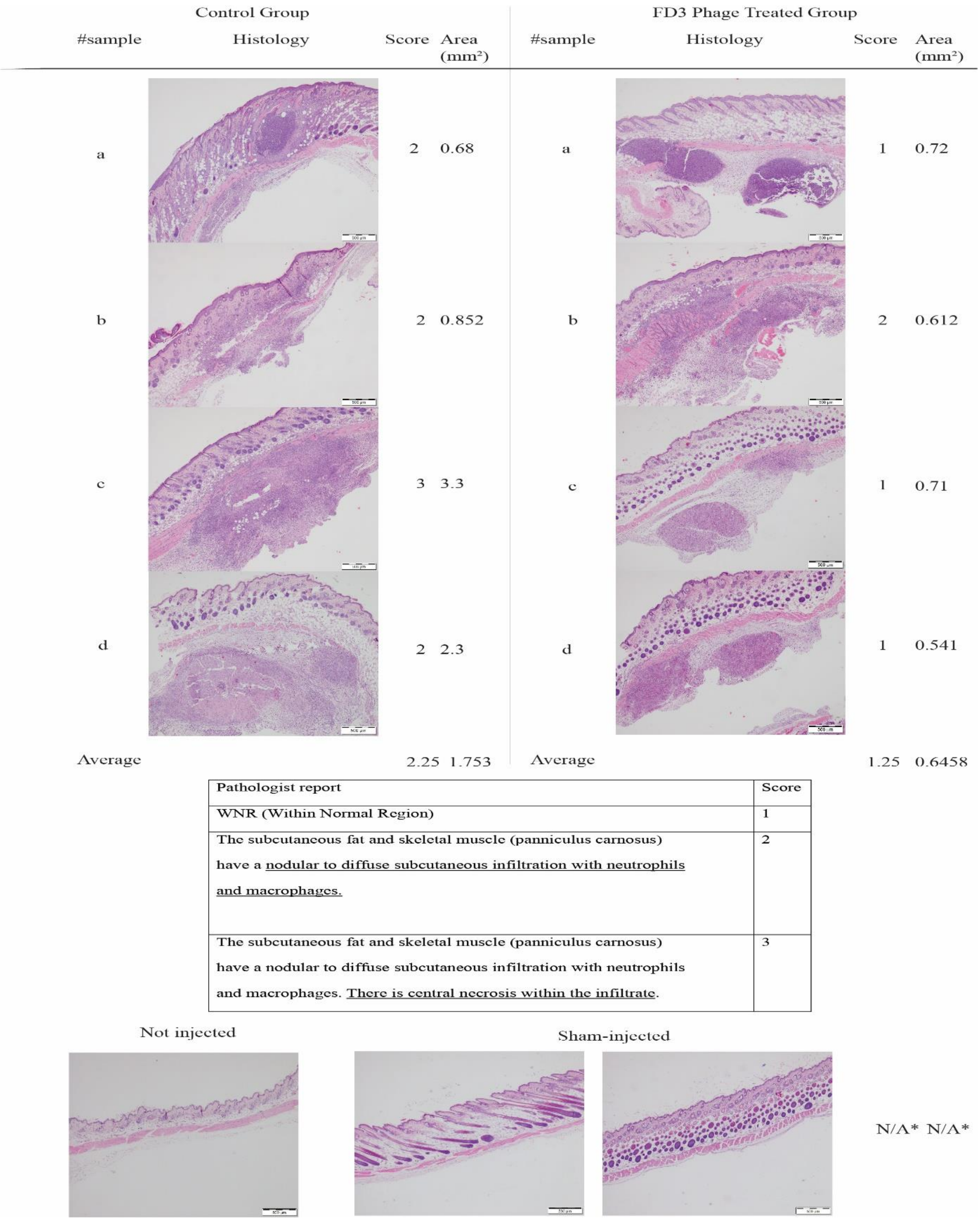

Control Group				FD3 Phage Treated Group			
#sample	Histology	Score	Area (mm²)	#sample	Histology	Score	Area (mm²)
a		2	0.68	a		1	0.72
b		2	0.852	b		2	0.612
c		3	3.3	c		1	0.71
d		2	2.3	d		1	0.541
Average		2.25	1.753	Average		1.25	0.6458

Pathologist report	Score
WNR (Within Normal Region)	1
The subcutaneous fat and skeletal muscle (panniculus carnosus) have a nodular to diffuse subcutaneous infiltration with neutrophils and macrophages.	2
The subcutaneous fat and skeletal muscle (panniculus carnosus) have a nodular to diffuse subcutaneous infiltration with neutrophils and macrophages. There is central necrosis within the infiltrate.	3

Fig. S7. Histological score. The score was created based on four biopsies from each group of mice in ×4 magnification. The area was calculated graphically using ImageJ software (1.53), and a score was calculated based on the pathological report. Averaging and combining the histological and clinical scores resulted in the combined histological and clinical score (See also Fig. 4). *Not-infected and sham-injected controls were not scored as no lesion was detected. This experiment was done twice.

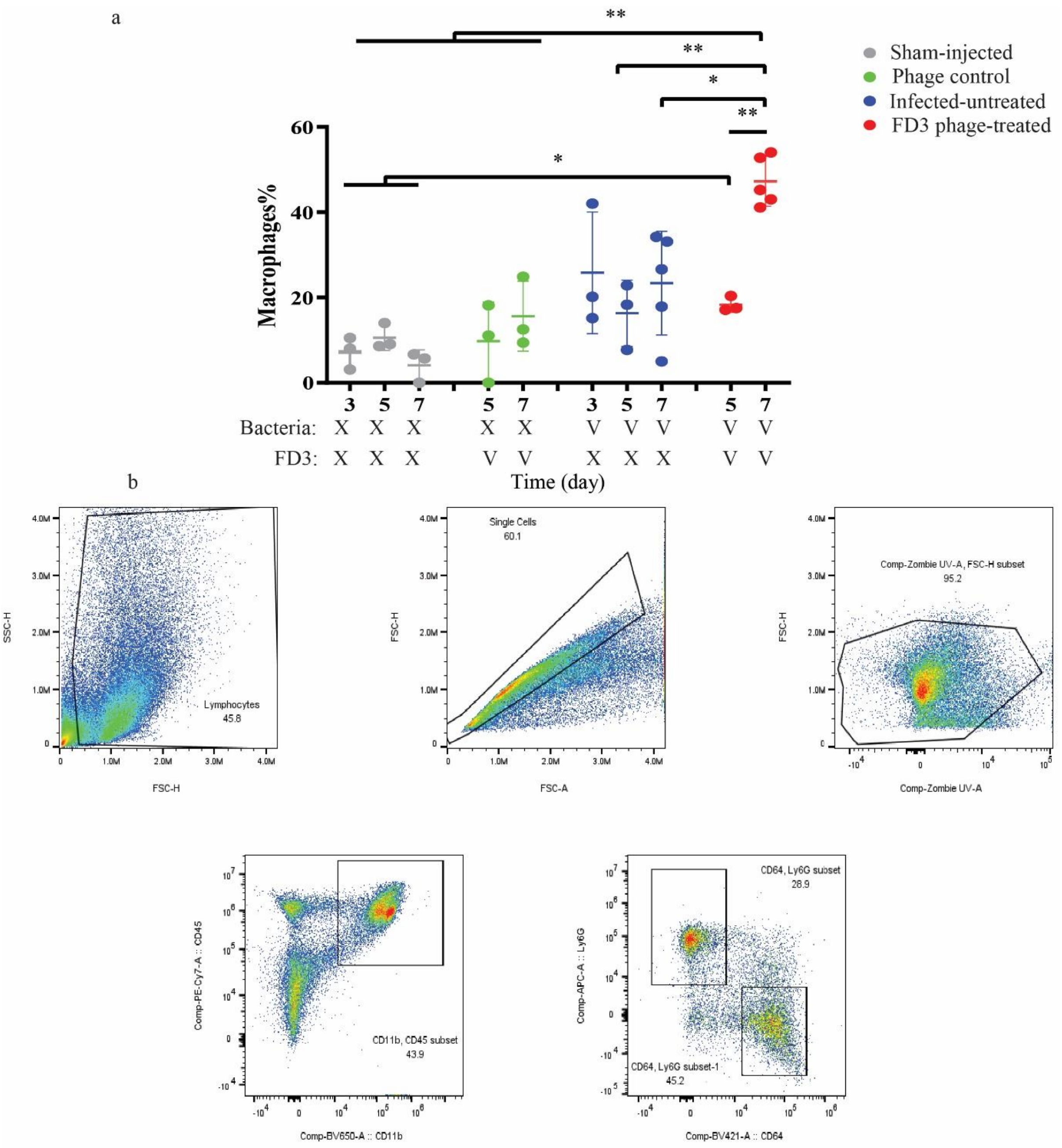

Fig. S8. Flow cytometry of macrophages from the *C. acnes*-induced lesions at various time points. (a) Percent of macrophages in mice lesions. On day 3, mice were allocated to treatment or vehicle groups, therefore there are no treated mice at this time point. The results are presented as mean ± SD. *p*-value of the infected-untreated group in comparison to the phage treated group =0.61, 0.01 on day 5, 7 accordingly. n = between 3-5 mice as described in Table 4. Student's t-test two-tailed unpaired was used, * denotes *p*-value< 0.05, and ** denotes *p*-value< 0.001. Source data are provided as a Source Data file. (b) Gating strategy of $CD45^{+}CD11b^{+}Ly6G^{+}$ [2] (neutrophils) and $CD45^{+}CD11b^{+}CD64^{+}$ [3] (macrophages) and flow cytometry experiments.

Table S1.

The genome sequence details of the phages used to construct the phylogenetic tree which is depicted in Fig. 1a. This table was created using the Genious Prime 2022.2.1 package.

Table S1.

Index	Accession	Description	Sequence length	Taxonomy
1	DQ431235	Propionibacterium phage PA6, complete genome	29739	Viruses; Duplodnaviria; Heunggongvirae; Uroviricota; Caudoviricetes; Caudovirales; Siphoviridae; Pahexavirus
2	FJ706171	Propionibacterium phage PAD20, complete genome	29074	Viruses; Duplodnaviria; Heunggongvirae; Uroviricota; Caudoviricetes; Caudovirales; Siphoviridae; Pahexavirus
3	FJ706172	Propionibacterium phage PAS50, complete genome	29017	Viruses; Duplodnaviria; Heunggongvirae; Uroviricota; Caudoviricetes; Caudovirales; Siphoviridae; Pahexavirus
4	JX262215	Propionibacterium phage P9.1, complete genome	29214	Viruses; Duplodnaviria; Heunggongvirae; Uroviricota; Caudoviricetes; Caudovirales; Siphoviridae; Pahexavirus
5	JX262216	Propionibacterium phage P14.4, complete genome	29729	Viruses; Duplodnaviria; Heunggongvirae; Uroviricota; Caudoviricetes; Caudovirales; Siphoviridae; Pahexavirus
6	JX262217	Propionibacterium phage P101A, complete genome	29574	Viruses; Duplodnaviria; Heunggongvirae; Uroviricota; Caudoviricetes; Caudovirales; Siphoviridae; Pahexavirus
7	JX262218	Propionibacterium phage P104A, complete genome	29371	Viruses; Duplodnaviria; Heunggongvirae; Uroviricota; Caudoviricetes; Caudovirales; Siphoviridae; Pahexavirus
8	JX262219	Propionibacterium phage P105, complete genome	29202	Viruses; Duplodnaviria; Heunggongvirae; Uroviricota; Caudoviricetes; Caudovirales; Siphoviridae; Pahexavirus
9	JX262220	Propionibacterium phage P100D, complete genome	29506	Viruses; Duplodnaviria; Heunggongvirae; Uroviricota; Caudoviricetes; Caudovirales; Siphoviridae; Pahexavirus
10	JX262221	Propionibacterium phage P100_A, complete genome	29505	Viruses; Duplodnaviria; Heunggongvirae; Uroviricota; Caudoviricetes; Caudovirales; Siphoviridae; Pahexavirus
11	JX262222	Propionibacterium phage P100_1, complete genome	29612	Viruses; Duplodnaviria; Heunggongvirae; Uroviricota; Caudoviricetes; Caudovirales; Siphoviridae; Pahexavirus
12	JX262223	Propionibacterium phage P1.1, complete genome	29348	Viruses; Duplodnaviria; Heunggongvirae; Uroviricota; Caudoviricetes; Caudovirales; Siphoviridae; Pahexavirus
13	JX262224	Propionibacterium phage ATCC29399B_T, complete geno	29516	Viruses; Duplodnaviria; Heunggongvirae; Uroviricota; Caudoviricetes; Caudovirales; Siphoviridae; Pahexavirus
14	JX262225	Propionibacterium phage ATCC29399B_C, complete geno	29516	Viruses; Duplodnaviria; Heunggongvirae; Uroviricota; Caudoviricetes; Caudovirales; Siphoviridae; Pahexavirus
15	JX570702	Propionibacterium phage PHL111M01, complete genome	29140	Viruses; Duplodnaviria; Heunggongvirae; Uroviricota; Caudoviricetes; Caudovirales; Siphoviridae; Pahexavirus
16	JX570703	Propionibacterium phage PHL073M02, complete genome	29503	Viruses; Duplodnaviria; Heunggongvirae; Uroviricota; Caudoviricetes; Caudovirales; Siphoviridae; Pahexavirus; unclassified Pahexavirus
17	JX570704	Propionibacterium phage PHL010M04, complete genome	29511	Viruses; Duplodnaviria; Heunggongvirae; Uroviricota; Caudoviricetes; Caudovirales; Siphoviridae; Pahexavirus; unclassified Pahexavirus
18	JX570705	Propionibacterium phage PHL060L00, complete genome	29514	Viruses; Duplodnaviria; Heunggongvirae; Uroviricota; Caudoviricetes; Caudovirales; Siphoviridae; Pahexavirus
19	JX570706	Propionibacterium phage PHL037M02, complete genome	29443	Viruses; Duplodnaviria; Heunggongvirae; Uroviricota; Caudoviricetes; Caudovirales; Siphoviridae; Pahexavirus

20	JX570707	Propionibacterium phage PHL085M01, complete genome	29451	Viruses; Duplodnaviria; Heunggongvirae; Uroviricota; Caudoviricetes; Caudovirales; Siphoviridae; Pahexavirus; unclassified Pahexavirus
21	JX570708	Propionibacterium phage PHL115M02, complete genome	29453	Viruses; Duplodnaviria; Heunggongvirae; Uroviricota; Caudoviricetes; Caudovirales; Siphoviridae; Pahexavirus; unclassified Pahexavirus
22	JX570709	Propionibacterium phage PHL067M10, complete genome	29377	Viruses; Duplodnaviria; Heunggongvirae; Uroviricota; Caudoviricetes; Caudovirales; Siphoviridae; Pahexavirus; unclassified Pahexavirus
23	JX570710	Propionibacterium phage PHL071N05, complete genome	29467	Viruses; Duplodnaviria; Heunggongvirae; Uroviricota; Caudoviricetes; Caudovirales; Siphoviridae; Pahexavirus
24	JX570711	Propionibacterium phage PHL066M04, complete genome	29512	Viruses; Duplodnaviria; Heunggongvirae; Uroviricota; Caudoviricetes; Caudovirales; Siphoviridae; Pahexavirus; unclassified Pahexavirus
25	JX570712	Propionibacterium phage PHL114L00, complete genome	29464	Viruses; Duplodnaviria; Heunggongvirae; Uroviricota; Caudoviricetes; Caudovirales; Siphoviridae; Pahexavirus
26	JX570713	Propionibacterium phage PHL113M01, complete genome	29200	Viruses; Duplodnaviria; Heunggongvirae; Uroviricota; Caudoviricetes; Caudovirales; Siphoviridae; Pahexavirus
27	JX570714	Propionibacterium phage PHL112N00, complete genome	29266	Viruses; Duplodnaviria; Heunggongvirae; Uroviricota; Caudoviricetes; Caudovirales; Siphoviridae; Pahexavirus
28	KJ578758	Propionibacterium phage PHL009M11, complete genome	29503	Viruses; Duplodnaviria; Heunggongvirae; Uroviricota; Caudoviricetes; Caudovirales; Siphoviridae; Pahexavirus
29	KJ578759	Propionibacterium phage PHL025M00, complete genome	29496	Viruses; Duplodnaviria; Heunggongvirae; Uroviricota; Caudoviricetes; Caudovirales; Siphoviridae; Pahexavirus
30	KJ578760	Propionibacterium phage PHL030N00, complete genome	29423	Viruses; Duplodnaviria; Heunggongvirae; Uroviricota; Caudoviricetes; Caudovirales; Siphoviridae; Pahexavirus; unclassified Pahexavirus
31	KJ578761	Propionibacterium phage PHL041M10, complete genome	29412	Viruses; Duplodnaviria; Heunggongvirae; Uroviricota; Caudoviricetes; Caudovirales; Siphoviridae; Pahexavirus
32	KJ578762	Propionibacterium phage PHL055N00, complete genome	29264	Viruses; Duplodnaviria; Heunggongvirae; Uroviricota; Caudoviricetes; Caudovirales; Siphoviridae; Pahexavirus; unclassified Pahexavirus
33	KJ578763	Propionibacterium phage PHL064M01, complete genome	29424	Viruses; Duplodnaviria; Heunggongvirae; Uroviricota; Caudoviricetes; Caudovirales; Siphoviridae; Pahexavirus; unclassified Pahexavirus
34	KJ578764	Propionibacterium phage PHL064M02, complete genome	29407	Viruses; Duplodnaviria; Heunggongvirae; Uroviricota; Caudoviricetes; Caudovirales; Siphoviridae; Pahexavirus; unclassified Pahexavirus
35	KJ578765	Propionibacterium phage PHL067M01, complete genome	29377	Viruses; Duplodnaviria; Heunggongvirae; Uroviricota; Caudoviricetes; Caudovirales; Siphoviridae; Pahexavirus
36	KJ578766	Propionibacterium phage PHL067M09, complete genome	29386	Viruses; Duplodnaviria; Heunggongvirae; Uroviricota; Caudoviricetes; Caudovirales; Siphoviridae; Pahexavirus; unclassified Pahexavirus
37	KJ578767	Propionibacterium phage PHL070N00, complete genome	29421	Viruses; Duplodnaviria; Heunggongvirae; Uroviricota; Caudoviricetes; Caudovirales; Siphoviridae; Pahexavirus
38	KJ578768	Propionibacterium phage PHL082M00, complete genome	29491	Viruses; Duplodnaviria; Heunggongvirae; Uroviricota; Caudoviricetes; Caudovirales; Siphoviridae; Pahexavirus; unclassified Pahexavirus
39	KJ578769	Propionibacterium phage PHL082M02, complete genome	29491	Viruses; Duplodnaviria; Heunggongvirae; Uroviricota; Caudoviricetes; Caudovirales; Siphoviridae; Pahexavirus; unclassified Pahexavirus
40	KJ578770	Propionibacterium phage PHL082M03, complete genome	29491	Viruses; Duplodnaviria; Heunggongvirae; Uroviricota; Caudoviricetes; Caudovirales; Siphoviridae; Pahexavirus
41	KJ578771	Propionibacterium phage PHL082M04, complete genome	29491	Viruses; Duplodnaviria; Heunggongvirae; Uroviricota; Caudoviricetes; Caudovirales; Siphoviridae; Pahexavirus; unclassified Pahexavirus

42	KJ578772	Propionibacterium phage PHL085N00, complete genome	29454	Viruses; Duplodnaviria; Heunggongvirae; Uroviricota; Caudoviricetes; Caudovirales; Siphoviridae; Pahexavirus; unclassified Pahexavirus
43	KJ578773	Propionibacterium phage PHL092M00, complete genome	29261	Viruses; Duplodnaviria; Heunggongvirae; Uroviricota; Caudoviricetes; Caudovirales; Siphoviridae; Pahexavirus
44	KJ578774	Propionibacterium phage PHL095N00, complete genome	29751	Viruses; Duplodnaviria; Heunggongvirae; Uroviricota; Caudoviricetes; Caudovirales; Siphoviridae; Pahexavirus
45	KJ578775	Propionibacterium phage PHL114N00, complete genome	29464	Viruses; Duplodnaviria; Heunggongvirae; Uroviricota; Caudoviricetes; Caudovirales; Siphoviridae; Pahexavirus
46	KJ578776	Propionibacterium phage PHL116M00, complete genome	29394	Viruses; Duplodnaviria; Heunggongvirae; Uroviricota; Caudoviricetes; Caudovirales; Siphoviridae; Pahexavirus
47	KJ578777	Propionibacterium phage PHL116M10, complete genome	29396	Viruses; Duplodnaviria; Heunggongvirae; Uroviricota; Caudoviricetes; Caudovirales; Siphoviridae; Pahexavirus; unclassified Pahexavirus
48	KJ578778	Propionibacterium phage PHL117M00, complete genome	29255	Viruses; Duplodnaviria; Heunggongvirae; Uroviricota; Caudoviricetes; Caudovirales; Siphoviridae; Pahexavirus
49	KJ578779	Propionibacterium phage PHL117M01, complete genome	29422	Viruses; Duplodnaviria; Heunggongvirae; Uroviricota; Caudoviricetes; Caudovirales; Siphoviridae; Pahexavirus
50	KJ578780	Propionibacterium phage PHL132N00, complete genome	29003	Viruses; Duplodnaviria; Heunggongvirae; Uroviricota; Caudoviricetes; Caudovirales; Siphoviridae; Pahexavirus
51	KJ578781	Propionibacterium phage PHL141N00, complete genome	29494	Viruses; Duplodnaviria; Heunggongvirae; Uroviricota; Caudoviricetes; Caudovirales; Siphoviridae; Pahexavirus
52	KJ578782	Propionibacterium phage PHL150M00, complete genome	29438	Viruses; Duplodnaviria; Heunggongvirae; Uroviricota; Caudoviricetes; Caudovirales; Siphoviridae; Pahexavirus; unclassified Pahexavirus
53	KJ578783	Propionibacterium phage PHL151M00, complete genome	29511	Viruses; Duplodnaviria; Heunggongvirae; Uroviricota; Caudoviricetes; Caudovirales; Siphoviridae; Pahexavirus
54	KJ578784	Propionibacterium phage PHL151N00, complete genome	29511	Viruses; Duplodnaviria; Heunggongvirae; Uroviricota; Caudoviricetes; Caudovirales; Siphoviridae; Pahexavirus
55	KJ578785	Propionibacterium phage PHL152M00, complete genome	29247	Viruses; Duplodnaviria; Heunggongvirae; Uroviricota; Caudoviricetes; Caudovirales; Siphoviridae; Pahexavirus
56	KJ578786	Propionibacterium phage PHL163M00, complete genome	29264	Viruses; Duplodnaviria; Heunggongvirae; Uroviricota; Caudoviricetes; Caudovirales; Siphoviridae; Pahexavirus
57	KJ578787	Propionibacterium phage PHL171M01, complete genome	29327	Viruses; Duplodnaviria; Heunggongvirae; Uroviricota; Caudoviricetes; Caudovirales; Siphoviridae; Pahexavirus
58	KJ578788	Propionibacterium phage PHL179M00, complete genome	29428	Viruses; Duplodnaviria; Heunggongvirae; Uroviricota; Caudoviricetes; Caudovirales; Siphoviridae; Pahexavirus
59	KJ578789	Propionibacterium phage PHL194M00, complete genome	29264	Viruses; Duplodnaviria; Heunggongvirae; Uroviricota; Caudoviricetes; Caudovirales; Siphoviridae; Pahexavirus
60	KJ578790	Propionibacterium phage PHL199M00, complete genome	29806	Viruses; Duplodnaviria; Heunggongvirae; Uroviricota; Caudoviricetes; Caudovirales; Siphoviridae; Pahexavirus
61	KJ578791	Propionibacterium phage PHL301M00, complete genome	29323	Viruses; Duplodnaviria; Heunggongvirae; Uroviricota; Caudoviricetes; Caudovirales; Siphoviridae; Pahexavirus
62	KJ578792	Propionibacterium phage PHL308M00, complete genome	29442	Viruses; Duplodnaviria; Heunggongvirae; Uroviricota; Caudoviricetes; Caudovirales; Siphoviridae; Pahexavirus
63	KJ722067	Propionibacterium phage Pacnes 2012-15, complete geno	29741	Viruses; Duplodnaviria; Heunggongvirae; Uroviricota; Caudoviricetes; Caudovirales; Siphoviridae; Pahexavirus

64	KR337643	Propionibacterium phage MrAK, complete genome	29726	Viruses; Duplodnaviria; Heunggongvirae; Uroviricota; Caudoviricetes; Caudovirales; Siphoviridae; Pahexavirus
65	KR337644	Propionibacterium phage Procrass1, complete genome	29347	Viruses; Duplodnaviria; Heunggongvirae; Uroviricota; Caudoviricetes; Caudovirales; Siphoviridae; Pahexavirus
66	KR337645	Propionibacterium phage Kubed, complete genome	29461	Viruses; Duplodnaviria; Heunggongvirae; Uroviricota; Caudoviricetes; Caudovirales; Siphoviridae; Pahexavirus
67	KR337646	Propionibacterium phage Wizzo, complete genome	29463	Viruses; Duplodnaviria; Heunggongvirae; Uroviricota; Caudoviricetes; Caudovirales; Siphoviridae; Pahexavirus
68	KR337647	Propionibacterium phage Solid, complete genome	29440	Viruses; Duplodnaviria; Heunggongvirae; Uroviricota; Caudoviricetes; Caudovirales; Siphoviridae; Pahexavirus
69	KR337648	Propionibacterium phage SKKY, complete genome	29594	Viruses; Duplodnaviria; Heunggongvirae; Uroviricota; Caudoviricetes; Caudovirales; Siphoviridae; Pahexavirus
70	KR337649	Propionibacterium phage Keiki, complete genome	29339	Viruses; Duplodnaviria; Heunggongvirae; Uroviricota; Caudoviricetes; Caudovirales; Siphoviridae; Pahexavirus
71	KR337650	Propionibacterium phage Lauchelly, complete genome	29517	Viruses; Duplodnaviria; Heunggongvirae; Uroviricota; Caudoviricetes; Caudovirales; Siphoviridae; Pahexavirus
72	KR337651	Propionibacterium phage Attacne, complete genome	28876	Viruses; Duplodnaviria; Heunggongvirae; Uroviricota; Caudoviricetes; Caudovirales; Siphoviridae; Pahexavirus
73	KR337652	Propionibacterium phage Stormborn, complete genome	29330	Viruses; Duplodnaviria; Heunggongvirae; Uroviricota; Caudoviricetes; Caudovirales; Siphoviridae; Pahexavirus
74	KR337653	Propionibacterium phage Pirate, complete genome	29324	Viruses; Duplodnaviria; Heunggongvirae; Uroviricota; Caudoviricetes; Caudovirales; Siphoviridae; Pahexavirus
75	KR337654	Propionibacterium phage Ouroboros, complete genome	29506	Viruses; Duplodnaviria; Heunggongvirae; Uroviricota; Caudoviricetes; Caudovirales; Siphoviridae; Pahexavirus
76	KR347352	Propionibacterium phage BruceLethal, complete genome	29249	Viruses; Duplodnaviria; Heunggongvirae; Uroviricota; Caudoviricetes; Caudovirales; Siphoviridae; Pahexavirus; unclassified Pahexavirus
77	KR347353	Propionibacterium phage Enoki, complete genome	29347	Viruses; Duplodnaviria; Heunggongvirae; Uroviricota; Caudoviricetes; Caudovirales; Siphoviridae; Pahexavirus; unclassified Pahexavirus
78	KR347354	Propionibacterium phage Moyashi, complete genome	29254	Viruses; Duplodnaviria; Heunggongvirae; Uroviricota; Caudoviricetes; Caudovirales; Siphoviridae; Pahexavirus; unclassified Pahexavirus
79	KR347355	Propionibacterium phage QueenBey, complete genome	29338	Viruses; Duplodnaviria; Heunggongvirae; Uroviricota; Caudoviricetes; Caudovirales; Siphoviridae; Pahexavirus; unclassified Pahexavirus
80	KR902978	Propionibacterium phage PAC1, complete genome	29605	Viruses; Duplodnaviria; Heunggongvirae; Uroviricota; Caudoviricetes; Caudovirales; Siphoviridae; Pahexavirus; unclassified Pahexavirus
81	KR902979	Propionibacterium phage PAC2, complete genome	29602	Viruses; Duplodnaviria; Heunggongvirae; Uroviricota; Caudoviricetes; Caudovirales; Siphoviridae; Pahexavirus; unclassified Pahexavirus
82	KR902980	Propionibacterium phage PAC3, complete genome	29526	Viruses; Duplodnaviria; Heunggongvirae; Uroviricota; Caudoviricetes; Caudovirales; Siphoviridae; Pahexavirus; unclassified Pahexavirus
83	KR902981	Propionibacterium phage PAC4, complete genome	29581	Viruses; Duplodnaviria; Heunggongvirae; Uroviricota; Caudoviricetes; Caudovirales; Siphoviridae; Pahexavirus; unclassified Pahexavirus
84	KR902982	Propionibacterium phage PAC5, complete genome	29428	Viruses; Duplodnaviria; Heunggongvirae; Uroviricota; Caudoviricetes; Caudovirales; Siphoviridae; Pahexavirus; unclassified Pahexavirus
85	KR902983	Propionibacterium phage PAC6, complete genome	29609	Viruses; Duplodnaviria; Heunggongvirae; Uroviricota; Caudoviricetes; Caudovirales; Siphoviridae; Pahexavirus; unclassified Pahexavirus

86	KR902984	Propionibacterium phage PAC7, complete genome	29551	Viruses; Duplodnaviria; Heunggongvirae; Uroviricota; Caudoviricetes; Caudovirales; Siphoviridae; Pahexavirus; unclassified Pahexavirus
87	KR902985	Propionibacterium phage PAC8, complete genome	29488	Viruses; Duplodnaviria; Heunggongvirae; Uroviricota; Caudoviricetes; Caudovirales; Siphoviridae; Pahexavirus; unclassified Pahexavirus
88	KR902986	Propionibacterium phage PAC9, complete genome	29786	Viruses; Duplodnaviria; Heunggongvirae; Uroviricota; Caudoviricetes; Caudovirales; Siphoviridae; Pahexavirus; unclassified Pahexavirus
89	KR902987	Propionibacterium phage PAC10, complete genome	29704	Viruses; Duplodnaviria; Heunggongvirae; Uroviricota; Caudoviricetes; Caudovirales; Siphoviridae; Pahexavirus; unclassified Pahexavirus
90	KT934381	Propionibacterium phage PA1-14, complete genome	29407	Viruses; Duplodnaviria; Heunggongvirae; Uroviricota; Caudoviricetes; Caudovirales; Siphoviridae; Pahexavirus; unclassified Pahexavirus
91	KY926792	Propionibacterium phage PacnesP1, complete genome	29533	Viruses; Duplodnaviria; Heunggongvirae; Uroviricota; Caudoviricetes; Caudovirales; Siphoviridae; Pahexavirus; unclassified Pahexavirus
92	KY926793	Propionibacterium phage PacnesP2, complete genome	30016	Viruses; Duplodnaviria; Heunggongvirae; Uroviricota; Caudoviricetes; Caudovirales; Siphoviridae; Pahexavirus; unclassified Pahexavirus
93	MF919491	Propionibacterium phage Aquarius, complete genome	30112	Viruses; Duplodnaviria; Heunggongvirae; Uroviricota; Caudoviricetes; Caudovirales; Siphoviridae; Pahexavirus; unclassified Pahexavirus
94	MF919505	Propionibacterium phage DrParker, complete genome	29742	Viruses; Duplodnaviria; Heunggongvirae; Uroviricota; Caudoviricetes; Caudovirales; Siphoviridae; Pahexavirus; unclassified Pahexavirus
95	MF919515	Propionibacterium phage Leviosa, complete genome	29451	Viruses; Duplodnaviria; Heunggongvirae; Uroviricota; Caudoviricetes; Caudovirales; Siphoviridae; Pahexavirus; unclassified Pahexavirus
96	MF919516	Propionibacterium phage LilBandit, complete genome	29041	Viruses; Duplodnaviria; Heunggongvirae; Uroviricota; Caudoviricetes; Caudovirales; Siphoviridae; Pahexavirus; unclassified Pahexavirus
97	MF919522	Propionibacterium phage MEAK, complete genome	29223	Viruses; Duplodnaviria; Heunggongvirae; Uroviricota; Caudoviricetes; Caudovirales; Siphoviridae; Pahexavirus; unclassified Pahexavirus
98	MF919533	Propionibacterium phage Supernova, complete genome	29217	Viruses; Duplodnaviria; Heunggongvirae; Uroviricota; Caudoviricetes; Caudovirales; Siphoviridae; Pahexavirus; unclassified Pahexavirus
99	MG820632	Propionibacterium phage pa310, complete genome	29508	Viruses; Duplodnaviria; Heunggongvirae; Uroviricota; Caudoviricetes; Caudovirales; Siphoviridae; Pahexavirus; unclassified Pahexavirus
100	MG820633	Propionibacterium phage pa59, complete genome	29507	Viruses; Duplodnaviria; Heunggongvirae; Uroviricota; Caudoviricetes; Caudovirales; Siphoviridae; Pahexavirus; unclassified Pahexavirus
101	MG820634	Propionibacterium phage pa27, complete genome	29593	Viruses; Duplodnaviria; Heunggongvirae; Uroviricota; Caudoviricetes; Caudovirales; Siphoviridae; Pahexavirus; unclassified Pahexavirus
102	MG820635	Propionibacterium phage pa29399-1-D_1, complete geno	29370	Viruses; Duplodnaviria; Heunggongvirae; Uroviricota; Caudoviricetes; Caudovirales; Siphoviridae; Pahexavirus; unclassified Pahexavirus
103	MG820636	Propionibacterium phage pa29399-1-D_2, complete geno	28892	Viruses; Duplodnaviria; Heunggongvirae; Uroviricota; Caudoviricetes; Caudovirales; Siphoviridae; Pahexavirus; unclassified Pahexavirus
104	MG820637	Propionibacterium phage pa63, complete genome	29446	Viruses; Duplodnaviria; Heunggongvirae; Uroviricota; Caudoviricetes; Caudovirales; Siphoviridae; Pahexavirus; unclassified Pahexavirus
105	MG820638	Propionibacterium phage pa6919-4, complete genome	29784	Viruses; Duplodnaviria; Heunggongvirae; Uroviricota; Caudoviricetes; Caudovirales; Siphoviridae; Pahexavirus; unclassified Pahexavirus
106	MG820639	Propionibacterium phage pa9-6919-4, complete genome	29784	Viruses; Duplodnaviria; Heunggongvirae; Uroviricota; Caudoviricetes; Caudovirales; Siphoviridae; Pahexavirus; unclassified Pahexavirus
107	MG820640	Propionibacterium phage pa15, complete genome	29309	Viruses; Duplodnaviria; Heunggongvirae; Uroviricota; Caudoviricetes; Caudovirales; Siphoviridae; Pahexavirus; unclassified Pahexavirus

108	MG820641	Propionibacterium phage pa615, complete genome	29307	Viruses; Duplodnaviria; Heunggongvirae; Uroviricota; Caudoviricetes; Caudovirales; Siphoviridae; Pahexavirus; unclassified Pahexavirus
109	MG820642	Propionibacterium phage pa28, complete genome	29733	Viruses; Duplodnaviria; Heunggongvirae; Uroviricota; Caudoviricetes; Caudovirales; Siphoviridae; Pahexavirus; unclassified Pahexavirus
110	MG820643	Propionibacterium phage pa35, complete genome	29491	Viruses; Duplodnaviria; Heunggongvirae; Uroviricota; Caudoviricetes; Caudovirales; Siphoviridae; Pahexavirus; unclassified Pahexavirus
111	MG820644	Propionibacterium phage pa33, complete genome	29135	Viruses; Duplodnaviria; Heunggongvirae; Uroviricota; Caudoviricetes; Caudovirales; Siphoviridae; Pahexavirus; unclassified Pahexavirus
112	MG820645	Propionibacterium phage pa3-SS3, complete genome	29137	Viruses; Duplodnaviria; Heunggongvirae; Uroviricota; Caudoviricetes; Caudovirales; Siphoviridae; Pahexavirus; unclassified Pahexavirus
113	MN329678	Propionibacterium phage Cota, complete genome	29549	Viruses; Duplodnaviria; Heunggongvirae; Uroviricota; Caudoviricetes; Caudovirales; Siphoviridae; Pahexavirus; unclassified Pahexavirus
114	MN813675	Cutibacterium phage P104B, complete genome	29330	Viruses; Duplodnaviria; Heunggongvirae; Uroviricota; Caudoviricetes; Caudovirales; Siphoviridae; Pahexavirus; unclassified Pahexavirus
115	MN813677	Cutibacterium phage P107C, complete genome	29516	Viruses; Duplodnaviria; Heunggongvirae; Uroviricota; Caudoviricetes; Caudovirales; Siphoviridae; Pahexavirus; unclassified Pahexavirus
116	MN813679	Cutibacterium phage P107A, complete genome	29465	Viruses; Duplodnaviria; Heunggongvirae; Uroviricota; Caudoviricetes; Caudovirales; Siphoviridae; Pahexavirus; unclassified Pahexavirus
117	MN813683	Cutibacterium phage P108C, complete genome	29494	Viruses; Duplodnaviria; Heunggongvirae; Uroviricota; Caudoviricetes; Caudovirales; Siphoviridae; Pahexavirus; unclassified Pahexavirus
118	MN813688	Cutibacterium phage P106I, complete genome	29470	Viruses; Duplodnaviria; Heunggongvirae; Uroviricota; Caudoviricetes; Caudovirales; Siphoviridae; Pahexavirus; unclassified Pahexavirus
119	MN813689	Cutibacterium phage P106L, complete genome	29641	Viruses; Duplodnaviria; Heunggongvirae; Uroviricota; Caudoviricetes; Caudovirales; Siphoviridae; Pahexavirus; unclassified Pahexavirus
120	MN813690	Cutibacterium phage P106M, complete genome	29641	Viruses; Duplodnaviria; Heunggongvirae; Uroviricota; Caudoviricetes; Caudovirales; Siphoviridae; Pahexavirus; unclassified Pahexavirus
121	MN813691	Cutibacterium phage P106A, complete genome	29554	Viruses; Duplodnaviria; Heunggongvirae; Uroviricota; Caudoviricetes; Caudovirales; Siphoviridae; Pahexavirus; unclassified Pahexavirus
122	MN813692	Cutibacterium phage P106C, complete genome	29641	Viruses; Duplodnaviria; Heunggongvirae; Uroviricota; Caudoviricetes; Caudovirales; Siphoviridae; Pahexavirus; unclassified Pahexavirus
123	MT647607	Propionibacterium phage phiPA50S, complete genome	29590	Viruses; Duplodnaviria; Heunggongvirae; Uroviricota; Caudoviricetes; Caudovirales; Siphoviridae; Pahexavirus; unclassified Pahexavirus
124	MW161461	Cutibacterium phage FD1, complete genome	29774	Viruses; Duplodnaviria; Heunggongvirae; Uroviricota; Caudoviricetes; Caudovirales; Siphoviridae; Pahexavirus; unclassified Pahexavirus
125	MW161462	Cutibacterium phage FD2, complete genome	29768	Viruses; Duplodnaviria; Heunggongvirae; Uroviricota; Caudoviricetes; Caudovirales; Siphoviridae; Pahexavirus; unclassified Pahexavirus
126	MW161463	Cutibacterium phage FD3, complete genome	29638	Viruses; Duplodnaviria; Heunggongvirae; Uroviricota; Caudoviricetes; Caudovirales; Siphoviridae; Pahexavirus; unclassified Pahexavirus
127	MW161464	Cutibacterium phage PAVL20, complete genome	29800	Viruses; Duplodnaviria; Heunggongvirae; Uroviricota; Caudoviricetes; Caudovirales; Siphoviridae; Pahexavirus; unclassified Pahexavirus
128	MW161465	Cutibacterium phage PAVL21, complete genome	30034	Viruses; Duplodnaviria; Heunggongvirae; Uroviricota; Caudoviricetes; Caudovirales; Siphoviridae; Pahexavirus; unclassified Pahexavirus
129	MW161466	Cutibacterium phage PAVL33, complete genome	29627	Viruses; Duplodnaviria; Heunggongvirae; Uroviricota; Caudoviricetes; Caudovirales; Siphoviridae; Pahexavirus; unclassified Pahexavirus

130	MW161467	Cutibacterium phage PAVL34, complete genome	29535	Viruses; Duplodnaviria; Heunggongvirae; Uroviricota; Caudoviricetes; Caudovirales; Siphoviridae; Pahexavirus; unclassified Pahexavirus
131	MW161468	Cutibacterium phage PAVL45, complete genome	29772	Viruses; Duplodnaviria; Heunggongvirae; Uroviricota; Caudoviricetes; Caudovirales; Siphoviridae; Pahexavirus; unclassified Pahexavirus
132	NC_009541	Propionibacterium phage PA6, complete genome	29739	Viruses; Duplodnaviria; Heunggongvirae; Uroviricota; Caudoviricetes; Caudovirales; Siphoviridae; Pahexavirus
133	NC_015453	Propionibacterium phage PAS50 endogenous virus, compl	29017	Viruses; Duplodnaviria; Heunggongvirae; Uroviricota; Caudoviricetes; Caudovirales; Siphoviridae; Pahexavirus
134	NC_015454	Propionibacterium phage PAD20 endogenous virus, compl	29074	Viruses; Duplodnaviria; Heunggongvirae; Uroviricota; Caudoviricetes; Caudovirales; Siphoviridae; Pahexavirus
135	NC_018834	Propionibacterium phage P9.1, complete genome	29214	Viruses; Duplodnaviria; Heunggongvirae; Uroviricota; Caudoviricetes; Caudovirales; Siphoviridae; Pahexavirus
136	NC_018838	Propionibacterium phage P100_A, complete genome	29505	Viruses; Duplodnaviria; Heunggongvirae; Uroviricota; Caudoviricetes; Caudovirales; Siphoviridae; Pahexavirus
137	NC_018839	Propionibacterium phage P14.4, complete genome	29729	Viruses; Duplodnaviria; Heunggongvirae; Uroviricota; Caudoviricetes; Caudovirales; Siphoviridae; Pahexavirus
138	NC_018840	Propionibacterium phage P100_1, complete genome	29612	Viruses; Duplodnaviria; Heunggongvirae; Uroviricota; Caudoviricetes; Caudovirales; Siphoviridae; Pahexavirus
139	NC_018841	Propionibacterium phage P101A, complete genome	29574	Viruses; Duplodnaviria; Heunggongvirae; Uroviricota; Caudoviricetes; Caudovirales; Siphoviridae; Pahexavirus
140	NC_018842	Propionibacterium phage P1.1, complete genome	29348	Viruses; Duplodnaviria; Heunggongvirae; Uroviricota; Caudoviricetes; Caudovirales; Siphoviridae; Pahexavirus
141	NC_018845	Propionibacterium phage P104A, complete genome	29371	Viruses; Duplodnaviria; Heunggongvirae; Uroviricota; Caudoviricetes; Caudovirales; Siphoviridae; Pahexavirus
142	NC_018847	Propionibacterium phage ATCC29399B_T, complete geno	29516	Viruses; Duplodnaviria; Heunggongvirae; Uroviricota; Caudoviricetes; Caudovirales; Siphoviridae; Pahexavirus
143	NC_018849	Propionibacterium phage P105, complete genome	29202	Viruses; Duplodnaviria; Heunggongvirae; Uroviricota; Caudoviricetes; Caudovirales; Siphoviridae; Pahexavirus
144	NC_018851	Propionibacterium phage ATCC29399B_C, complete geno	29516	Viruses; Duplodnaviria; Heunggongvirae; Uroviricota; Caudoviricetes; Caudovirales; Siphoviridae; Pahexavirus
145	NC_018852	Propionibacterium phage P100D, complete genome	29506	Viruses; Duplodnaviria; Heunggongvirae; Uroviricota; Caudoviricetes; Caudovirales; Siphoviridae; Pahexavirus
146	NC_022334	Propionibacterium phage PHL112N00, complete genome	29266	Viruses; Duplodnaviria; Heunggongvirae; Uroviricota; Caudoviricetes; Caudovirales; Siphoviridae; Pahexavirus
147	NC_022335	Propionibacterium phage PHL067M10, complete genome	29377	Viruses; Duplodnaviria; Heunggongvirae; Uroviricota; Caudoviricetes; Caudovirales; Siphoviridae; Pahexavirus; unclassified Pahexavirus
148	NC_022336	Propionibacterium phage PHL010M04, complete genome	29511	Viruses; Duplodnaviria; Heunggongvirae; Uroviricota; Caudoviricetes; Caudovirales; Siphoviridae; Pahexavirus; unclassified Pahexavirus
149	NC_022337	Propionibacterium phage PHL071N05, complete genome	29467	Viruses; Duplodnaviria; Heunggongvirae; Uroviricota; Caudoviricetes; Caudovirales; Siphoviridae; Pahexavirus

150	NC_022338	Propionibacterium phage PHL060L00, complete genome	29514	Viruses; Duplodnaviria; Heunggongvirae; Uroviricota; Caudoviricetes; Caudovirales; Siphoviridae; Pahexavirus
151	NC_022339	Propionibacterium phage PHL037M02, complete genome	29443	Viruses; Duplodnaviria; Heunggongvirae; Uroviricota; Caudoviricetes; Caudovirales; Siphoviridae; Pahexavirus
152	NC_022340	Propionibacterium phage PHL114L00, complete genome	29464	Viruses; Duplodnaviria; Heunggongvirae; Uroviricota; Caudoviricetes; Caudovirales; Siphoviridae; Pahexavirus
153	NC_022341	Propionibacterium phage PHL113M01, complete genome	29200	Viruses; Duplodnaviria; Heunggongvirae; Uroviricota; Caudoviricetes; Caudovirales; Siphoviridae; Pahexavirus
154	NC_022342	Propionibacterium phage PHL111M01, complete genome	29140	Viruses; Duplodnaviria; Heunggongvirae; Uroviricota; Caudoviricetes; Caudovirales; Siphoviridae; Pahexavirus
155	NC_027294	Propionibacterium phage PHL150M00, complete genome	29438	Viruses; Duplodnaviria; Heunggongvirae; Uroviricota; Caudoviricetes; Caudovirales; Siphoviridae; Pahexavirus; unclassified Pahexavirus
156	NC_027295	Propionibacterium phage PHL199M00, complete genome	29806	Viruses; Duplodnaviria; Heunggongvirae; Uroviricota; Caudoviricetes; Caudovirales; Siphoviridae; Pahexavirus
157	NC_027333	Propionibacterium phage PHL070N00, complete genome	29421	Viruses; Duplodnaviria; Heunggongvirae; Uroviricota; Caudoviricetes; Caudovirales; Siphoviridae; Pahexavirus
158	NC_027336	Propionibacterium phage PHL009M11, complete genome	29503	Viruses; Duplodnaviria; Heunggongvirae; Uroviricota; Caudoviricetes; Caudovirales; Siphoviridae; Pahexavirus
159	NC_027346	Propionibacterium phage PHL171M01, complete genome	29327	Viruses; Duplodnaviria; Heunggongvirae; Uroviricota; Caudoviricetes; Caudovirales; Siphoviridae; Pahexavirus
160	NC_027347	Propionibacterium phage PHL151M00, complete genome	29511	Viruses; Duplodnaviria; Heunggongvirae; Uroviricota; Caudoviricetes; Caudovirales; Siphoviridae; Pahexavirus
161	NC_027354	Propionibacterium phage PHL301M00, complete genome	29323	Viruses; Duplodnaviria; Heunggongvirae; Uroviricota; Caudoviricetes; Caudovirales; Siphoviridae; Pahexavirus
162	NC_027357	Propionibacterium phage PHL025M00, complete genome	29496	Viruses; Duplodnaviria; Heunggongvirae; Uroviricota; Caudoviricetes; Caudovirales; Siphoviridae; Pahexavirus
163	NC_027359	Propionibacterium phage PHL082M00, complete genome	29491	Viruses; Duplodnaviria; Heunggongvirae; Uroviricota; Caudoviricetes; Caudovirales; Siphoviridae; Pahexavirus; unclassified Pahexavirus
164	NC_027361	Propionibacterium phage PHL085N00, complete genome	29454	Viruses; Duplodnaviria; Heunggongvirae; Uroviricota; Caudoviricetes; Caudovirales; Siphoviridae; Pahexavirus; unclassified Pahexavirus
165	NC_027362	Propionibacterium phage PHL116M00, complete genome	29394	Viruses; Duplodnaviria; Heunggongvirae; Uroviricota; Caudoviricetes; Caudovirales; Siphoviridae; Pahexavirus
166	NC_027367	Propionibacterium phage PHL132N00, complete genome	29003	Viruses; Duplodnaviria; Heunggongvirae; Uroviricota; Caudoviricetes; Caudovirales; Siphoviridae; Pahexavirus
167	NC_027370	Propionibacterium phage PHL179M00, complete genome	29428	Viruses; Duplodnaviria; Heunggongvirae; Uroviricota; Caudoviricetes; Caudovirales; Siphoviridae; Pahexavirus
168	NC_027371	Propionibacterium phage Pacnes 2012-15, complete geno	29741	Viruses; Duplodnaviria; Heunggongvirae; Uroviricota; Caudoviricetes; Caudovirales; Siphoviridae; Pahexavirus
169	NC_027373	Propionibacterium phage PHL030N00, complete genome	29423	Viruses; Duplodnaviria; Heunggongvirae; Uroviricota; Caudoviricetes; Caudovirales; Siphoviridae; Pahexavirus; unclassified Pahexavirus
170	NC_027376	Propionibacterium phage PHL308M00, complete genome	29442	Viruses; Duplodnaviria; Heunggongvirae; Uroviricota; Caudoviricetes; Caudovirales; Siphoviridae; Pahexavirus
171	NC_027380	Propionibacterium phage PHL067M01, complete genome	29377	Viruses; Duplodnaviria; Heunggongvirae; Uroviricota; Caudoviricetes; Caudovirales; Siphoviridae; Pahexavirus

172	NC_027385	Propionibacterium phage PHL092M00, complete genome	29261	Viruses; Duplodnaviria; Heunggongvirae; Uroviricota; Caudoviricetes; Caudovirales; Siphoviridae; Pahexavirus
173	NC_027386	Propionibacterium phage PHL152M00, complete genome	29247	Viruses; Duplodnaviria; Heunggongvirae; Uroviricota; Caudoviricetes; Caudovirales; Siphoviridae; Pahexavirus
174	NC_027389	Propionibacterium phage PHL141N00, complete genome	29494	Viruses; Duplodnaviria; Heunggongvirae; Uroviricota; Caudoviricetes; Caudovirales; Siphoviridae; Pahexavirus
175	NC_027391	Propionibacterium phage PHL041M10, complete genome	29412	Viruses; Duplodnaviria; Heunggongvirae; Uroviricota; Caudoviricetes; Caudovirales; Siphoviridae; Pahexavirus
176	NC_027392	Propionibacterium phage PHL194M00, complete genome	29264	Viruses; Duplodnaviria; Heunggongvirae; Uroviricota; Caudoviricetes; Caudovirales; Siphoviridae; Pahexavirus
177	NC_027400	Propionibacterium phage PHL055N00, complete genome	29264	Viruses; Duplodnaviria; Heunggongvirae; Uroviricota; Caudoviricetes; Caudovirales; Siphoviridae; Pahexavirus; unclassified Pahexavirus
178	NC_027401	Propionibacterium phage PHL095N00, complete genome	29751	Viruses; Duplodnaviria; Heunggongvirae; Uroviricota; Caudoviricetes; Caudovirales; Siphoviridae; Pahexavirus
179	NC_027403	Propionibacterium phage PHL117M00, complete genome	29255	Viruses; Duplodnaviria; Heunggongvirae; Uroviricota; Caudoviricetes; Caudovirales; Siphoviridae; Pahexavirus
180	NC_027405	Propionibacterium phage PHL163M00, complete genome	29264	Viruses; Duplodnaviria; Heunggongvirae; Uroviricota; Caudoviricetes; Caudovirales; Siphoviridae; Pahexavirus
181	NC_027620	Propionibacterium phage MrAK, complete genome	29726	Viruses; Duplodnaviria; Heunggongvirae; Uroviricota; Caudoviricetes; Caudovirales; Siphoviridae; Pahexavirus
182	NC_027621	Propionibacterium phage Wizzo, complete genome	29463	Viruses; Duplodnaviria; Heunggongvirae; Uroviricota; Caudoviricetes; Caudovirales; Siphoviridae; Pahexavirus
183	NC_027622	Propionibacterium phage Stormborn, complete genome	29330	Viruses; Duplodnaviria; Heunggongvirae; Uroviricota; Caudoviricetes; Caudovirales; Siphoviridae; Pahexavirus
184	NC_027623	Propionibacterium phage Pirate, complete genome	29324	Viruses; Duplodnaviria; Heunggongvirae; Uroviricota; Caudoviricetes; Caudovirales; Siphoviridae; Pahexavirus
185	NC_027624	Propionibacterium phage SKKY, complete genome	29594	Viruses; Duplodnaviria; Heunggongvirae; Uroviricota; Caudoviricetes; Caudovirales; Siphoviridae; Pahexavirus
186	NC_027625	Propionibacterium phage Kubed, complete genome	29461	Viruses; Duplodnaviria; Heunggongvirae; Uroviricota; Caudoviricetes; Caudovirales; Siphoviridae; Pahexavirus
187	NC_027626	Propionibacterium phage Procrass1, complete genome	29347	Viruses; Duplodnaviria; Heunggongvirae; Uroviricota; Caudoviricetes; Caudovirales; Siphoviridae; Pahexavirus
188	NC_027627	Propionibacterium phage Solid, complete genome	29440	Viruses; Duplodnaviria; Heunggongvirae; Uroviricota; Caudoviricetes; Caudovirales; Siphoviridae; Pahexavirus
189	NC_027628	Propionibacterium phage Lauchelly, complete genome	29517	Viruses; Duplodnaviria; Heunggongvirae; Uroviricota; Caudoviricetes; Caudovirales; Siphoviridae; Pahexavirus
190	NC_027629	Propionibacterium phage Attacne, complete genome	28876	Viruses; Duplodnaviria; Heunggongvirae; Uroviricota; Caudoviricetes; Caudovirales; Siphoviridae; Pahexavirus
191	NC_027630	Propionibacterium phage Ouroboros, complete genome	29506	Viruses; Duplodnaviria; Heunggongvirae; Uroviricota; Caudoviricetes; Caudovirales; Siphoviridae; Pahexavirus
192	NC_028694	Propionibacterium phage PA1-14, complete genome	29407	Viruses; Duplodnaviria; Heunggongvirae; Uroviricota; Caudoviricetes; Caudovirales; Siphoviridae; Pahexavirus; unclassified Pahexavirus
193	NC_028967	Propionibacterium phage PAC1, complete genome	29605	Viruses; Duplodnaviria; Heunggongvirae; Uroviricota; Caudoviricetes; Caudovirales; Siphoviridae; Pahexavirus; unclassified Pahexavirus

194	NC_031003	Propionibacterium phage Moyashi, complete genome	29254	Viruses; Duplodnaviria; Heunggongvirae; Uroviricota; Caudoviricetes; Caudovirales; Siphoviridae; Pahexavirus; unclassified Pahexavirus
195	NC_031005	Propionibacterium phage QueenBey, complete genome	29338	Viruses; Duplodnaviria; Heunggongvirae; Uroviricota; Caudoviricetes; Caudovirales; Siphoviridae; Pahexavirus; unclassified Pahexavirus
196	NC_031084	Propionibacterium phage BruceLethal, complete genome	29249	Viruses; Duplodnaviria; Heunggongvirae; Uroviricota; Caudoviricetes; Caudovirales; Siphoviridae; Pahexavirus; unclassified Pahexavirus
197	NC_031119	Propionibacterium phage Enoki, complete genome	29347	Viruses; Duplodnaviria; Heunggongvirae; Uroviricota; Caudoviricetes; Caudovirales; Siphoviridae; Pahexavirus; unclassified Pahexavirus
198	NC_041954	Propionibacterium phage Keiki, complete genome	29339	Viruses; Duplodnaviria; Heunggongvirae; Uroviricota; Caudoviricetes; Caudovirales; Siphoviridae; Pahexavirus
199	NC_041955	Propionibacterium phage PHL082M03, complete genome	29491	Viruses; Duplodnaviria; Heunggongvirae; Uroviricota; Caudoviricetes; Caudovirales; Siphoviridae; Pahexavirus
200	NC_041956	Propionibacterium phage PHL117M01, complete genome	29422	Viruses; Duplodnaviria; Heunggongvirae; Uroviricota; Caudoviricetes; Caudovirales; Siphoviridae; Pahexavirus
201	NC_041957	Propionibacterium phage PHL151N00, complete genome	29511	Viruses; Duplodnaviria; Heunggongvirae; Uroviricota; Caudoviricetes; Caudovirales; Siphoviridae; Pahexavirus

Table S2.

Annotation of the CDSs of the eight phages isolated in this work. The annotations were performed using RAST version 2 (https://rast.nmpdr.org/rast.cgi, accessed on March 1, 2021), Phage Search Tool Enhanced Release (PHASTER) (https: //phaster.ca, accessed on March 1, 2021), and the BLAST server.

Table S2.

Index	Sequence Name	Protein id	Start	End	Length	Direction	Annotation
1	FD1	QPB11540.1	22042	23553	1512	forward	hypothetical protein CDS
2	FD1	QPB11541.1	23550	24875	1326	forward	hypothetical protein CDS
3	FD1	QPB11506.1	4134	5297	1164	forward	hypothetical protein CDS
4	FD1	QPB11517.1	10181	11218	1038	reverse	hypothetical protein CDS
5	FD1	QPB11505.1	3186	4133	948	forward	hypothetical protein CDS
6	FD1	QPB11527.1	15602	16549	948	reverse	exonuclease CDS
7	FD1	QPB11545.1	26311	27258	948	forward	hypothetical protein CDS
8	FD1	QPB11507.1	5317	6135	819	forward	hypothetical protein CDS
9	FD1	QPB11509.1	6443	7249	807	forward	hypothetical protein CDS
10	FD1	QPB11542.1	24882	25637	756	forward	hypothetical protein CDS
11	FD1	QPB11523.1	13782	14522	741	reverse	hypothetical protein CDS
12	FD1	QPB11521.1	12557	13228	672	reverse	DNA primase CDS
13	FD1	QPB11510.1	7495	8148	654	forward	hypothetical protein CDS
14	FD1	QPB11501.1	1280	1900	621	reverse	hypothetical protein CDS
15	FD1	QPB11550.1	28832	29431	600	forward	hypothetical protein CDS
16	FD1	QPB11520.1	11950	12513	564	reverse	hypothetical protein CDS
17	FD1	QPB11519.1	11393	11953	561	reverse	hypothetical protein CDS
18	FD1	QPB11531.1	17466	17999	534	reverse	hypothetical protein CDS
19	FD1	QPB11513.1	8647	9168	522	reverse	hypothetical protein CDS
20	FD1	QPB11546.1	27302	27766	465	forward	hypothetical protein CDS
21	FD1	QPB11504.1	2709	3170	462	forward	tail length tape-measure protein CDS
22	FD1	QPB11544.1	25876	26304	429	forward	hypothetical protein CDS
23	FD1	QPB11532.1	18230	18646	417	forward	hypothetical protein CDS
24	FD1	QPB11526.1	15195	15605	411	reverse	hypothetical protein CDS
25	FD1	QPB11514.1	9173	9565	393	reverse	hypothetical protein CDS
26	FD1	QPB11539.1	21666	22037	372	forward	hypothetical protein CDS
27	FD1	QPB11549.1	28409	28780	372	forward	hypothetical protein CDS
28	FD1	QPB11528.1	16549	16908	360	reverse	hypothetical protein CDS
29	FD1	QPB11522.1	13426	13782	357	reverse	hypothetical protein CDS
30	FD1	QPB11547.1	27768	28115	348	forward	hypothetical protein CDS
31	FD1	QPB11516.1	9853	10173	321	reverse	hypothetical protein CDS
32	FD1	QPB11499.1	387	692	306	reverse	hypothetical protein CDS
33	FD1	QPB11502.1	1952	2251	300	reverse	hypothetical protein CDS
34	FD1	QPB11548.1	28122	28412	291	forward	hypothetical protein CDS
35	FD1	QPB11503.1	2432	2710	279	reverse	hypothetical protein CDS
36	FD1	QPB11515.1	9569	9835	267	reverse	hypothetical protein CDS
37	FD1	QPB11508.1	6177	6440	264	forward	hypothetical protein CDS
38	FD1	QPB11534.1	18871	19119	249	forward	hypothetical protein CDS
39	FD1	QPB11535.1	19270	19503	234	reverse	hypothetical protein CDS
40	FD1	QPB11524.1	14580	14807	228	forward	hypothetical protein CDS
41	FD1	QPB11530.1	17215	17442	228	reverse	hypothetical protein CDS

42	FD1	QPB11533.1	18650	18871	222	forward	hypothetical protein CDS
43	FD1	QPB11500.1	878	1081	204	reverse	hypothetical protein CDS
44	FD1	QPB11529.1	17015	17218	204	reverse	hypothetical protein CDS
45	FD1	QPB11518.1	11215	11409	195	reverse	hypothetical protein CDS
46	FD1	QPB11525.1	14819	15013	195	forward	hypothetical protein CDS
47	FD1	QPB11536.1	19738	19932	195	reverse	hypothetical protein CDS
48	FD1	QPB11543.1	25669	25860	192	reverse	hypothetical protein CDS
49	FD1	QPB11537.1	21248	21427	180	reverse	hypothetical protein CDS
50	FD1	QPB11498.1	224	397	174	forward	hypothetical protein CDS
51	FD1	QPB11538.1	21431	21604	174	reverse	hypothetical protein CDS
52	FD1	QPB11511.1	8214	8384	171	reverse	hypothetical protein CDS
53	FD1	QPB11512.1	8440	8562	123	forward	putative holin CDS
54	FD2	QPB11592.1	22036	23547	1512	forward	hypothetical protein CDS
55	FD2	QPB11593.1	23544	24869	1326	forward	hypothetical protein CDS
56	FD2	QPB11559.1	4134	5297	1164	forward	hypothetical protein CDS
57	FD2	QPB11569.1	10181	11218	1038	reverse	hypothetical protein CDS
58	FD2	QPB11558.1	3186	4133	948	forward	hypothetical protein CDS
59	FD2	QPB11578.1	15602	16549	948	reverse	exonuclease CDS
60	FD2	QPB11596.1	26305	27234	930	forward	hypothetical protein CDS
61	FD2	QPB11575.1	13782	14645	864	reverse	hypothetical protein CDS
62	FD2	QPB11563.1	7291	8148	858	forward	hypothetical protein CDS
63	FD2	QPB11560.1	5317	6135	819	forward	hypothetical protein CDS
64	FD2	QPB11562.1	6443	7249	807	forward	hypothetical protein CDS
65	FD2	QPB11594.1	24876	25631	756	forward	hypothetical protein CDS
66	FD2	QPB11573.1	12557	13228	672	reverse	DNA primase CDS
67	FD2	QPB11601.1	28827	29456	630	forward	hypothetical protein CDS
68	FD2	QPB11554.1	1280	1900	621	reverse	hypothetical protein CDS
69	FD2	QPB11572.1	11950	12513	564	reverse	hypothetical protein CDS
70	FD2	QPB11571.1	11393	11953	561	reverse	hypothetical protein CDS
71	FD2	QPB11595.1	25741	26298	558	forward	hypothetical protein CDS
72	FD2	QPB11582.1	17466	17999	534	reverse	hypothetical protein CDS
73	FD2	QPB11565.1	8647	9168	522	reverse	hypothetical protein CDS
74	FD2	QPB11576.1	14686	15156	471	reverse	hypothetical protein CDS
75	FD2	QPB11557.1	2709	3170	462	forward	tail length tape-measure protein CDS
76	FD2	QPB11597.1	27300	27761	462	forward	hypothetical protein CDS
77	FD2	QPB11591.1	21594	22031	438	forward	hypothetical protein CDS
78	FD2	QPB11583.1	18230	18646	417	forward	hypothetical protein CDS
79	FD2	QPB11577.1	15195	15605	411	reverse	hypothetical protein CDS
80	FD2	QPB11564.1	8161	8562	402	forward	putative holin CDS
81	FD2	QPB11566.1	9173	9565	393	reverse	hypothetical protein CDS
82	FD2	QPB11600.1	28404	28775	372	forward	hypothetical protein CDS
83	FD2	QPB11579.1	16549	16908	360	reverse	hypothetical protein CDS
84	FD2	QPB11574.1	13426	13782	357	reverse	hypothetical protein CDS
85	FD2	QPB11598.1	27763	28110	348	forward	hypothetical protein CDS
86	FD2	QPB11568.1	9853	10173	321	reverse	hypothetical protein CDS
87	FD2	QPB11552.1	387	692	306	reverse	hypothetical protein CDS
88	FD2	QPB11555.1	1952	2242	291	reverse	hypothetical protein CDS
89	FD2	QPB11599.1	28117	28407	291	forward	hypothetical protein CDS
90	FD2	QPB11556.1	2432	2710	279	reverse	hypothetical protein CDS
91	FD2	QPB11567.1	9569	9835	267	reverse	hypothetical protein CDS
92	FD2	QPB11561.1	6177	6440	264	forward	hypothetical protein CDS
93	FD2	QPB11585.1	18871	19119	249	forward	hypothetical protein CDS
94	FD2	QPB11586.1	19270	19503	234	reverse	hypothetical protein CDS

95	FD2	QPB11581.1	17215	17442	228	reverse	hypothetical protein CDS
96	FD2	QPB11584.1	18650	18871	222	forward	hypothetical protein CDS
97	FD2	QPB11553.1	878	1081	204	reverse	hypothetical protein CDS
98	FD2	QPB11580.1	17015	17218	204	reverse	hypothetical protein CDS
99	FD2	QPB11588.1	20952	21152	201	reverse	hypothetical protein CDS
100	FD2	QPB11570.1	11215	11409	195	reverse	hypothetical protein CDS
101	FD2	QPB11587.1	19738	19932	195	reverse	hypothetical protein CDS
102	FD2	QPB11589.1	21247	21426	180	reverse	hypothetical protein CDS
103	FD2	QPB11551.1	224	397	174	forward	hypothetical protein CDS
104	FD2	QPB11590.1	21430	21573	144	reverse	hypothetical protein CDS
105	FD3	QPB11641.1	21906	23417	1512	forward	hypothetical protein CDS
106	FD3	QPB11642.1	23414	24739	1326	forward	hypothetical protein CDS
107	FD3	QPB11610.1	4133	5293	1161	forward	hypothetical protein CDS
108	FD3	QPB11626.1	13597	14664	1068	reverse	hypothetical protein CDS
109	FD3	QPB11620.1	10185	11231	1047	reverse	hypothetical protein CDS
110	FD3	QPB11609.1	3151	4128	978	forward	hypothetical protein CDS
111	FD3	QPB11646.1	26162	27112	951	forward	hypothetical protein CDS
112	FD3	QPB11629.1	15622	16563	942	reverse	exonuclease CDS
113	FD3	QPB11611.1	5313	6131	819	forward	hypothetical protein CDS
114	FD3	QPB11614.1	7347	8153	807	forward	hypothetical protein CDS
115	FD3	QPB11613.1	6446	7243	798	forward	hypothetical protein CDS
116	FD3	QPB11643.1	24746	25501	756	forward	hypothetical protein CDS
117	FD3	QPB11624.1	12576	13247	672	reverse	DNA primase CDS
118	FD3	QPB11651.1	28683	29324	642	forward	major tail protein CDS
119	FD3	QPB11623.1	11966	12529	564	reverse	hypothetical protein CDS
120	FD3	QPB11622.1	11409	11969	561	reverse	hypothetical protein CDS
121	FD3	QPB11645.1	25604	26158	555	forward	hypothetical protein CDS
122	FD3	QPB11633.1	17483	18022	540	reverse	hypothetical protein CDS
123	FD3	QPB11616.1	8647	9168	522	reverse	hypothetical protein CDS
124	FD3	QPB11607.1	2321	2809	489	reverse	hypothetical protein CDS
125	FD3	QPB11647.1	27129	27617	489	forward	hypothetical protein CDS
126	FD3	QPB11627.1	14705	15169	465	reverse	hypothetical protein CDS
127	FD3	QPB11615.1	8157	8561	405	forward	putative holin CDS
128	FD3	QPB11628.1	15221	15625	405	reverse	hypothetical protein CDS
129	FD3	QPB11617.1	9174	9566	393	reverse	hypothetical protein CDS
130	FD3	QPB11648.1	27592	27966	375	forward	hypothetical protein CDS
131	FD3	QPB11650.1	28260	28631	372	forward	hypothetical protein CDS
132	FD3	QPB11608.1	2808	3170	363	forward	tail length tape-measure protein CDS
133	FD3	QPB11636.1	18729	19085	357	forward	hypothetical protein CDS
134	FD3	QPB11640.1	21553	21906	354	forward	hypothetical protein CDS
135	FD3	QPB11630.1	16563	16904	342	reverse	hypothetical protein CDS
136	FD3	QPB11619.1	9854	10174	321	reverse	hypothetical protein CDS
137	FD3	QPB11602.1	387	692	306	reverse	hypothetical protein CDS
138	FD3	QPB11639.1	21169	21471	303	forward	hypothetical protein CDS
139	FD3	QPB11649.1	27973	28263	291	forward	hypothetical protein CDS
140	FD3	QPB11635.1	18292	18579	288	forward	hypothetical protein CDS
141	FD3	QPB11612.1	6177	6443	267	forward	hypothetical protein CDS
142	FD3	QPB11618.1	9570	9836	267	reverse	hypothetical protein CDS
143	FD3	QPB11605.1	1721	1972	252	reverse	hypothetical protein CDS
144	FD3	QPB11606.1	2003	2251	249	reverse	hypothetical protein CDS
145	FD3	QPB11638.1	19651	19896	246	reverse	hypothetical protein CDS
146	FD3	QPB11632.1	17230	17460	231	reverse	hypothetical protein CDS
147	FD3	QPB11631.1	17028	17237	210	reverse	hypothetical protein CDS

148	FD3	QPB11621.1	11228	11425	198	reverse	hypothetical protein CDS
149	FD3	QPB11625.1	13282	13470	189	forward	hypothetical protein CDS
150	FD3	QPB11603.1	878	1054	177	reverse	hypothetical protein CDS
151	FD3	QPB11634.1	18132	18302	171	forward	hypothetical protein CDS
152	FD3	QPB11604.1	1280	1447	168	reverse	hypothetical protein CDS
153	FD3	QPB11644.1	25498	25623	126	forward	hypothetical protein CDS
154	FD3	QPB11637.1	19100	19201	102	forward	hypothetical protein CDS
155	PAVL20	QPB11653.1	405	3170	2766	forward	hypothetical protein CDS
156	PAVL20	QPB11688.1	22068	23579	1512	forward	hypothetical protein CDS
157	PAVL20	QPB11689.1	23576	24901	1326	forward	hypothetical protein CDS
158	PAVL20	QPB11655.1	4134	5297	1164	forward	hypothetical protein CDS
159	PAVL20	QPB11665.1	10181	11218	1038	reverse	hypothetical protein CDS
160	PAVL20	QPB11654.1	3186	4133	948	forward	hypothetical protein CDS
161	PAVL20	QPB11675.1	15602	16549	948	reverse	exonuclease CDS
162	PAVL20	QPB11693.1	26337	27284	948	forward	hypothetical protein CDS
163	PAVL20	QPB11659.1	7291	8148	858	forward	hypothetical protein CDS
164	PAVL20	QPB11656.1	5317	6135	819	forward	hypothetical protein CDS
165	PAVL20	QPB11658.1	6443	7249	807	forward	hypothetical protein CDS
166	PAVL20	QPB11690.1	24908	25663	756	forward	hypothetical protein CDS
167	PAVL20	QPB11671.1	13782	14522	741	reverse	hypothetical protein CDS
168	PAVL20	QPB11669.1	12557	13228	672	reverse	DNA primase CDS
169	PAVL20	QPB11698.1	28858	29457	600	forward	hypothetical protein CDS
170	PAVL20	QPB11668.1	11950	12513	564	reverse	hypothetical protein CDS
171	PAVL20	QPB11667.1	11393	11953	561	reverse	hypothetical protein CDS
172	PAVL20	QPB11679.1	17466	17999	534	reverse	hypothetical protein CDS
173	PAVL20	QPB11661.1	8647	9168	522	reverse	hypothetical protein CDS
174	PAVL20	QPB11692.1	25854	26330	477	forward	hypothetical protein CDS
175	PAVL20	QPB11694.1	27331	27792	462	forward	hypothetical protein CDS
176	PAVL20	QPB11680.1	18230	18646	417	forward	hypothetical protein CDS
177	PAVL20	QPB11674.1	15195	15605	411	reverse	hypothetical protein CDS
178	PAVL20	QPB11662.1	9173	9565	393	reverse	hypothetical protein CDS
179	PAVL20	QPB11687.1	21692	22063	372	forward	hypothetical protein CDS
180	PAVL20	QPB11697.1	28435	28806	372	forward	hypothetical protein CDS
181	PAVL20	QPB11676.1	16549	16908	360	reverse	hypothetical protein CDS
182	PAVL20	QPB11670.1	13426	13782	357	reverse	hypothetical protein CDS
183	PAVL20	QPB11695.1	27794	28141	348	forward	hypothetical protein CDS
184	PAVL20	QPB11664.1	9853	10173	321	reverse	hypothetical protein CDS
185	PAVL20	QPB11686.1	21295	21600	306	forward	hypothetical protein CDS
186	PAVL20	QPB11696.1	28148	28438	291	forward	hypothetical protein CDS
187	PAVL20	QPB11660.1	8281	8562	282	forward	putative holin CDS
188	PAVL20	QPB11663.1	9569	9835	267	reverse	hypothetical protein CDS
189	PAVL20	QPB11657.1	6177	6440	264	forward	hypothetical protein CDS
190	PAVL20	QPB11682.1	18868	19119	252	forward	hypothetical protein CDS
191	PAVL20	QPB11683.1	19270	19503	234	reverse	hypothetical protein CDS
192	PAVL20	QPB11672.1	14580	14807	228	forward	hypothetical protein CDS
193	PAVL20	QPB11678.1	17215	17442	228	reverse	hypothetical protein CDS
194	PAVL20	QPB11681.1	18650	18871	222	forward	hypothetical protein CDS
195	PAVL20	QPB11677.1	17015	17218	204	reverse	hypothetical protein CDS
196	PAVL20	QPB11685.1	20952	21152	201	reverse	hypothetical protein CDS
197	PAVL20	QPB11666.1	11215	11409	195	reverse	hypothetical protein CDS
198	PAVL20	QPB11673.1	14819	15013	195	forward	hypothetical protein CDS
199	PAVL20	QPB11684.1	19738	19932	195	reverse	hypothetical protein CDS
200	PAVL20	QPB11652.1	224	397	174	forward	hypothetical protein CDS

201	PAVL20	QPB11691.1	25670	25792	123	forward	hypothetical protein CDS
202	PAVL21	QPB11745.1	22297	23808	1512	forward	hypothetical protein CDS
203	PAVL21	QPB11746.1	23805	25130	1326	forward	hypothetical protein CDS
204	PAVL21	QPB11705.1	4137	5297	1161	forward	hypothetical protein CDS
205	PAVL21	QPB11717.1	10261	11307	1047	reverse	hypothetical protein CDS
206	PAVL21	QPB11703.1	2154	3170	1017	forward	tail length tape-measure protein CDS
207	PAVL21	QPB11704.1	3167	4132	966	forward	hypothetical protein CDS
208	PAVL21	QPB11750.1	26554	27501	948	forward	hypothetical protein CDS
209	PAVL21	QPB11706.1	5347	6135	789	forward	hypothetical protein CDS
210	PAVL21	QPB11747.1	25134	25892	759	forward	hypothetical protein CDS
211	PAVL21	QPB11702.1	1280	1972	693	reverse	hypothetical protein CDS
212	PAVL21	QPB11721.1	12649	13320	672	reverse	DNA primase CDS
213	PAVL21	QPB11755.1	29080	29721	642	forward	major tail protein CDS
214	PAVL21	QPB11720.1	12039	12605	567	reverse	hypothetical protein CDS
215	PAVL21	QPB11719.1	11482	12042	561	reverse	hypothetical protein CDS
216	PAVL21	QPB11711.1	7678	8232	555	forward	hypothetical protein CDS
217	PAVL21	QPB11735.1	17564	18097	534	reverse	hypothetical protein CDS
218	PAVL21	QPB11713.1	8733	9251	519	reverse	hypothetical protein CDS
219	PAVL21	QPB11708.1	6455	6931	477	forward	tail fiber protein CDS
220	PAVL21	QPB11749.1	26077	26550	474	forward	hypothetical protein CDS
221	PAVL21	QPB11752.1	27945	28355	411	forward	hypothetical protein CDS
222	PAVL21	QPB11712.1	8245	8646	402	forward	putative holin CDS
223	PAVL21	QPB11714.1	9256	9648	393	reverse	hypothetical protein CDS
224	PAVL21	QPB11728.1	15082	15474	393	forward	hypothetical protein CDS
225	PAVL21	QPB11731.1	16169	16561	393	forward	hypothetical protein CDS
226	PAVL21	QPB11709.1	6937	7320	384	forward	hypothetical protein CDS
227	PAVL21	QPB11744.1	21921	22292	372	forward	hypothetical protein CDS
228	PAVL21	QPB11754.1	28649	29020	372	forward	hypothetical protein CDS
229	PAVL21	QPB11722.1	13518	13874	357	reverse	hypothetical protein CDS
230	PAVL21	QPB11733.1	16958	17290	333	forward	hypothetical protein CDS
231	PAVL21	QPB11723.1	13886	14215	330	forward	hypothetical protein CDS
232	PAVL21	QPB11716.1	9930	10250	321	reverse	hypothetical protein CDS
233	PAVL21	QPB11736.1	18192	18512	321	forward	hypothetical protein CDS
234	PAVL21	QPB11739.1	18947	19255	309	reverse	hypothetical protein CDS
235	PAVL21	QPB11753.1	28362	28652	291	forward	hypothetical protein CDS
236	PAVL21	QPB11743.1	21584	21868	285	reverse	hypothetical protein CDS
237	PAVL21	QPB11715.1	9652	9918	267	reverse	hypothetical protein CDS
238	PAVL21	QPB11707.1	6180	6443	264	forward	hypothetical protein CDS
239	PAVL21	QPB11741.1	19969	20214	246	reverse	hypothetical protein CDS
240	PAVL21	QPB11742.1	21189	21431	243	reverse	hypothetical protein CDS
241	PAVL21	QPB11734.1	17319	17546	228	reverse	hypothetical protein CDS
242	PAVL21	QPB11727.1	14676	14900	225	forward	hypothetical protein CDS
243	PAVL21	QPB11701.1	878	1081	204	reverse	hypothetical protein CDS
244	PAVL21	QPB11748.1	25889	26080	192	forward	hypothetical protein CDS
245	PAVL21	QPB11729.1	15699	15887	189	forward	hypothetical protein CDS
246	PAVL21	QPB11700.1	244	429	186	reverse	hypothetical protein CDS
247	PAVL21	QPB11710.1	7343	7528	186	reverse	hypothetical protein CDS
248	PAVL21	QPB11732.1	16591	16776	186	forward	hypothetical protein CDS
249	PAVL21	QPB11738.1	18747	18929	183	forward	hypothetical protein CDS
250	PAVL21	QPB11730.1	15984	16148	165	forward	hypothetical protein CDS
251	PAVL21	QPB11737.1	18582	18743	162	forward	hypothetical protein CDS
252	PAVL21	QPB11699.1	88	234	147	reverse	hypothetical protein CDS
253	PAVL21	QPB11724.1	14228	14374	147	forward	hypothetical protein CDS

254	PAVL21	QPB11740.1	19481	19621	141	reverse	hypothetical protein CDS
255	PAVL21	QPB11751.1	27781	27900	120	reverse	hypothetical protein CDS
256	PAVL21	QPB11726.1	14541	14657	117	forward	hypothetical protein CDS
257	PAVL21	QPB11718.1	11368	11466	99	forward	hypothetical protein CDS
258	PAVL21	QPB11725.1	14445	14537	93	forward	hypothetical protein CDS
259	PAVL33	QPB11808.1	405	3170	2766	forward	hypothetical protein CDS
260	PAVL33	QPB11850.1	21822	23333	1512	forward	hypothetical protein CDS
261	PAVL33	QPB11851.1	23330	24649	1320	forward	capsid and scaffold protein CDS
262	PAVL33	QPB11810.1	4135	5292	1158	forward	hypothetical protein CDS
263	PAVL33	QPB11833.1	15291	16349	1059	reverse	exonuclease CDS
264	PAVL33	QPB11855.1	26050	27021	972	forward	major capsid protein CDS
265	PAVL33	QPB11809.1	3186	4127	942	forward	hypothetical protein CDS
266	PAVL33	QPB11813.1	7323	8186	864	forward	hypothetical protein CDS
267	PAVL33	QPB11811.1	5313	6131	819	forward	hypothetical protein CDS
268	PAVL33	QPB11812.1	6456	7271	816	forward	hypothetical protein CDS
269	PAVL33	QPB11852.1	24646	25410	765	forward	hypothetical protein CDS
270	PAVL33	QPB11826.1	12341	13012	672	reverse	DNA primase CDS
271	PAVL33	QPB11860.1	28592	29221	630	forward	hypothetical protein CDS
272	PAVL33	QPB11854.1	25519	26043	525	forward	hypothetical protein CDS
273	PAVL33	QPB11815.1	8690	9211	522	reverse	hypothetical protein CDS
274	PAVL33	QPB11856.1	27065	27526	462	forward	hypothetical protein CDS
275	PAVL33	QPB11818.1	10138	10584	447	forward	hypothetical protein CDS
276	PAVL33	QPB11814.1	8199	8603	405	forward	putative holin CDS
277	PAVL33	QPB11849.1	21416	21817	402	forward	hypothetical protein CDS
278	PAVL33	QPB11824.1	11707	12105	399	forward	hypothetical protein CDS
279	PAVL33	QPB11816.1	9217	9609	393	reverse	hypothetical protein CDS
280	PAVL33	QPB11859.1	28169	28540	372	forward	hypothetical protein CDS
281	PAVL33	QPB11834.1	16349	16708	360	reverse	hypothetical protein CDS
282	PAVL33	QPB11857.1	27528	27875	348	forward	hypothetical protein CDS
283	PAVL33	QPB11841.1	18295	18606	312	reverse	hypothetical protein CDS
284	PAVL33	QPB11832.1	14779	15087	309	forward	hypothetical protein CDS
285	PAVL33	QPB11842.1	18638	18946	309	reverse	hypothetical protein CDS
286	PAVL33	QPB11848.1	21069	21374	306	forward	hypothetical protein CDS
287	PAVL33	QPB11844.1	19534	19824	291	reverse	hypothetical protein CDS
288	PAVL33	QPB11858.1	27882	28172	291	forward	hypothetical protein CDS
289	PAVL33	QPB11807.1	110	397	288	forward	hypothetical protein CDS
290	PAVL33	QPB11817.1	9613	9897	285	reverse	hypothetical protein CDS
291	PAVL33	QPB11828.1	13578	13847	270	forward	hypothetical protein CDS
292	PAVL33	QPB11846.1	20438	20683	246	reverse	hypothetical protein CDS
293	PAVL33	QPB11831.1	14361	14597	237	forward	hypothetical protein CDS
294	PAVL33	QPB11819.1	10606	10836	231	forward	hypothetical protein CDS
295	PAVL33	QPB11836.1	17011	17238	228	reverse	hypothetical protein CDS
296	PAVL33	QPB11823.1	11467	11682	216	reverse	hypothetical protein CDS
297	PAVL33	QPB11821.1	11141	11347	207	forward	hypothetical protein CDS
298	PAVL33	QPB11820.1	10873	11076	204	forward	hypothetical protein CDS
299	PAVL33	QPB11839.1	17527	17730	204	forward	hypothetical protein CDS
300	PAVL33	QPB11827.1	13210	13407	198	reverse	hypothetical protein CDS
301	PAVL33	QPB11847.1	20875	21072	198	forward	hypothetical protein CDS
302	PAVL33	QPB11840.1	17878	18063	186	reverse	hypothetical protein CDS
303	PAVL33	QPB11825.1	12152	12322	171	forward	hypothetical protein CDS
304	PAVL33	QPB11843.1	19368	19526	159	forward	hypothetical protein CDS
305	PAVL33	QPB11835.1	16839	16982	144	forward	hypothetical protein CDS
306	PAVL33	QPB11845.1	20267	20389	123	reverse	hypothetical protein CDS

307	PAVL33	QPB11838.1	17414	17530	117	forward	hypothetical protein CDS
308	PAVL33	QPB11853.1	25413	25526	114	forward	hypothetical protein CDS
309	PAVL33	QPB11822.1	11347	11451	105	forward	hypothetical protein CDS
310	PAVL33	QPB11829.1	13840	13944	105	forward	hypothetical protein CDS
311	PAVL33	QPB11837.1	17277	17381	105	forward	hypothetical protein CDS
312	PAVL33	QPB11830.1	14085	14180	96	forward	hypothetical protein CDS
313	PAVL34	QPB11757.1	405	3170	2766	forward	hypothetical protein CDS
314	PAVL34	QPB11796.1	21914	23425	1512	forward	hypothetical protein CDS
315	PAVL34	QPB11797.1	23422	24741	1320	forward	capsid and scaffold protein CDS
316	PAVL34	QPB11759.1	4135	5292	1158	forward	hypothetical protein CDS
317	PAVL34	QPB11801.1	26142	27113	972	forward	major capsid protein CDS
318	PAVL34	QPB11779.1	13552	14505	954	reverse	hypothetical protein CDS
319	PAVL34	QPB11758.1	3186	4127	942	forward	hypothetical protein CDS
320	PAVL34	QPB11782.1	15469	16410	942	reverse	exonuclease CDS
321	PAVL34	QPB11760.1	5313	6131	819	forward	hypothetical protein CDS
322	PAVL34	QPB11764.1	7447	8262	816	forward	hypothetical protein CDS
323	PAVL34	QPB11798.1	24738	25502	765	forward	hypothetical protein CDS
324	PAVL34	QPB11777.1	12417	13088	672	reverse	DNA primase CDS
325	PAVL34	QPB11806.1	28684	29313	630	forward	hypothetical protein CDS
326	PAVL34	QPB11786.1	17322	17855	534	reverse	hypothetical protein CDS
327	PAVL34	QPB11800.1	25611	26135	525	forward	hypothetical protein CDS
328	PAVL34	QPB11766.1	8766	9287	522	reverse	hypothetical protein CDS
329	PAVL34	QPB11762.1	6455	6949	495	forward	tail fiber protein CDS
330	PAVL34	QPB11780.1	14552	15022	471	reverse	hypothetical protein CDS
331	PAVL34	QPB11802.1	27157	27618	462	forward	hypothetical protein CDS
332	PAVL34	QPB11769.1	10211	10660	450	forward	hypothetical protein CDS
333	PAVL34	QPB11795.1	21469	21909	441	forward	hypothetical protein CDS
334	PAVL34	QPB11765.1	8275	8679	405	forward	putative holin CDS
335	PAVL34	QPB11781.1	15068	15472	405	reverse	hypothetical protein CDS
336	PAVL34	QPB11763.1	6946	7347	402	forward	tail fiber protein CDS
337	PAVL34	QPB11775.1	11783	12181	399	forward	hypothetical protein CDS
338	PAVL34	QPB11767.1	9293	9685	393	reverse	hypothetical protein CDS
339	PAVL34	QPB11805.1	28261	28632	372	forward	hypothetical protein CDS
340	PAVL34	QPB11783.1	16410	16769	360	reverse	hypothetical protein CDS
341	PAVL34	QPB11788.1	18353	18664	312	reverse	hypothetical protein CDS
342	PAVL34	QPB11794.1	21170	21475	306	forward	hypothetical protein CDS
343	PAVL34	QPB11789.1	18681	18980	300	forward	hypothetical protein CDS
344	PAVL34	QPB11804.1	27974	28264	291	forward	hypothetical protein CDS
345	PAVL34	QPB11756.1	110	397	288	forward	hypothetical protein CDS
346	PAVL34	QPB11768.1	9689	9973	285	reverse	hypothetical protein CDS
347	PAVL34	QPB11761.1	6188	6451	264	forward	hypothetical protein CDS
348	PAVL34	QPB11791.1	19686	19931	246	reverse	hypothetical protein CDS
349	PAVL34	QPB11792.1	20539	20784	246	reverse	hypothetical protein CDS
350	PAVL34	QPB11770.1	10682	10912	231	forward	hypothetical protein CDS
351	PAVL34	QPB11785.1	17072	17299	228	reverse	hypothetical protein CDS
352	PAVL34	QPB11774.1	11543	11758	216	reverse	hypothetical protein CDS
353	PAVL34	QPB11772.1	11217	11423	207	forward	hypothetical protein CDS
354	PAVL34	QPB11771.1	10949	11152	204	forward	hypothetical protein CDS
355	PAVL34	QPB11784.1	16875	17075	201	reverse	hypothetical protein CDS
356	PAVL34	QPB11778.1	13286	13483	198	reverse	hypothetical protein CDS
357	PAVL34	QPB11793.1	20976	21173	198	forward	hypothetical protein CDS
358	PAVL34	QPB11787.1	18101	18268	168	forward	hypothetical protein CDS
359	PAVL34	QPB11790.1	19408	19566	159	forward	hypothetical protein CDS

360	PAVL34	QPB11803.1	27809	27967	159	forward	hypothetical protein CDS
361	PAVL34	QPB11776.1	12246	12398	153	forward	hypothetical protein CDS
362	PAVL34	QPB11799.1	25505	25618	114	forward	hypothetical protein CDS
363	PAVL34	QPB11773.1	11423	11527	105	forward	hypothetical protein CDS
364	PAVL45	QPB11862.1	405	3170	2766	forward	hypothetical protein CDS
365	PAVL45	QPB11899.1	22040	23551	1512	forward	hypothetical protein CDS
366	PAVL45	QPB11900.1	23548	24873	1326	forward	hypothetical protein CDS
367	PAVL45	QPB11864.1	4134	5297	1164	forward	hypothetical protein CDS
368	PAVL45	QPB11875.1	10181	11218	1038	reverse	hypothetical protein CDS
369	PAVL45	QPB11863.1	3186	4133	948	forward	hypothetical protein CDS
370	PAVL45	QPB11885.1	15602	16549	948	reverse	exonuclease CDS
371	PAVL45	QPB11904.1	26309	27256	948	forward	hypothetical protein CDS
372	PAVL45	QPB11865.1	5317	6135	819	forward	hypothetical protein CDS
373	PAVL45	QPB11867.1	6443	7249	807	forward	hypothetical protein CDS
374	PAVL45	QPB11901.1	24880	25635	756	forward	hypothetical protein CDS
375	PAVL45	QPB11881.1	13782	14522	741	reverse	hypothetical protein CDS
376	PAVL45	QPB11879.1	12557	13228	672	reverse	DNA primase CDS
377	PAVL45	QPB11868.1	7495	8148	654	forward	hypothetical protein CDS
378	PAVL45	QPB11909.1	28830	29429	600	forward	hypothetical protein CDS
379	PAVL45	QPB11878.1	11950	12513	564	reverse	hypothetical protein CDS
380	PAVL45	QPB11877.1	11393	11953	561	reverse	hypothetical protein CDS
381	PAVL45	QPB11889.1	17466	17999	534	reverse	hypothetical protein CDS
382	PAVL45	QPB11871.1	8647	9168	522	reverse	hypothetical protein CDS
383	PAVL45	QPB11905.1	27300	27764	465	forward	hypothetical protein CDS
384	PAVL45	QPB11903.1	25874	26302	429	forward	hypothetical protein CDS
385	PAVL45	QPB11890.1	18230	18646	417	forward	hypothetical protein CDS
386	PAVL45	QPB11884.1	15195	15605	411	reverse	hypothetical protein CDS
387	PAVL45	QPB11872.1	9173	9565	393	reverse	hypothetical protein CDS
388	PAVL45	QPB11898.1	21664	22035	372	forward	hypothetical protein CDS
389	PAVL45	QPB11908.1	28407	28778	372	forward	hypothetical protein CDS
390	PAVL45	QPB11886.1	16549	16908	360	reverse	hypothetical protein CDS
391	PAVL45	QPB11880.1	13426	13782	357	reverse	hypothetical protein CDS
392	PAVL45	QPB11906.1	27766	28113	348	forward	hypothetical protein CDS
393	PAVL45	QPB11874.1	9853	10173	321	reverse	hypothetical protein CDS
394	PAVL45	QPB11897.1	21295	21600	306	forward	hypothetical protein CDS
395	PAVL45	QPB11907.1	28120	28410	291	forward	hypothetical protein CDS
396	PAVL45	QPB11873.1	9569	9835	267	reverse	hypothetical protein CDS
397	PAVL45	QPB11866.1	6177	6440	264	forward	hypothetical protein CDS
398	PAVL45	QPB11892.1	18871	19119	249	forward	hypothetical protein CDS
399	PAVL45	QPB11894.1	19270	19503	234	reverse	hypothetical protein CDS
400	PAVL45	QPB11882.1	14580	14807	228	forward	hypothetical protein CDS
401	PAVL45	QPB11888.1	17215	17442	228	reverse	hypothetical protein CDS
402	PAVL45	QPB11891.1	18650	18871	222	forward	hypothetical protein CDS
403	PAVL45	QPB11887.1	17015	17218	204	reverse	hypothetical protein CDS
404	PAVL45	QPB11896.1	20952	21152	201	reverse	hypothetical protein CDS
405	PAVL45	QPB11876.1	11215	11409	195	reverse	hypothetical protein CDS
406	PAVL45	QPB11883.1	14819	15013	195	forward	hypothetical protein CDS
407	PAVL45	QPB11895.1	19738	19932	195	reverse	hypothetical protein CDS
408	PAVL45	QPB11902.1	25667	25858	192	reverse	hypothetical protein CDS
409	PAVL45	QPB11861.1	224	397	174	forward	hypothetical protein CDS
410	PAVL45	QPB11869.1	8214	8384	171	reverse	hypothetical protein CDS
411	PAVL45	QPB11870.1	8428	8562	135	forward	putative holin CDS
412	PAVL45	QPB11893.1	19141	19245	105	forward	hypothetical protein CDS

Table S3.

Phage and antibiotic sensitivity profiles of the clinical *C. acnes* isolates. S – Susceptible, I – Intermediate, R – Resistant.

Table S3.

Index	Isolate number	Fd1	Fd2	Fd3	PAVL20	PAVL21	PAVL33	PAVL34	PAVL45	Erythromycin	Clindamycin	Tetracycline	Doxycycline	Minocycline	Bacterial strain	SLST Type [26]	Phage isolated from sample?
**1**	**3**	R	R	R	R	R	R	R	R	S	S	S	S	S	*C. avdium*	N/A	No
**2**	**4**	R	R	R	R	R	R	R	R	S	S	S	S	S	*C. acnes*	C1	No
**3**	**20**	R	R	R	R	R	R	R	R	S	S	S	S	S	*C. avdium*	N/A	Yes
**4**	**21**	R	R	R	R	R	R	R	R	S	S	S	S	S	*C. acnes*	A1	Yes
**5**	**33**	R	R	R	R	R	R	R	R	S	S	S	S	S	*C. acnes*	H1	Yes
**6**	**34**	R	R	R	R	R	R	R	R	S	S	S	S	S	*C. avdium*	N/A	Yes
**7**	**39**	R	R	R	R	R	R	R	R	S	S	S	S	S	*C. granulosum*	N/A	No
**8**	**45**	R	R	R	R	R	R	R	R	S	S	S	S	S	*C. avdium*	N/A	Yes
**9**	**48**	R	R	R	R	R	R	R	R	S	S	S	S	S	*C. acnes*	Unkown	No
**10**	**14**	S	S	S	S	S	S	S	S	R	R	R	R	R	*C. acnes*	D1	No
**11**	**35**	S	S	S	S	S	S	S	S	R	R	R	R	R	*C. acnes*	A1	No
**12**	**5**	S	S	S	S	S	S	S	S	R	R	R	R	S	*C. acnes*	A1	No
**13**	**2**	S	S	S	S	S	S	S	S	R	R	I	R	I	*C. acnes*	A1	No
**14**	**31**	S	S	S	S	S	S	S	S	R	R	I	R	I	*C. acnes*	Unkown	No
**15**	**42**	S	S	S	S	S	S	S	S	R	R	I	R	I	*C. avdium*	N/A	No
**16**	**28**	S	S	S	S	S	S	S	S	R	R	I	R	S	*C. acnes*	A1	No
**17**	**25**	S	S	S	S	S	S	S	S	R	I	I	R	I	*C. acnes*	A1	No
**18**	**7**	S	S	S	S	S	S	S	S	R	I	I	I	S	*C. acnes*	C1	No
**19**	**24**	S	S	S	S	S	S	S	S	R	I	S	S	S	*C. acnes*	Unkown	No
**20**	**10**	S	S	S	S	S	S	S	S	S	S	S	S	R	*C. acnes*	A1	No
**21**	**23**	S	S	S	S	S	S	S	S	S	S	S	S	R	*C. acnes*	A1	No
**22**	**13**	S	S	S	S	S	S	S	S	S	S	I	S	S	*C. acnes*	C1	No
**23**	**19**	S	S	S	S	S	S	S	S	S	S	S	I	S	*C. acnes*	Unkown	No
**24**	**1**	S	S	S	S	S	S	S	S	S	S	S	S	S	*C. acnes*	A1	No
**25**	**6**	S	S	S	S	S	S	S	S	S	S	S	S	S	*C. acnes*	A1	No
**26**	**8**	S	S	S	S	S	S	S	S	S	S	S	S	S	*C. acnes*	C1	No
**27**	**9**	S	S	S	S	S	S	S	S	S	S	S	S	S	*C. acnes*	C2	No
**28**	**11**	S	S	S	S	S	S	S	S	S	S	S	S	S	*C. acnes*	A1	No
**29**	**15**	S	S	S	S	S	S	S	S	S	S	S	S	S	*C. acnes*	F4	No
**30**	**16**	S	S	S	S	S	S	S	S	S	S	S	S	S	*C. avdium*	N/A	No
**31**	**18**	S	S	S	S	S	S	S	S	S	S	S	S	S	*C. acnes*	A1	No
**32**	**22**	S	S	S	S	S	S	S	S	S	S	S	S	S	*C. acnes*	A1	No
**33**	**27**	S	S	S	S	S	S	S	S	S	S	S	S	S	*C. acnes*	H1	No
**34**	**30**	S	S	S	S	S	S	S	S	S	S	S	S	S	*C. acnes*	D1	No
**35**	**32**	S	S	S	S	S	S	S	S	S	S	S	S	S	*C. acnes*	Unkown	No
**36**	**36**	S	S	S	S	S	S	S	S	S	S	S	S	S	*C. acnes*	A1	No
**37**	**37**	S	S	S	S	S	S	S	S	S	S	S	S	S	*C. acnes*	A1	No
**38**	**40**	S	S	S	S	S	S	S	S	S	S	S	S	S	*C. acnes*	Unkown	No
**39**	**41**	S	S	S	S	S	S	S	S	S	S	S	S	S	*C. acnes*	A1	No
**40**	**43**	S	S	S	S	S	S	S	S	S	S	S	S	S	*C. acnes*	K1	No
**41**	**44**	S	S	S	S	S	S	S	S	S	S	S	S	S	*C. acnes*	F4	No
**42**	**46**	S	S	S	S	S	S	S	S	S	S	S	S	S	*C. avdium*	N/A	No
**43**	**47**	S	S	S	S	S	S	S	S	S	S	S	S	S	*C. acnes*	A1	No
**44**	**49**	S	S	S	S	S	S	S	S	S	S	S	S	S	*C. acnes*	F4	No

Table S4.

Daily scoring and photography of the 4 sham-injected, 17 infected-untreated and 17 FD3 phage-treated mice for the eight days of the experiment. L- left lesion, R- right lesion. The diameter score was calculated using an electronic caliper. The elevation of the eschar scores were assessed by two different blinded scorers in all mice except the sham-injected mice, which were not blinded. The total score is the sum of the individual scores. Images were obtained from the same location and in the same room with similar lighting using the same camera. However, owing to the movement of mice, it was impossible to achieve identical angles and backgrounds. For more details, refer to the assessment of phage clinical efficacy section in Materials and Methods and Supplemental Fig. S6.

<u>Table S4.</u>

<u>Sham Injection Control</u>

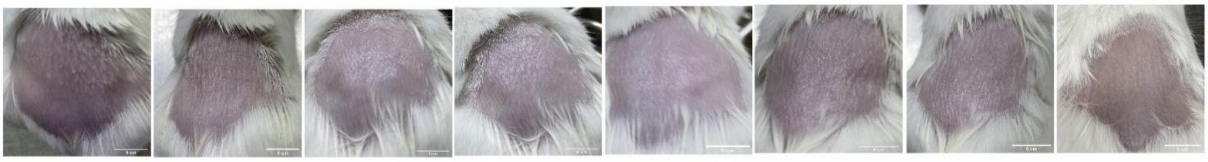

# Animal : 1		Day 3		Day 4		Day 5		Day 6		Day 7		Day 8		Day 9		Day 10	
		L	R	L	R	L	R	L	R	L	R	L	R	L	R	L	R
Diameter Score	measured	1	1	2	0	0	0	0	0	0	0	0	0	0	0	0	0
Elevation Score	first scorer	2	0	1	0	0	0	1	0	0	0	0	0	0	0	0	0
	second scorer	2	1	0	0	1	0	0	0	0	0	0	0	0	0	0	0
Eschar Score	first scorer	0	0	0	0	0	0	0	0	0	0	0	0	0	0	0	0
	second scorer	0	0	0	0	0	0	0	0	0	0	0	0	0	0	0	0
Total Score	averaged	3	1.5	2.5	0	0.5	0	0.5	0	0	0	0	0	0	0	0	0

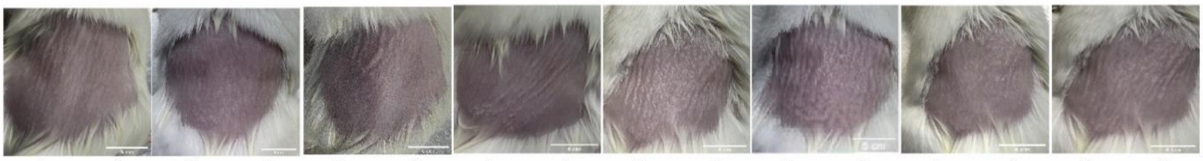

# Animal : 2		Day 3		Day 4		Day 5		Day 6		Day 7		Day 8		Day 9		Day 10	
		L	R	L	R	L	R	L	R	L	R	L	R	L	R	L	R
Diameter Score	measured	1	1	1	1	0	0	0	0	0	0	0	0	0	0	0	0
Elevation Score	first scorer	1	0	1	0	0	0	1	0	0	0	0	0	0	0	0	0
	second scorer	1	1	0	0	1	0	0	0	0	0	0	0	0	0	0	0
Eschar Score	first scorer	0	0	0	0	0	0	0	0	0	0	0	0	0	0	0	0
	second scorer	0	0	0	0	0	0	0	0	0	0	0	0	0	0	0	0
Total Score	averaged	2	1.5	2.5	0	0.5	0	0.5	0	0	0	0	0	0	0	0	0

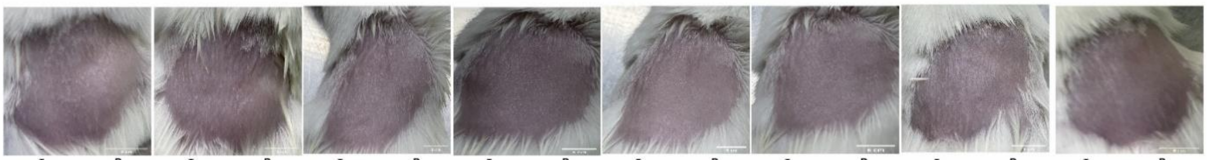

# Animal : 3		Day 3		Day 4		Day 5		Day 6		Day 7		Day 8		Day 9		Day 10	
		L	R	L	R	L	R	L	R	L	R	L	R	L	R	L	R
Diameter Score	measured	1	2	1	2	0	1	0	0	0	0	0	0	0	0	0	0
Elevation Score	first scorer	1	2	1	1	1	0	1	1	0	0	0	0	0	0	0	0
	second scorer	1	2	0	1	0	1	0	0	0	0	0	0	0	0	0	0
Eschar Score	first scorer	0	0	0	0	0	0	0	0	0	0	0	0	0	0	0	0
	second scorer	0	0	0	0	0	0	0	0	0	0	0	0	0	0	0	0
Total Score	averaged	1	2.5	1.5	2	0.5	1.5	0.5	0.5	0	0	0	0	0	0	0	0

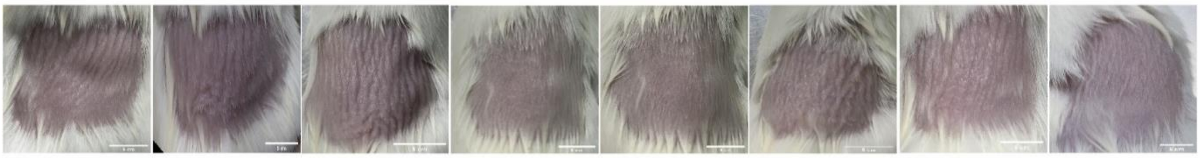

# Animal : 4		Day 3		Day 4		Day 5		Day 6		Day 7		Day 8		Day 9		Day 10	
		L	R	L	R	L	R	L	R	L	R	L	R	L	R	L	R
Diameter Score	measured	0	1	0	0	0	0	0	0	0	0	0	0	0	0	0	0
Elevation Score	first scorer	0	2	0	0	0	0	0	1	0	0	0	0	0	0	0	0
	second scorer	1	2	0	0	0	0	0	1	0	0	0	0	0	0	0	0
Eschar Score	first scorer	0	0	0	0	0	0	0	0	0	0	0	0	0	0	0	0
	second scorer	0	0	0	0	0	0	0	0	0	0	0	0	0	0	0	0
Total Score	averaged	0.5	1.5	0	0	0	0	0	1	0	0	0	0	0	0	0	0

Infected-Untreated

# Animal : 1

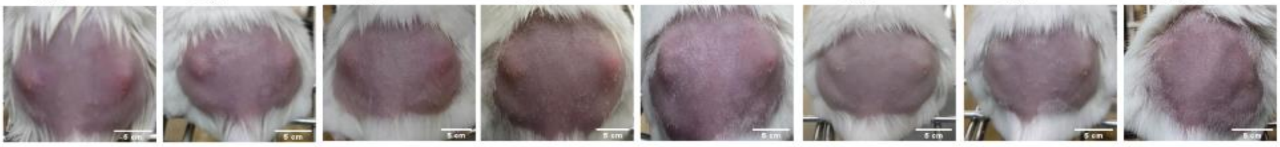

		Day 3		Day 4		Day 5		Day 6		Day 7		Day 8		Day 9		Day 10	
		**L**	**R**	**L**	**R**	**L**	**R**	**L**	**R**	**L**	**R**	**L**	**R**	**L**	**R**	**L**	**R**
**Diameter Score**	measured	2	2	1	1	2	2	2	2	2	2	2	2	2	1	1	2
**Elevation Score**	first scorer	1	1	1	1	1	1	2	2	1	1	1	1	1	1	0	1
	second scorer	2	2	1	1	2	1	1	2	1	1	1	1	0	1	0	1
**Eschar Score**	first scorer	0	0	0	1	0	0	0	0	0	1	0	0	0	1	0	0
	second scorer	0	0	0	1	0	0	0	0	0	1	0	1	0	0	0	0
**Total Score**	averaged	**3.5**	**3.5**	**2**	**3**	**3.5**	**3**	**3.5**	**4**	**3**	**4**	**3**	**3.5**	**2.5**	**2.5**	**1**	**3**

# Animal : 2

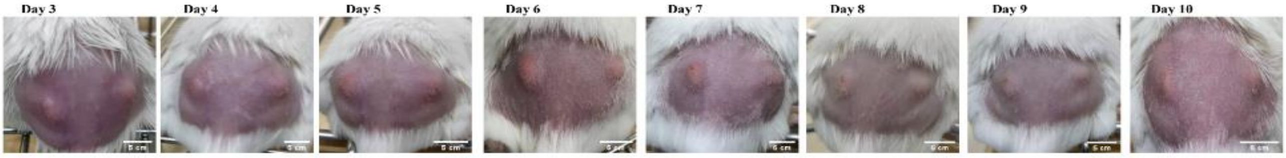

		Day 3		Day 4		Day 5		Day 6		Day 7		Day 8		Day 9		Day 10	
		**L**	**R**	**L**	**R**	**L**	**R**	**L**	**R**	**L**	**R**	**L**	**R**	**L**	**R**	**L**	**R**
**Diameter Score**	measured	2	3	2	2	3	2	3	1	3	2	2	1	2	2	1	1
**Elevation Score**	first scorer	2	2	2	2	2	2	2	1	2	1	1	1	1	1	1	1
	second scorer	2	2	2	2	2	2	2	2	1	1	1	1	2	1	2	1
**Eschar Score**	first scorer	0	0	0	0	0	0	0	0	1	0	1	0	1	0	0	0
	second scorer	0	0	0	0	0	0	0	0	1	0	1	0	1	0	0	0
**Total Score**	averaged	**4**	**5**	**4**	**4**	**5**	**4**	**5**	**2.5**	**5.5**	**3**	**4**	**2**	**4.5**	**3**	**2.5**	**2**

# Animal : 3

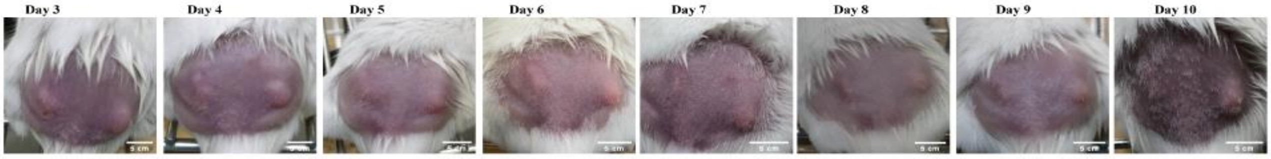

		Day 3		Day 4		Day 5		Day 6		Day 7		Day 8		Day 9		Day 10	
		**L**	**R**	**L**	**R**	**L**	**R**	**L**	**R**	**L**	**R**	**L**	**R**	**L**	**R**	**L**	**R**
**Diameter Score**	measured	2	2	2	2	2	2	2	2	2	3	2	2	1	2	1	2
**Elevation Score**	first scorer	1	2	1	2	1	2	1	2	0	1	1	1	1	1	1	1
	second scorer	1	2	1	2	1	2	1	2	0	2	1	2	2	2	0	2
**Eschar Score**	first scorer	0	0	0	0	0	0	0	0	1	0	0	0	0	0	0	0
	second scorer	0	0	0	0	0	0	0	0	1	0	0	0	0	0	0	0
**Total Score**	averaged	**3**	**4**	**3**	**4**	**3**	**4**	**3**	**4**	**3**	**4.5**	**3**	**3.5**	**2.5**	**3.5**	**1.5**	**3.5**

# Animal : 4

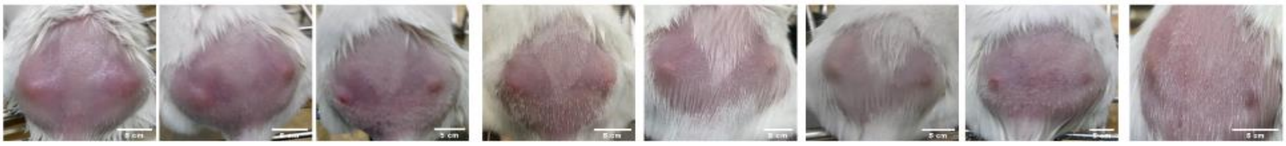

		Day 3		Day 4		Day 5		Day 6		Day 7		Day 8		Day 9		Day 10	
		**L**	**R**	**L**	**R**	**L**	**R**	**L**	**R**	**L**	**R**	**L**	**R**	**L**	**R**	**L**	**R**
**Diameter Score**	measured	2	2	2	2	2	2	2	2	2	2	2	2	2	2	1	1
**Elevation Score**	first scorer	2	2	1	1	1	1	1	1	1	1	1	1	0	1	0	0
	second scorer	2	1	1	1	1	1	1	1	1	1	1	1	1	0	1	1
**Eschar Score**	first scorer	0	0	0	0	0	0	0	0	0	0	0	0	0	0	0	0
	second scorer	0	0	0	0	0	0	0	0	0	0	0	0	0	0	0	0
**Total Score**	averaged	**4**	**3.5**	**3**	**3**	**3**	**3**	**3**	**3**	**3**	**3**	**3**	**3**	**2.5**	**2.5**	**1.5**	**1.5**

# Animal : 5

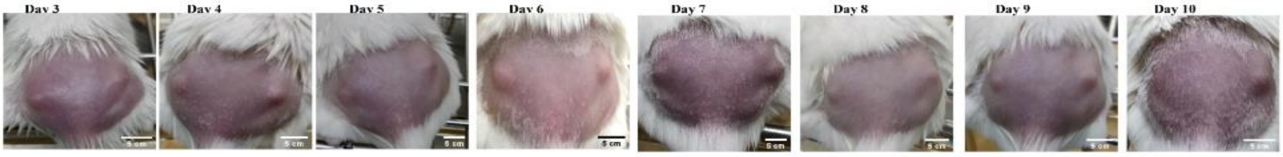

		Day 3		Day 4		Day 5		Day 6		Day 7		Day 8		Day 9		Day 10	
		**L**	**R**	**L**	**R**	**L**	**R**	**L**	**R**	**L**	**R**	**L**	**R**	**L**	**R**	**L**	**R**
**Diameter Score**	measured	2	2	2	2	1	1	1	1	2	2	2	2	2	2	2	2
**Elevation Score**	first scorer	1	1	1	2	1	2	2	2	1	2	1	1	2	2	1	1
	second scorer	2	1	1	1	1	1	2	2	2	1	2	2	1	1	1	2
**Eschar Score**	first scorer	0	0	0	0	0	0	0	0	0	0	0	0	0	0	0	0
	second scorer	0	0	0	0	0	0	0	0	0	0	0	0	0	0	0	0
**Total Score**	averaged	**3.5**	**3**	**3**	**3.5**	**2**	**2.5**	**3**	**3**	**3.5**	**3.5**	**3.5**	**3.5**	**3.5**	**3.5**	**3**	**3.5**

# Animal : 6

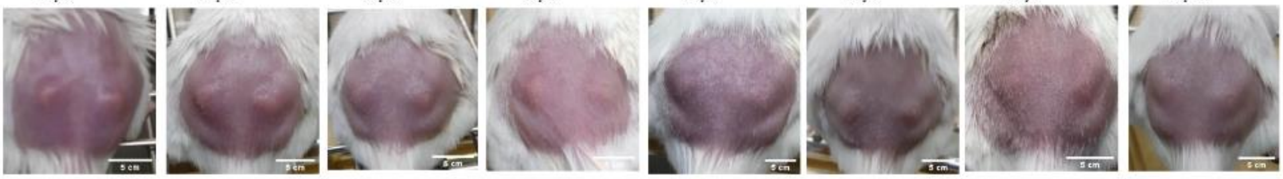

		Day 3		Day 4		Day 5		Day 6		Day 7		Day 8		Day 9		Day 10	
		L	R	L	R	L	R	L	R	L	R	L	R	L	R	L	R
**Diameter Score**	measured	1	1	2	2	1	1	1	1	2	2	2	1	1	1	2	2
**Elevation Score**	first scorer	1	2	2	2	2	2	2	2	2	1	2	3	1	2	1	1
	second scorer	2	1	2	2	2	2	2	2	1	1	2	2	1	2	2	2
**Eschar Score**	first scorer	0	0	0	0	0	0	0	0	0	0	0	1	0	1	0	1
	second scorer	0	0	0	0	0	0	0	0	0	0	0	0	0	0	0	1
**Total Score**	averaged	**2.5**	**2.5**	**4**	**4**	**3**	**3**	**3**	**3**	**3.5**	**3**	**4**	**4**	**2**	**3.5**	**3.5**	**4.5**

# Animal : 7

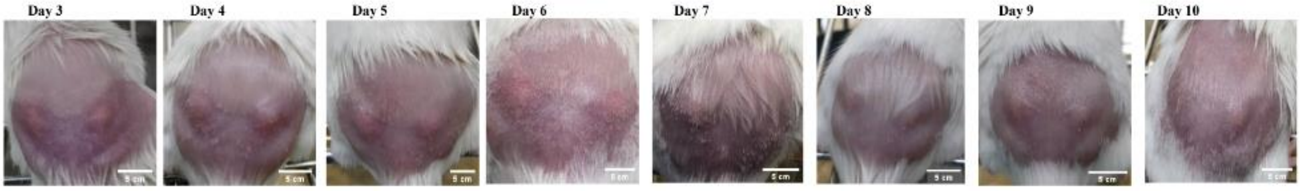

		Day 3		Day 4		Day 5		Day 6		Day 7		Day 8		Day 9		Day 10	
		L	R	L	R	L	R	L	R	L	R	L	R	L	R	L	R
**Diameter Score**	measured	2	1	3	2	3	2	3	3	2	1	2	2	1	2	1	1
**Elevation Score**	first scorer	1	1	1	2	2	1	1	0	2	1	1	1	1	1	1	1
	second scorer	1	2	1	2	2	1	0	1	2	0	1	2	2	2	1	2
**Eschar Score**	first scorer	0	0	0	0	0	0	1	0	0	0	0	0	0	0	1	0
	second scorer	0	0	0	0	0	0	0	0	0	0	0	0	0	0	0	0
**Total Score**	averaged	**3**	**2.5**	**4**	**4**	**5**	**3**	**4**	**3.5**	**4**	**1.5**	**3**	**3.5**	**2.5**	**3.5**	**2.5**	**2.5**

# Animal : 8

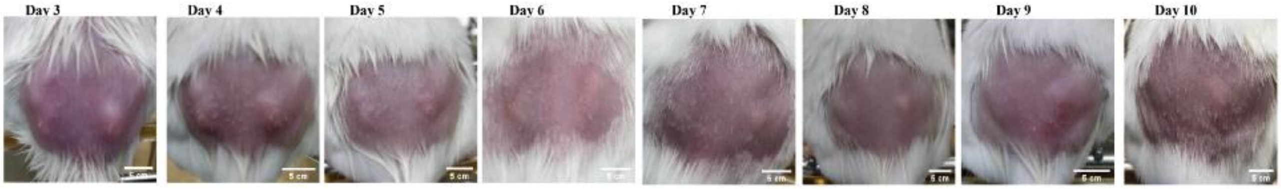

		Day 3		Day 4		Day 5		Day 6		Day 7		Day 8		Day 9		Day 10	
		L	R	L	R	L	R	L	R	L	R	L	R	L	R	L	R
**Diameter Score**	measured	2	2	2	2	3	3	3	2	2	1	2	2	2	2	2	2
**Elevation Score**	first scorer	1	2	2	2	1	1	1	1	0	1	0	1	1	1	1	1
	second scorer	1	2	2	2	1	1	0	1	0	1	1	2	1	2	1	2
**Eschar Score**	first scorer	0	0	0	0	0	0	0	0	0	0	0	0	0	0	0	0
	second scorer	0	0	0	0	0	0	0	0	0	0	0	0	0	0	0	0
**Total Score**	averaged	**3**	**4**	**4**	**4**	**4**	**4**	**3.5**	**3**	**2**	**2**	**2.5**	**3.5**	**3**	**3.5**	**3**	**3.5**

# Animal : 9

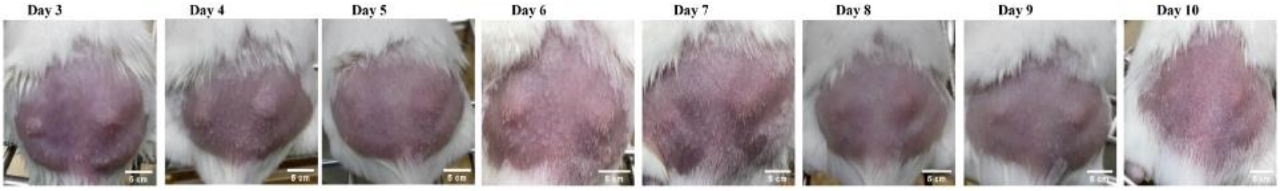

		Day 3		Day 4		Day 5		Day 6		Day 7		Day 8		Day 9		Day 10	
		L	R	L	R	L	R	L	R	L	R	L	R	L	R	L	R
**Diameter Score**	measured	1	2	1	2	2	2	2	3	1	2	2	1	2	2	2	1
**Elevation Score**	first scorer	1	2	1	2	0	0	2	1	0	2	1	1	1	1	1	1
	second scorer	2	2	2	2	0	0	2	1	0	1	1	1	1	2	1	2
**Eschar Score**	first scorer	0	0	0	0	0	0	0	0	0	0	0	0	0	0	0	0
	second scorer	0	0	0	0	0	0	0	0	0	0	0	0	0	0	0	0
**Total Score**	averaged	**2.5**	**4**	**2.5**	**4**	**2**	**2**	**4**	**4**	**1**	**3.5**	**3**	**2**	**3**	**3.5**	**3**	**2.5**

# Animal : 10

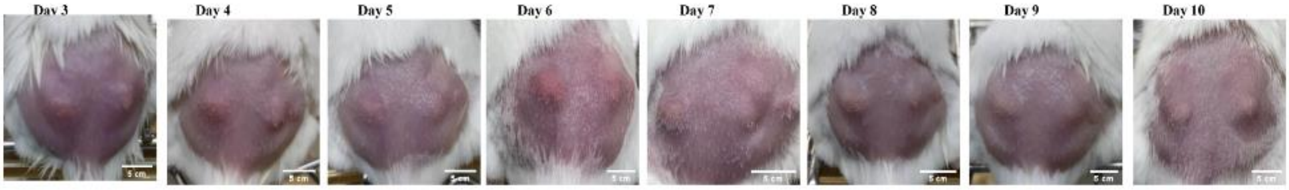

		Day 3		Day 4		Day 5		Day 6		Day 7		Day 8		Day 9		Day 10	
		L	R	L	R	L	R	L	R	L	R	L	R	L	R	L	R
**Diameter Score**	measured	2	2	2	1	2	1	2	2	1	1	2	2	2	2	2	2
**Elevation Score**	first scorer	2	2	2	2	1	2	1	1	2	2	2	2	2	2	2	2
	second scorer	2	2	2	2	2	2	1	1	2	2	2	2	2	2	2	2
**Eschar Score**	first scorer	0	0	0	0	0	0	0	0	0	0	0	0	0	0	1	0
	second scorer	0	0	0	0	0	0	0	0	0	0	0	0	0	0	0	0
**Total Score**	averaged	**4**	**4**	**4**	**3**	**3.5**	**3**	**3**	**3**	**3**	**3**	**4**	**4**	**4**	**4**	**4.5**	**4**

Day 3 Day 4 Day 5 Day 6 Day 7 Day 8 Day 9 Day 10

# Animal : 11

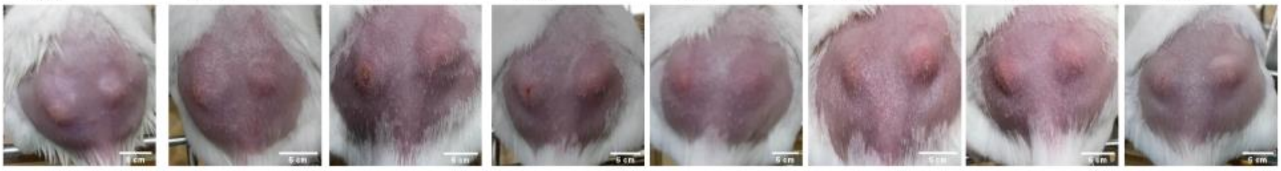

		L	R	L	R	L	R	L	R	L	R	L	R	L	R	L	R
**Diameter Score**	measured	2	2	2	2	2	2	2	3	2	2	2	2	2	2	2	3
**Elevation Score**	first scorer	2	3	1	2	2	2	2	3	2	2	2	2	2	2	2	3
	second scorer	3	3	2	1	2	2	2	3	1	2	1	2	2	2	2	2
**Eschar Score**	first scorer	0	0	0	0	1	0	1	0	0	0	0	0	0	0	1	0
	second scorer	0	0	0	0	1	0	1	0	0	0	0	0	0	0	0	0
**Total Score**	averaged	**4.5**	**5**	**3.5**	**3.5**	**5**	**4**	**5**	**6**	**3.5**	**4**	**3.5**	**4**	**4**	**4**	**4.5**	**5.5**

Day 3 Day 4 Day 5 Day 6 Day 7 Day 8 Day 9 Day 10

# Animal : 12

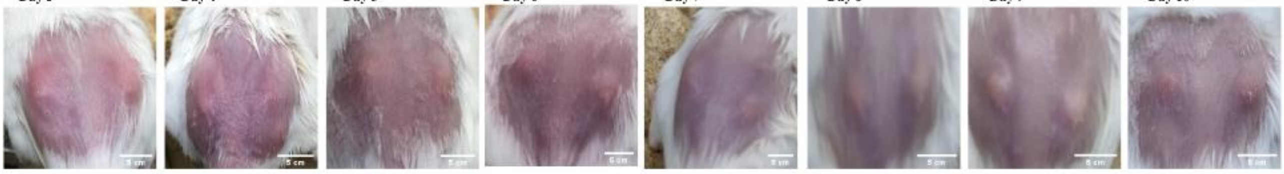

		L	R	L	R	L	R	L	R	L	R	L	R	L	R	L	R
**Diameter Score**	measured	2	2	3	2	3	2	2	2	2	2	2	2	2	2	2	2
**Elevation Score**	first scorer	2	1	1	2	1	2	2	2	2	1	1	1	3	2	2	1
	second scorer	1	1	1	2	1	2	1	2	2	2	2	2	2	1	2	1
**Eschar Score**	first scorer	0	0	0	0	0	0	0	0	0	0	0	0	0	0	0	0
	second scorer	0	0	0	0	0	0	0	0	0	0	0	0	0	0	0	0
**Total Score**	averaged	**3.5**	**3**	**4**	**4**	**4**	**4**	**3.5**	**4**	**4**	**3.5**	**3.5**	**3.5**	**4.5**	**3.5**	**4**	**3**

Day 3 Day 4 Day 5 Day 6 Day 7 Day 8 Day 9 Day 10

# Animal : 13

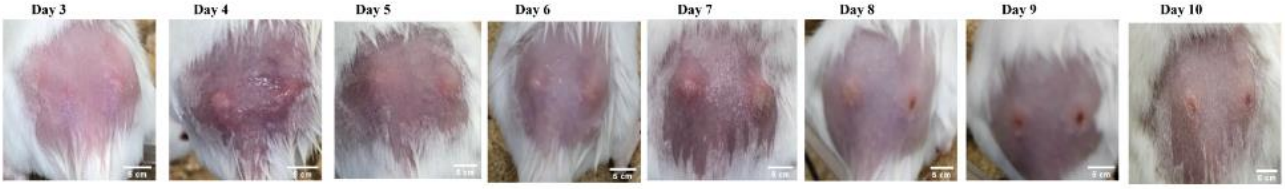

		L	R	L	R	L	R	L	R	L	R	L	R	L	R	L	R
**Diameter Score**	measured	2	2	2	2	2	2	2	2	2	2	2	2	2	2	1	2
**Elevation Score**	first scorer	2	2	2	1	2	2	2	1	2	2	2	1	1	1	1	1
	second scorer	2	1	1	1	2	2	1	1	3	2	2	1	1	1	1	1
**Eschar Score**	first scorer	0	0	0	0	0	0	0	0	0	0	0	1	1	2	1	2
	second scorer	0	0	0	0	0	0	0	0	0	0	0	1	1	2	1	2
**Total Score**	averaged	**4**	**3.5**	**3.5**	**3**	**4**	**4**	**3.5**	**3**	**4.5**	**4**	**4**	**4**	**4**	**5**	**3**	**5**

Day 3 Day 4 Day 5 Day 6 Day 7 Day 8 Day 9 Day 10

# Animal : 14

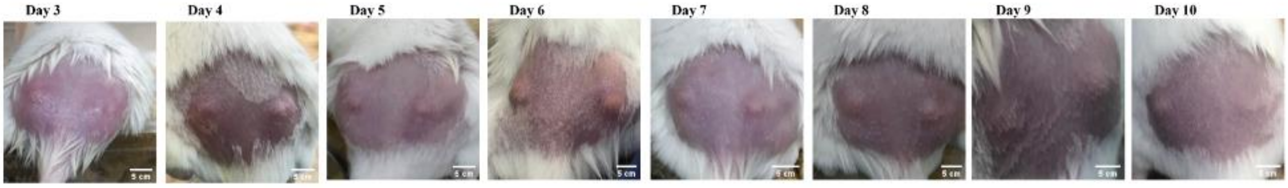

		L	R	L	R	L	R	L	R	L	R	L	R	L	R	L	R
**Diameter Score**	measured	2	2	2	2	2	3	2	3	2	3	3	3	2	3	2	3
**Elevation Score**	first scorer	0	0	1	1	1	1	2	1	1	1	2	1	0	1	1	0
	second scorer	0	1	1	2	1	1	1	2	2	2	1	1	1	1	1	1
**Eschar Score**	first scorer	0	0	0	0	0	0	0	0	0	0	0	0	0	0	0	0
	second scorer	0	0	0	0	0	0	0	0	0	0	0	0	0	0	0	0
**Total Score**	averaged	**2**	**2.5**	**3**	**3.5**	**3**	**4**	**3.5**	**4.5**	**3.5**	**4.5**	**4.5**	**4**	**2.5**	**4**	**3**	**3.5**

Day 3 Day 4 Day 5 Day 6 Day 7 Day 8 Day 9 Day 10

# Animal : 15

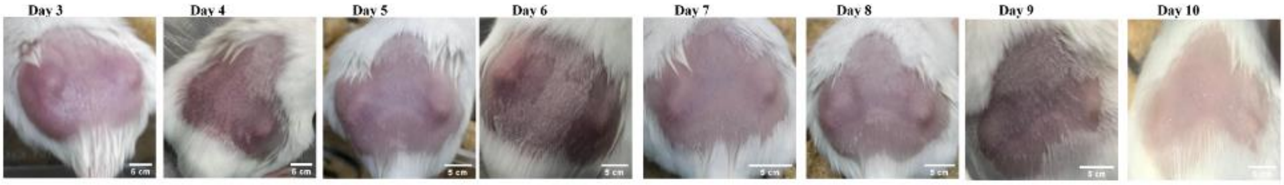

		L	R	L	R	L	R	L	R	L	R	L	R	L	R	L	R
**Diameter Score**	measured	2	1	2	2	2	2	2	2	1	1	1	2	1	2	1	2
**Elevation Score**	first scorer	2	2	1	1	1	2	1	1	2	1	2	1	1	1	1	1
	second scorer	2	2	0	1	1	2	2	1	2	2	2	2	1	1	1	1
**Eschar Score**	first scorer	0	0	0	0	0	0	0	0	0	0	0	0	0	0	0	0
	second scorer	0	0	0	0	0	0	0	0	0	0	0	0	0	0	0	0
**Total Score**	averaged	**4**	**3**	**2.5**	**3**	**3**	**4**	**3.5**	**3**	**3**	**2.5**	**3**	**3.5**	**2**	**3**	**2**	**3**

# Animal : 16

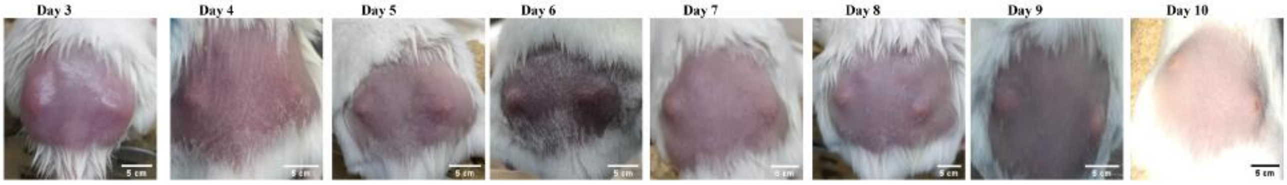

		L	R	L	R	L	R	L	R	L	R	L	R	L	R	L	R
**Diameter Score**	measured	2	2	2	1	2	2	2	2	2	2	1	2	2	2	1	2
**Elevation Score**	first scorer	1	2	1	1	1	2	1	2	1	1	1	1	1	1	1	1
	second scorer	2	2	1	1	1	1	2	1	1	1	1	1	2	2	0	1
**Eschar Score**	first scorer	0	0	0	0	0	0	0	0	0	0	0	0	0	0	1	2
	second scorer	0	0	0	0	0	0	0	0	0	0	0	0	0	0	1	1
**Total Score**	averaged	**3.5**	**4**	**3**	**2**	**3**	**3.5**	**3.5**	**3.5**	**3**	**3**	**2**	**3**	**3.5**	**3.5**	**2.5**	**4.5**

# Animal : 17

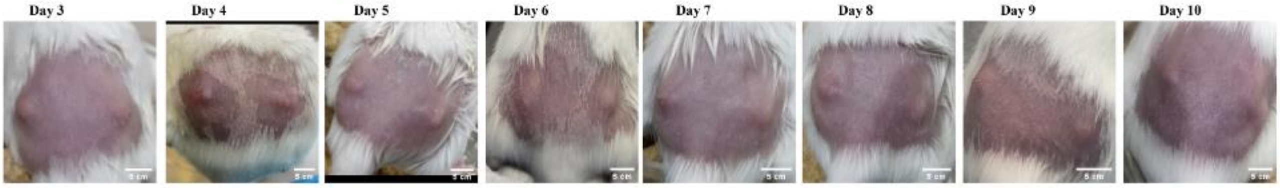

		L	R	L	R	L	R	L	R	L	R	L	R	L	R	L	R
**Diameter Score**	measured	2	1	3	2	2	2	2	3	2	2	2	3	1	2	2	2
**Elevation Score**	first scorer	1	1	1	0	1	1	1	1	2	2	2	2	1	2	1	2
	second scorer	1	2	2	1	2	2	1	1	1	1	1	1	2	2	2	2
**Eschar Score**	first scorer	0	1	1	0	0	0	0	0	0	0	0	0	0	0	0	0
	second scorer	0	0	0	0	0	0	0	0	0	0	0	0	0	0	0	0
**Total Score**	averaged	**3**	**3**	**5**	**2.5**	**3.5**	**3.5**	**3**	**4**	**3.5**	**3.5**	**3.5**	**4.5**	**2.5**	**4**	**3.5**	**4**

FD3 phage-treated group

Day 3 | Day 4 | Day 5 | Day 6 | Day 7 | Day 8 | Day 9 | Day 10

\# Animal : 1

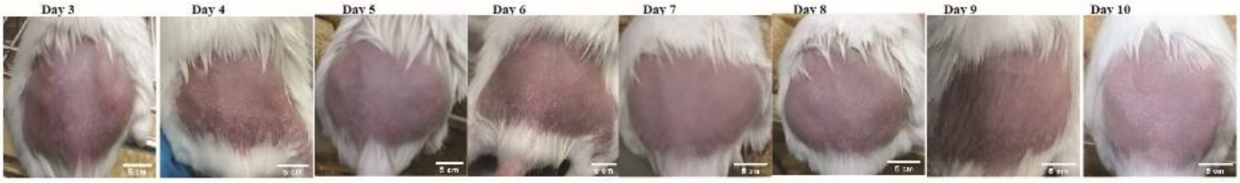

		L	R	L	R	L	R	L	R	L	R	L	R	L	R	L	R
**Diameter Score**	measured	1	2	2	1	2	2	1	1	1	0	1	0	1	0	1	0
**Elevation Score**	first scorer	1	1	2	0	1	0	1	1	0	0	1	1	1	0	1	0
	second scorer	2	2	1	0	1	0	0	0	1	0	1	1	1	0	1	1
**Eschar Score**	first scorer	0	0	0	0	0	0	0	0	0	0	0	0	0	0	0	0
	second scorer	0	0	0	0	0	0	0	0	0	0	0	0	0	0	0	0
**Total Score**	averaged	2.5	3.5	3.5	1	3	2	1.5	1.5	1.5	0	2	1	2	0	2	0.5

Day 3 | Day 4 | Day 5 | Day 6 | Day 7 | Day 8 | Day 9 | Day 10

\# Animal : 2

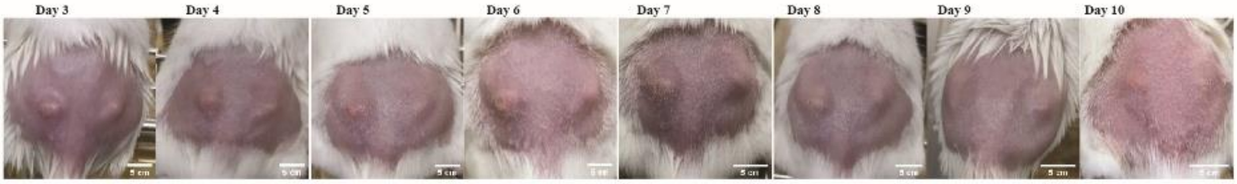

		L	R	L	R	L	R	L	R	L	R	L	R	L	R	L	R
**Diameter Score**	measured	3	1	2	1	2	1	2	1	2	1	2	1	1	1	1	1
**Elevation Score**	first scorer	2	2	2	1	2	2	2	1	2	1	2	1	1	2	1	1
	second scorer	2	2	2	2	2	1	2	2	2	2	2	2	1	1	1	1
**Eschar Score**	first scorer	0	0	0	0	0	0	1	0	1	0	0	0	0	0	0	0
	second scorer	0	0	0	0	0	0	0	0	0	0	0	0	0	0	0	0
**Total Score**	averaged	5	3	4	2.5	4	2.5	4.5	2.5	4.5	2.5	4	2.5	2	2.5	2	2

Day 3 | Day 4 | Day 5 | Day 6 | Day 7 | Day 8 | Day 9 | Day 10

\# Animal : 3

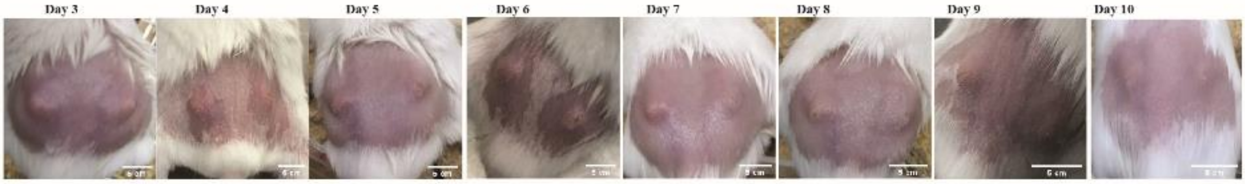

		L	R	L	R	L	R	L	R	L	R	L	R	L	R	L	R
**Diameter Score**	measured	3	2	2	1	2	1	1	1	1	1	1	1	1	1	1	0
**Elevation Score**	first scorer	2	2	1	1	1	1	1	1	1	0	1	1	1	1	1	0
	second scorer	2	2	0	1	1	0	0	1	1	1	1	1	1	0	1	1
**Eschar Score**	first scorer	0	0	0	0	0	0	0	0	0	0	0	0	0	0	0	0
	second scorer	0	0	0	0	0	0	0	1	0	0	1	1	0	0	0	0
**Total Score**	averaged	5	4	2.5	2	3	1.5	1.5	2.5	2	1.5	2.5	2.5	2	1.5	2	0.5

Day 3 | Day 4 | Day 5 | Day 6 | Day 7 | Day 8 | Day 9 | Day 10

\# Animal : 4

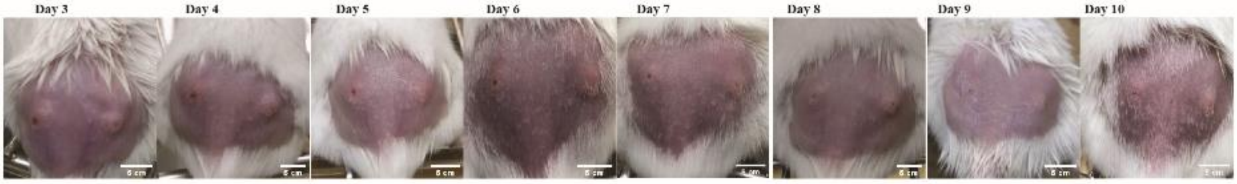

		L	R	L	R	L	R	L	R	L	R	L	R	L	R	L	R
**Diameter Score**	measured	2	2	2	2	1	2	1	2	1	1	2	2	1	1	1	1
**Elevation Score**	first scorer	1	1	0	2	1	1	1	1	2	1	1	2	1	1	1	1
	second scorer	2	1	1	2	0	1	1	1	1	1	1	2	1	1	0	1
**Eschar Score**	first scorer	2	1	1	0	1	0	1	0	1	0	2	0	0	0	1	1
	second scorer	1	1	2	0	2	0	1	0	2	1	1	1	1	0	0	0
**Total Score**	averaged	5	4	4	4	3	3	3	3	4	2.5	4.5	4.5	2.5	2	2	2.5

Day 3 | Day 4 | Day 5 | Day 6 | Day 7 | Day 8 | Day 9 | Day 10

\# Animal : 5

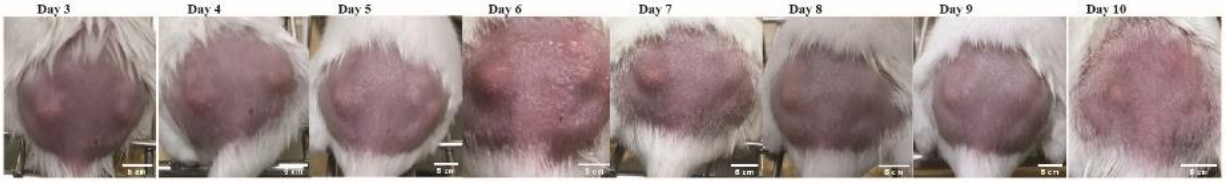

		L	R	L	R	L	R	L	R	L	R	L	R	L	R	L	R
**Diameter Score**	measured	2	1	2	1	2	2	2	1	2	1	2	2	2	1	2	0
**Elevation Score**	first scorer	2	2	2	2	1	1	2	1	2	1	2	2	1	1	1	1
	second scorer	2	2	1	1	2	1	1	0	2	2	2	1	1	1	0	0
**Eschar Score**	first scorer	0	0	0	0	0	0	0	0	0	0	0	0	0	0	0	0
	second scorer	0	0	0	0	0	0	0	0	0	0	0	0	0	0	0	0
**Total Score**	averaged	4	3	3.5	2	4	3	3.5	1.5	4	2.5	4	3.5	3	2	2.5	0.5

# Animal : 6

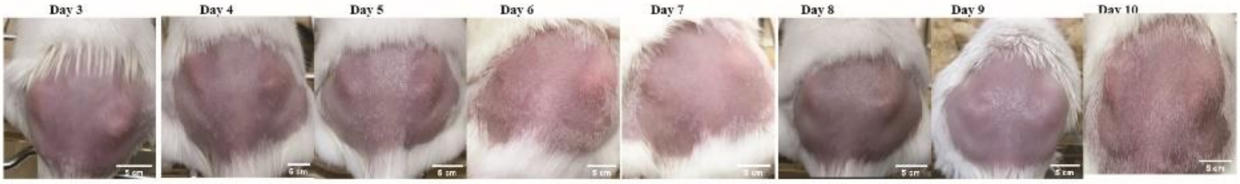

		Day 3		Day 4		Day 5		Day 6		Day 7		Day 8		Day 9		Day 10	
		L	R	L	R	L	R	L	R	L	R	L	R	L	R	L	R
**Diameter Score**	measured	2	2	2	2	2	3	1	2	1	2	1	2	1	2	1	2
**Elevation Score**	first scorer	2	1	1	2	0	2	1	2	2	2	1	1	0	1	1	0
	second scorer	1	2	0	1	1	1	0	1	1	1	1	2	1	2	0	1
**Eschar Score**	first scorer	0	0	0	0	0	0	0	0	0	0	0	0	0	0	0	0
	second scorer	0	0	0	0	0	0	0	0	0	0	0	0	0	0	0	0
**Total Score**	averaged	3.5	3.5	2.5	3.5	2.5	4.5	1.5	3.5	2.5	3.5	2	3.5	1.5	3.5	1.5	2.5

# Animal : 7

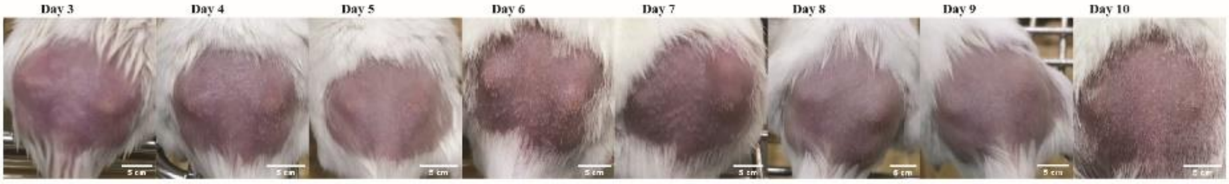

		Day 3		Day 4		Day 5		Day 6		Day 7		Day 8		Day 9		Day 10	
		L	R	L	R	L	R	L	R	L	R	L	R	L	R	L	R
**Diameter Score**	measured	2	1	2	1	2	1	1	1	1	1	1	2	1	2	1	1
**Elevation Score**	first scorer	2	2	0	2	1	2	0	1	1	1	1	1	1	0	1	1
	second scorer	1	1	1	1	1	1	1	2	1	1	1	2	1	0	1	0
**Eschar Score**	first scorer	0	0	0	0	0	0	0	0	0	0	0	0	0	0	0	0
	second scorer	0	0	0	0	0	0	0	0	0	0	0	0	0	0	0	0
**Total Score**	averaged	3.5	2.5	2.5	2.5	3	2.5	1.5	2.5	2	2	2	3.5	2	2	2	1.5

# Animal : 8

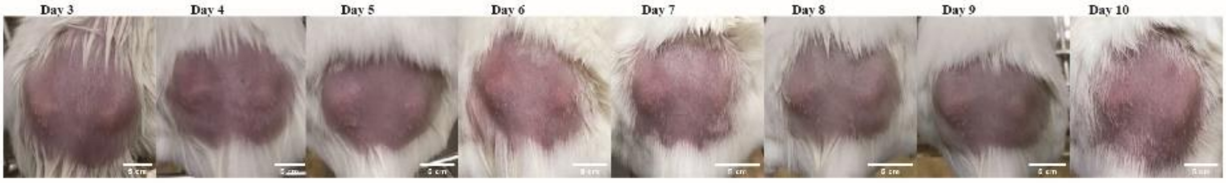

		Day 3		Day 4		Day 5		Day 6		Day 7		Day 8		Day 9		Day 10	
		L	R	L	R	L	R	L	R	L	R	L	R	L	R	L	R
**Diameter Score**	measured	1	2	2	2	2	2	2	2	1	1	1	2	1	1	1	1
**Elevation Score**	first scorer	1	2	2	1	2	1	1	2	1	1	1	1	1	1	1	1
	second scorer	1	2	1	1	1	1	1	1	1	0	1	2	0	1	1	2
**Eschar Score**	first scorer	0	0	0	0	0	0	0	0	0	0	0	0	0	0	0	0
	second scorer	0	0	0	0	0	0	0	0	0	0	0	0	0	0	0	0
**Total Score**	averaged	2	4	3.5	3	3.5	3	3	3.5	2	1.5	2	3.5	1.5	2	2	2.5

# Animal : 9

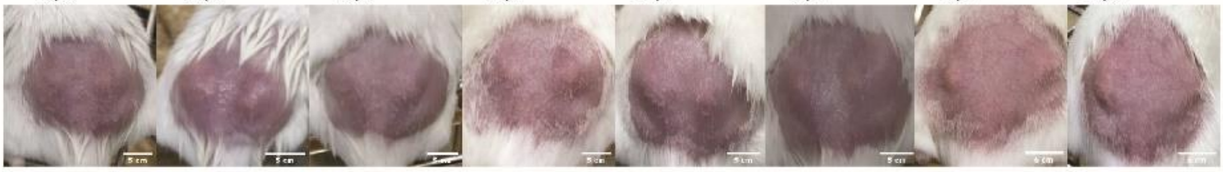

		Day 3		Day 4		Day 5		Day 6		Day 7		Day 8		Day 9		Day 10	
		L	R	L	R	L	R	L	R	L	R	L	R	L	R	L	R
**Diameter Score**	measured	1	2	2	2	1	3	1	2	1	1	1	2	1	2	1	2
**Elevation Score**	first scorer	2	2	0	2	1	2	1	1	1	0	1	1	1	1	1	1
	second scorer	1	1	0	1	1	1	2	2	2	1	0	1	1	1	1	1
**Eschar Score**	first scorer	0	0	0	0	0	0	0	0	0	0	0	0	0	0	0	0
	second scorer	0	0	0	0	0	0	0	0	0	0	0	0	0	0	0	0
**Total Score**	averaged	2.5	3.5	2	3.5	2	4.5	2.5	3.5	2.5	1.5	1.5	3	2	3	2	3

# Animal : 10

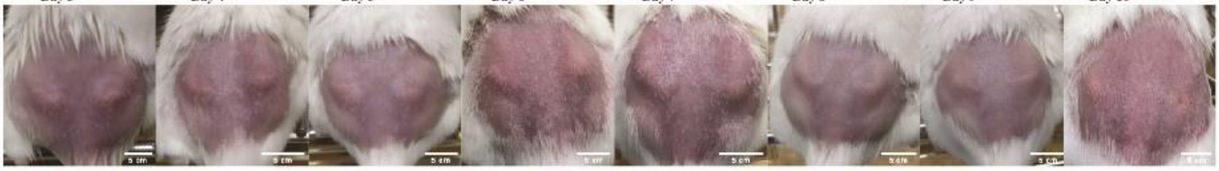

		Day 3		Day 4		Day 5		Day 6		Day 7		Day 8		Day 9		Day 10	
		L	R	L	R	L	R	L	R	L	R	L	R	L	R	L	R
**Diameter Score**	measured	2	3	1	2	2	2	1	2	2	2	2	1	2	1	2	1
**Elevation Score**	first scorer	2	1	2	2	1	1	1	1	1	1	1	1	1	1	1	1
	second scorer	2	1	2	2	1	1	1	1	1	0	1	1	1	1	1	1
**Eschar Score**	first scorer	0	0	0	0	0	0	0	0	0	0	0	0	0	0	0	0
	second scorer	0	0	0	0	0	0	0	0	0	0	0	0	0	0	0	0
**Total Score**	averaged	4	4	3	4	3	3	2	3	3	2.5	32	2	3	2	3	2

Day 3 | Day 4 | Day 5 | Day 6 | Day 7 | Day 8 | Day 9 | Day 10

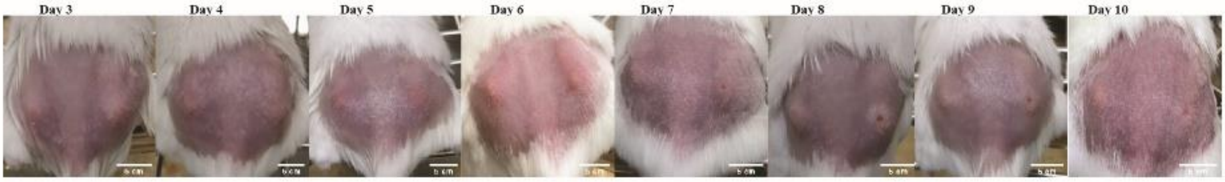

Animal : 11

		Day 3		Day 4		Day 5		Day 6		Day 7		Day 8		Day 9		Day 10	
		L	R	L	R	L	R	L	R	L	R	L	R	L	R	L	R
Diameter Score	measured	2	2	2	2	2	2	1	1	1	0	2	1	1	1	1	0
Elevation Score	first scorer	1	0	1	0	0	1	1	0	0	0	1	1	0	1	1	0
	second scorer	2	1	0	1	1	1	1	1	1	1	1	1	1	1	1	0
Eschar Score	first scorer	0	0	0	0	0	0	0	0	0	0	0	1	0	0	0	0
	second scorer	0	0	0	0	1	0	0	0	0	1	0	1	0	1	0	1
Total Score	averaged	3.5	2.5	2.5	2.5	3	3	2	1.5	1.5	1	3	3	1.5	2.5	2	0.5

Day 3 | Day 4 | Day 5 | Day 6 | Day 7 | Day 8 | Day 9 | Day 10

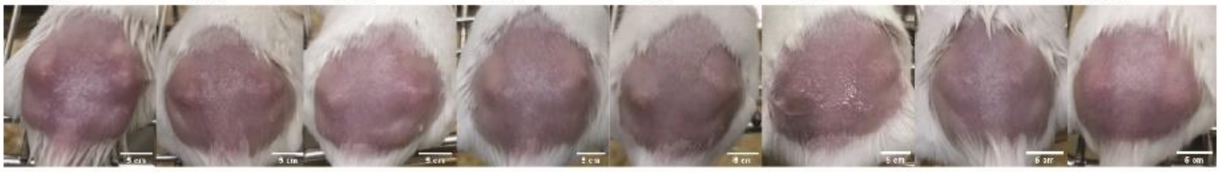

Animal : 12

		Day 3		Day 4		Day 5		Day 6		Day 7		Day 8		Day 9		Day 10	
		L	R	L	R	L	R	L	R	L	R	L	R	L	R	L	R
Diameter Score	measured	3	2	2	2	3	2	2	1	2	1	2	1	1	0	1	1
Elevation Score	first scorer	2	2	2	1	2	1	1	2	2	2	2	1	2	1	1	1
	second scorer	3	3	2	1	2	1	2	1	2	1	2	1	1	0	1	1
Eschar Score	first scorer	0	0	0	0	0	0	0	0	0	0	0	0	0	0	0	0
	second scorer	0	0	0	0	0	0	0	0	0	0	0	0	0	0	0	0
Total Score	averaged	5.5	4.5	4	3	5	3	3.5	2.5	4	2.5	4	2	2.5	0.5	2	2

Day 3 | Day 4 | Day 5 | Day 6 | Day 7 | Day 8 | Day 9 | Day 10

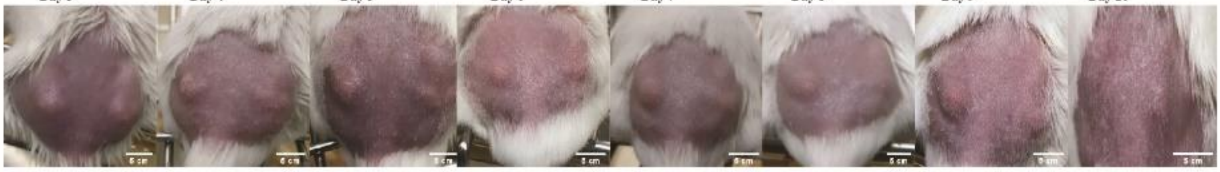

Animal : 13

		Day 3		Day 4		Day 5		Day 6		Day 7		Day 8		Day 9		Day 10	
		L	R	L	R	L	R	L	R	L	R	L	R	L	R	L	R
Diameter Score	measured	2	2	2	2	3	2	2	1	2	1	2	0	2	0	2	0
Elevation Score	first scorer	1	1	1	1	2	1	2	1	2	1	1	1	1	1	1	0
	second scorer	2	2	1	1	2	1	2	1	2	1	1	0	1	0	0	1
Eschar Score	first scorer	0	0	0	0	0	0	0	0	0	0	0	0	0	0	0	0
	second scorer	0	0	0	0	0	0	0	0	0	0	0	0	0	0	0	0
Total Score	averaged	3.5	3.5	3	3	5	3	4	2	4	2	3	0.5	3	0.5	2.5	0.5

Day 3 | Day 4 | Day 5 | Day 6 | Day 7 | Day 8 | Day 9 | Day 10

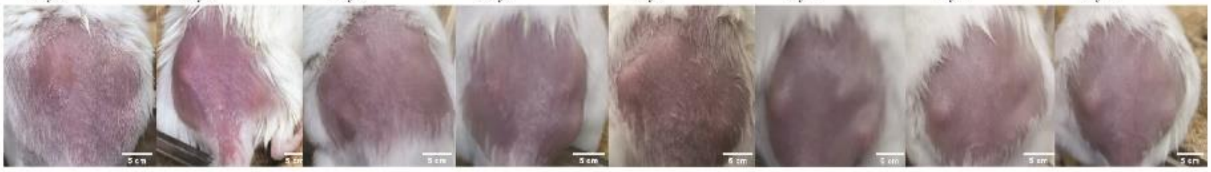

Animal : 14

		Day 3		Day 4		Day 5		Day 6		Day 7		Day 8		Day 9		Day 10	
		L	R	L	R	L	R	L	R	L	R	L	R	L	R	L	R
Diameter Score	measured	1	2	1	2	1	0	1	0	2	1	2	2	2	1	2	1
Elevation Score	first scorer	1	0	1	0	0	0	1	1	2	1	2	1	2	1	2	0
	second scorer	0	0	1	0	0	0	0	0	1	1	1	1	2	1	2	1
Eschar Score	first scorer	0	0	0	0	0	0	0	0	0	0	0	0	0	0	0	0
	second scorer	0	0	0	0	0	0	0	0	0	0	0	0	0	0	0	0
Total Score	averaged	1.5	2	2	2	1	0	1.5	0.5	3.5	2	3.5	3	4	2	4	1.5

Day 3 | Day 4 | Day 5 | Day 6 | Day 7 | Day 8 | Day 9 | Day 10

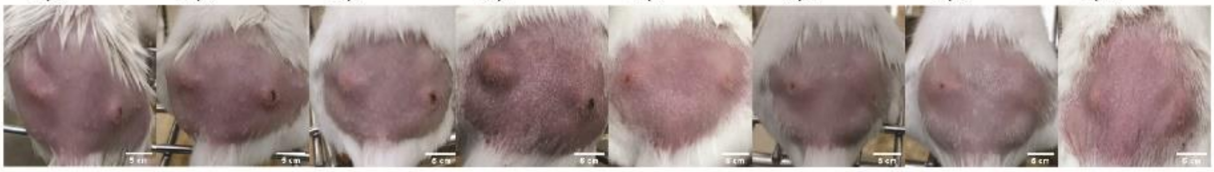

Animal : 15

		Day 3		Day 4		Day 5		Day 6		Day 7		Day 8		Day 9		Day 10	
		L	R	L	R	L	R	L	R	L	R	L	R	L	R	L	R
Diameter Score	measured	2	2	2	2	3	1	2	1	2	1	2	2	2	1	2	1
Elevation Score	first scorer	2	1	2	2	2	1	2	1	1	1	1	1	1	0	1	0
	second scorer	2	1	2	2	2	1	2	1	1	1	1	1	1	1	1	1
Eschar Score	first scorer	0	1	0	2	0	2	0	2	1	1	1	0	1	0	0	0
	second scorer	0	2	0	2	0	2	0	2	2	0	2	0	1	0	0	0
Total Score	averaged	4	4.5	4	6	5	4	4	4	4.5	2.5	4.5	3	4	1.5	3	1.5

Animal : 16

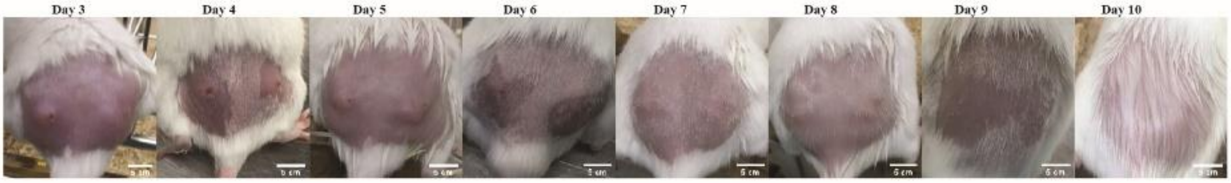

		L	R	L	R	L	R	L	R	L	R	L	R	L	R	L	R
**Diameter Score**	measured	3	2	2	1	2	1	0	0	1	1	1	0	0	0	0	0
**Elevation Score**	first scorer	1	1	1	1	1	1	0	0	0	0	0	0	0	0	0	0
	second scorer	1	1	0	1	1	1	0	0	0	0	0	0	0	0	0	0
**Eschar Score**	first scorer	2	0	2	1	1	1	1	0	0	0	0	0	0	0	0	0
	second scorer	2	0	2	2	1	0	1	0	0	0	0	0	0	0	0	0
**Total Score**	averaged	**6**	**3**	**4.5**	**3.5**	**4**	**2.5**	**1**	**0**	**1**	**1**	**1**	**0**	**0**	**0**	**0**	**0**

Animal : 17

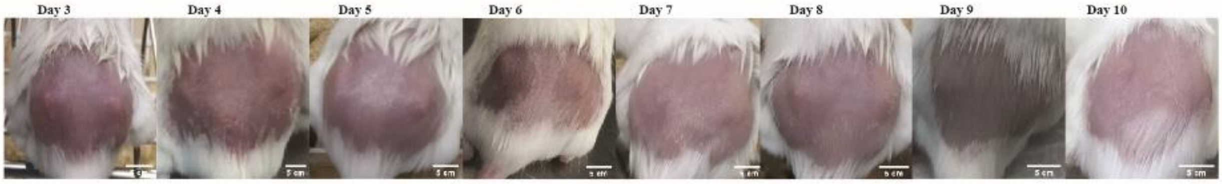

		L	R	L	R	L	R	L	R	L	R	L	R	L	R	L	R
**Diameter Score**	measured	2	2	2	2	2	2	2	1	2	1	2	1	1	1	1	1
**Elevation Score**	first scorer	2	2	2	1	1	1	1	1	1	1	2	1	1	1	1	0
	second scorer	2	2	1	1	1	1	1	1	1	1	1	1	1	1	1	0
**Eschar Score**	first scorer	0	0	0	0	0	0	0	0	0	0	0	0	0	0	0	0
	second scorer	0	0	0	0	0	0	0	0	0	0	0	0	0	0	0	0
**Total Score**	averaged	**4**	**4**	**3.5**	**3**	**3**	**3**	**3**	**2**	**3**	**2**	**3.5**	**2**	**2**	**2**	**2**	**1**

Table S5.

List of antibodies used in this study. Their name, dilution used in this manuscript, validation reference, company name, catalog, and clone number are described.

Table S5.

Name	Dilution	Validation	Company name	Catalog number	Clone number
CD115	1:100	Hong *et al.* [4]	BioLegend (San Diego, CA)	135509	AFS98
CD45	1:100	Davies *et al.* [5]	BioLegend (San Diego, CA)	103101	30-f11
CD64	1:100	Fujiyama *et al.* [6]	BioLegend (San Diego, CA)	139303	X54-5/7.1
CD11b	1:100	Davies *et al.* [5]	BioLegend (San Diego, CA)	101201	M1/70
LY6C	1:100	Hoving *et al.* [7]	BioLegend (San Diego, CA)	128015	HK1.4
LY6G	1:100	Davies *et al.* [5]	BioLegend (San Diego, CA)	127607	IA8
Zombie UV™	1:100	Fujiyama *et al.* [6]	BioLegend (San Diego, CA)	423107	n/a
LY6G	1:100	Koren *et al.* [8]	BD Biosciences, Franklin Lakes, NJ	394206	IA8
Goat anti-rat igG	1:200	Koren *et al.* [8]	Invitrogen, Waltham, MA	11006	n/a

Table S6.

List of primers used in this study. The name of the gene, the Forward and reverse sequence, reference of their source are described.

Table S6.

Gene	Forward	Reverse	Source
18S	CGG CTA CCA CAT CCA AGG AA	GGG CCT CGA AAG AGT CCT GTA T	Koren *et al* .[8]
CXCL2	TGA ACA AAG GCA AGG CTA ACT	CAG GTA CGA TCC AGG CTT CC	Koren *et al.* [8]
TNF-alpha	CCC AGG GAC CTC TCT CTA ATC A	AGC TGC CCC TCA GCT TGA G	Nassar *et al.* [9]
IL1-beta	CAA CCA ACA AGT GAT ATT	GAT CCA CAC TCT CCA GCT	Mizraji *et al.* [10]
IL-6	AGT TGC CTT CTT GGG ACT G	CAG AAT TGC CAT TGC ACA A	Nassar *et al.* [9]
IL-17	CAC TTT GCC TCC CAG ATC AC	ACC AAT CCC AAA AGG TCC TC	Nassar *et al.* [9]

Supplementary References

1. Rohrschneider, M. *et al.* Evaluation of the transwell system for characterization of dissolution behavior of inhalation drugs: effects of membrane and surfactant. *Mol. Pharm.* **12**, 2618–2624 (2015).
2. Boivin, G. *et al.* Durable and controlled depletion of neutrophils in mice. *Nat. Commun.* **11**, 2762 (2020).
3. Niderla-Bielińska, J. *et al.* A comprehensive miRNome analysis of macrophages isolated from db/db mice and selected miRNAs involved in metabolic syndrome-associated cardiac remodeling. *Int. J. Mol. Sci.* **22**, 2197 (2021).
4. Hong, H. *et al.* Interleukin-3 plays dual roles in osteoclastogenesis by promoting the development of osteoclast progenitors but inhibiting the osteoclastogenic process. *Biochem. Biophys. Res. Commun.* **440**, 545–550 (2013).
5. Davies, M. L. *et al.* A systemic macrophage response is required to contain a peripheral poxvirus infection. *PLoS Pathog.* **13**, e1006435 (2017).
6. Fujiyama, S. *et al.* Identification and isolation of splenic tissue-resident macrophage sub-populations by flow cytometry. *Int. Immunol.* **31**, 51–56 (2019).
7. Hoving, L. R. *et al.* Dietary yeast-derived mannan oligosaccharides have immune-modulatory properties but do not improve high fat diet-induced obesity and glucose intolerance. *PLoS One* **13**, e0196165 (2018).
8. Koren, N. *et al.* Maturation of the neonatal oral mucosa involves unique epithelium-microbiota interactions. *Cell Host Microbe* **29**, 197–209 (2021).
9. Nassar, M. *et al.* GAS6 is a key homeostatic immunological regulator of host–commensal interactions in the oral mucosa. *Proc. Natl. Acad. Sci.* **114**, E337–E346 (2017).
10. Mizraji, G., Heyman, O., Van Dyke, T. E. & Wilensky, A. Resolvin D2 restrains Th1 immunity and prevents alveolar bone loss in murine periodontitis. *Front. Immunol.* **9**, 785 (2018).

<u>Publication licenses for figures:</u>

Figure 3a

Supplemental Figure S5

49 Spadina Ave. Suite 200
Toronto ON M5V 2J1 Canada
www.biorender.com

Confirmation of Publication and Licensing Rights

February 2nd, 2023
Science Suite Inc.

Subscription: *Student Plan*
Agreement number: *YA24YQ3L1K*
Journal name: *Nature Communications*

To whom this may concern,

This document is to confirm that Amit Rimon has been granted a license to use the BioRender content, including icons, templates and other original artwork, appearing in the attached completed graphic pursuant to BioRender's Academic License Terms. This license permits BioRender content to be sublicensed for use in journal publications.

All rights and ownership of BioRender content are reserved by BioRender. All completed graphics must be accompanied by the following citation: "Created with BioRender.com".

BioRender content included in the completed graphic is not licensed for any commercial uses beyond publication in a journal. For any commercial use of this figure, users may, if allowed, recreate it in BioRender under an Industry BioRender Plan.

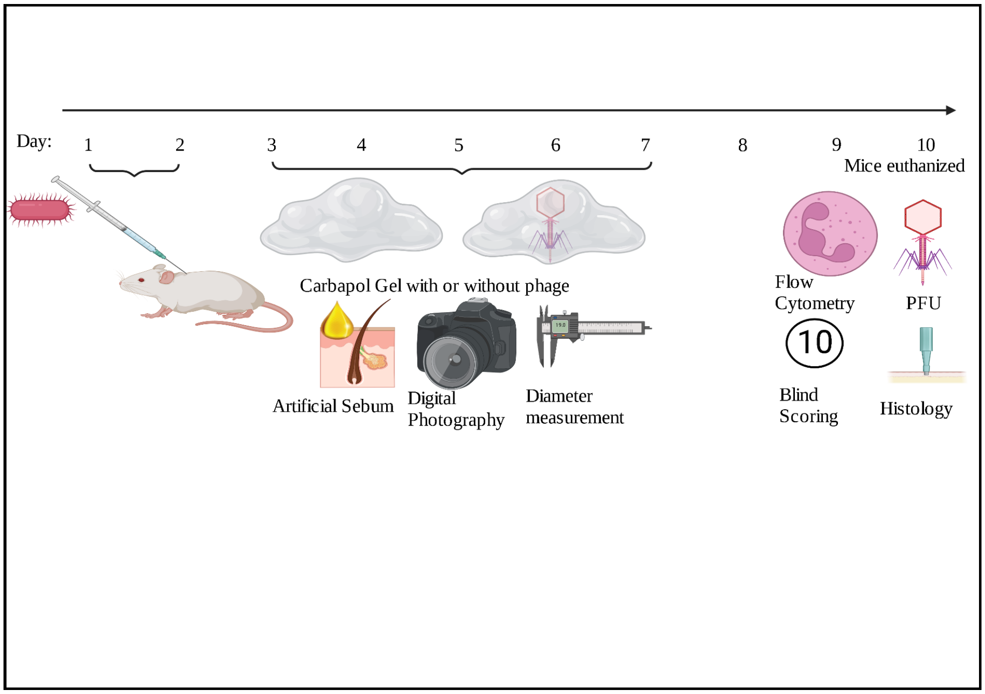

For any questions regarding this document, or other questions about publishing with BioRender refer to our BioRender Publication Guide, or contact BioRender Support at support@biorender.com.

49 Spadina Ave. Suite 200
Toronto ON M5V 2J1 Canada
www.biorender.com

Confirmation of Publication and Licensing Rights

February 2nd, 2023
Science Suite Inc.

Subscription: *Student Plan*
Agreement number: *AM24YQ57FP*
Journal name: *Nature Communications*

To whom this may concern,

This document is to confirm that Amit Rimon has been granted a license to use the BioRender content, including icons, templates and other original artwork, appearing in the attached completed graphic pursuant to BioRender's Academic License Terms. This license permits BioRender content to be sublicensed for use in journal publications.

All rights and ownership of BioRender content are reserved by BioRender. All completed graphics must be accompanied by the following citation: "Created with BioRender.com".

BioRender content included in the completed graphic is not licensed for any commercial uses beyond publication in a journal. For any commercial use of this figure, users may, if allowed, recreate it in BioRender under an Industry BioRender Plan.

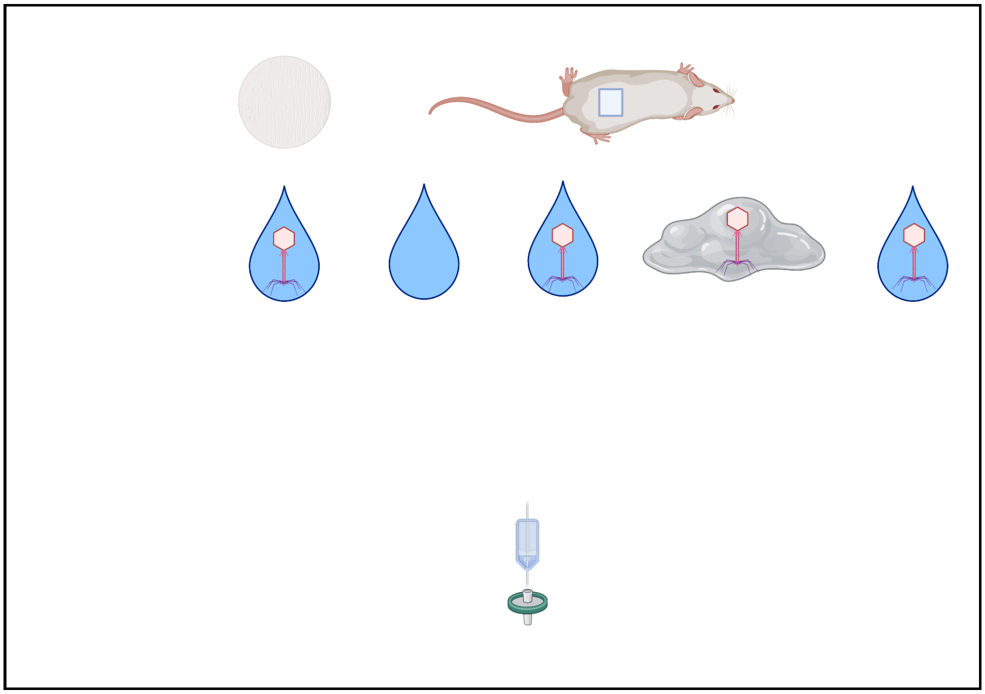

For any questions regarding this document, or other questions about publishing with BioRender refer to our BioRender Publication Guide, or contact BioRender Support at support@biorender.com.
