# Supplementary material for: Topical phage therapy in a mouse model of Cutibacterium acnes-induced acne-like lesions
Source: Nat Commun. 2023 Feb 22;14:1005. doi: 10.1038/s41467-023-36694-8 (PMC9947178; doi:10.1038/s41467-023-36694-8)
Supplement: Supplementary file 3 — Reporting Summary [file 41467_2023_36694_MOESM3_ESM.pdf]

nature portfolio

Corresponding author(s): Prof. Ronen Hazan

Last updated by author(s): Jan 8, 2023

Reporting Summary

Nature Portfolio wishes to improve the reproducibility of the work that we publish. This form provides structure for consistency and transparency in reporting. For further information on Nature Portfolio policies, see our Editorial Policies and the Editorial Policy Checklist.

Statistics

For all statistical analyses, confirm that the following items are present in the figure legend, table legend, main text, or Methods section.

n/a	Confirmed	
☐	☒	The exact sample size (*n*) for each experimental group/condition, given as a discrete number and unit of measurement
☐	☒	A statement on whether measurements were taken from distinct samples or whether the same sample was measured repeatedly
☐	☒	The statistical test(s) used AND whether they are one- or two-sided<br>*Only common tests should be described solely by name; describe more complex techniques in the Methods section.*
☐	☒	A description of all covariates tested
☐	☒	A description of any assumptions or corrections, such as tests of normality and adjustment for multiple comparisons
☐	☒	A full description of the statistical parameters including central tendency (e.g. means) or other basic estimates (e.g. regression coefficient) AND variation (e.g. standard deviation) or associated estimates of uncertainty (e.g. confidence intervals)
☐	☒	For null hypothesis testing, the test statistic (e.g. *F*, *t*, *r*) with confidence intervals, effect sizes, degrees of freedom and *P* value noted<br>*Give P values as exact values whenever suitable.*
☒	☐	For Bayesian analysis, information on the choice of priors and Markov chain Monte Carlo settings
☐	☒	For hierarchical and complex designs, identification of the appropriate level for tests and full reporting of outcomes
☒	☐	Estimates of effect sizes (e.g. Cohen's *d*, Pearson's *r*), indicating how they were calculated

Our web collection on statistics for biologists contains articles on many of the points above.

Software and code

Policy information about availability of computer code

Data collection	NA
Data analysis	Genious Prime GraphPad Prism 8.0.2 (GraphPad Software, Inc., La Jolla, CA), Genious Prime (Version 2022.2.1 and 2022.2.2 , New Zeland) , using ImageJ software (V.1.53), Illustrator (Version 24.1, Adobe, San Jose, CA). In some figures illustration were made using Biorender (biorender.com), publication license was granted (Supplementary file).

For manuscripts utilizing custom algorithms or software that are central to the research but not yet described in published literature, software must be made available to editors and reviewers. We strongly encourage code deposition in a community repository (e.g. GitHub). See the Nature Portfolio guidelines for submitting code & software for further information.

Data

Policy information about availability of data

All manuscripts must include a data availability statement. This statement should provide the following information, where applicable:

- Accession codes, unique identifiers, or web links for publicly available datasets
- A description of any restrictions on data availability
- For clinical datasets or third party data, please ensure that the statement adheres to our policy

Data Availability All data presented herein are available in the main text, supplementary materials, source file, or data depositories. The phage sequences generated

in this study were deposited in the GenBank (https://www.ncbi.nlm.nih.gov) with the accession codes described in Table 2. Images not displayed in the Supplementary can be found in the BioStudies data repository (https://www.ebi.ac.uk/biostudies/studies/S-BSST889). Source data are provided with this paper.

Human research participants

Policy information about studies involving human research participants and Sex and Gender in Research.

Reporting on sex and gender	As described in table 1, our human sample collection contains both men and woman, 75% woman, 25% men.
Population characteristics	As described in table 1, our median age is 19, age range is from 12 to 30. mild moderate and severe acne vulgaris.
Recruitment	Acne vulgaris patients over 10 years of age attending the Dermatology Clinic at Hadassah Hebrew University Medical Center, were invited to participate in the study. Following a detailed explanation of the study, written informed consent was obtained from the participants and/or their parents. Clinical information, including age, sex, age of onset, previous or current anti-acne treatments, acne severity (according to the Physician Global Assessment) and acne distribution, were obtained from medical records.<br>A possible bias is that participants were recruited from a tertiary medical center and may therefore over represent severe acne or failure of treatment due to bacterial resistance.
Ethics oversight	Clinical and demographic information collection for this study was approved by the local ethics review board, Hadassah Medical Center Helsinki Committee (HMO-0073-19). Clinically isolated bacteria and phage sample collection for this study were approved by the local ethics review board, Hadassah Medical Center Helsinki Committee (HMO-0212-17) Informed consent was given by patients and their parents in case of a minor participant.

Note that full information on the approval of the study protocol must also be provided in the manuscript.

Field-specific reporting

Please select the one below that is the best fit for your research. If you are not sure, read the appropriate sections before making your selection.

☒ Life sciences ☐ Behavioural & social sciences ☐ Ecological, evolutionary & environmental sciences

For a reference copy of the document with all sections, see nature.com/documents/nr-reporting-summary-flat.pdf

Life sciences study design

All studies must disclose on these points even when the disclosure is negative.

Sample size	In the in vitro studies such as growth kinetics, bacterial and phage quantification, triplicates were made as it is the standard n in these experiments.<br>for the first animal experiment that assessed clinical efficacy comparing 3 groups, a total of 38 mice. The sample size was calculated with the size calculator ClinCalc (https://clincalc.com/stats/samplesize.aspx), with a preliminary estimation of a score of 1.9 ± 0.4 AU in the infected-untreated group and 1.5 AU in the phage-treated group (n =16). The sham injection control mice number was determined according to the minimum number of mice needed to complete all experiments, as no effect was expected, and we aimed to follow the minimization requirement, to use as few mice as possible. for the second animal experiment assessing inflammation kinetics a preliminary experiment in which day 7 sacrifice was made, which was the goal of this experiment. Preliminary estimation was 45 ± 14 %Noutophil in the infected-untreated group and 21 %Noutophil in the treated group (n =5, ClinCalc sample size calculator), in order to gain other time points, a minimal n=3 was used as described in Table 4 in the manuscript (a total of 34 mice).
Data exclusions	No data was excluded from this experiment.
Replication	All attempts at replication were successful. All of the in-vitro experiments were repeated twice, including electron microscopy, growth kinetics, bacterial phage sensitivity, and phage stability. ex-vivo experiments in which the biological sample was stable have undergone repetition such as PCR, Elisa. in vivo experiments that were representative experiments such as histology, immunohistochemistry, and skin crossing ability were repeated as well. full-scale in-vivo studies were first assessed on a smaller scale to assess the effect and then repeated according to the sample size described above.
Randomization	Mice were randomized to either control or treatment groups simply by following serial number allocation, in in-vitro experiments, randomization was not needed as all of grown bacteria were grown in similar conditions, and often on the same plate. bacteria was not put in the extremities of the 96 cell plate as this rows might evaporate and alter experiments conditions.
Blinding	Mice scoring was blinded in the infected-untreated and treated group, so that the scorer did not know which mice he/she was scoring, or what treatment did it receive. each mouse was scored by two independent scorers as described in the text. the histological score was given by a professional mice pathologist, who was blinded as well. As other experiments done such as electron microscopy, growth kinetics, phage and bacterial quantifications, immunohistochemistry, flow cytometry, RT PCR and Elisa, were objective they were not blinded.

Reporting for specific materials, systems and methods

We require information from authors about some types of materials, experimental systems and methods used in many studies. Here, indicate whether each material, system or method listed is relevant to your study. If you are not sure if a list item applies to your research, read the appropriate section before selecting a response.

Materials & experimental systems

n/a	Involved in the study	
☐	☒	Antibodies
☒	☐	Eukaryotic cell lines
☒	☐	Palaeontology and archaeology
☐	☒	Animals and other organisms
☒	☐	Clinical data
☒	☐	Dual use research of concern

Methods

n/a	Involved in the study	
☒	☐	ChIP-seq
☐	☒	Flow cytometry
☒	☐	MRI-based neuroimaging

Antibodies

Antibodies used	For Flow Cytometry: antibodies obtained from BioLegend (San Diego, CA, USA) at a 1:100 dilution: CD115 (Clone #: AFS98, Catalog #: 135509), CD45 (Clone #: 30-f11, Catalog #: 103101), CD64 (Clone #: X54-5/7.1, Catalog #: 139303), CD11b (Clone #: M1/70, Catalog #: 101201), LY6C (Clone #: HK1.4, Catalog #: 128015), LY6G (Clone #: 1A8, Catalog #: 127607), and Zombie UV™ (Catalog #: 423107) for dead cell exclusion<br>For Immunofluorescence:<br>1:100 primary rat anti-mouse Ly6G (Clone #: IA8, Catalog #: 394206, BD biosciences, Franklin Lakes, NJ), 1:200 Second antibiody Goat anti-rat igG (Catalog #: 11006, Invitrogen, Waltham, MA)
Validation	Antibodies used in Flow Cytometry:<br>CD115 : Hong H, et al. 2013. Biochem Biophys Res Commun. 440:545.<br>CD45, CD11b, LY6G : Davies M, et al. 2017. PLoS Pathogens. 13(6):e1006435<br>LY6C : Hoving LR, et al. 2018. PloS One. 13(5): e0196165.<br>CD64, Zombie UV: Fujiyama S, et al. 2019. International immunology. 31(1), 51-56.<br><br>For Immunofluorescence:<br>Ly6G (BD biosciences), Goat anti-rat igG: Koren N, et al. 2021. Cell Host & Microbe. 29(2), 197-209.

Animals and other research organisms

Policy information about studies involving animals; ARRIVE guidelines recommended for reporting animal research, and Sex and Gender in Research

Laboratory animals	Eight week old ICR mice. Housing conditions were 21-24°C , 30-70% humidity, with light in the room for 12 hours, between 7:00-19:00, and dark for 12 hours from 19:00-7:00.
Wild animals	No wild animals were used in this study.
Reporting on sex	Female mice were used, as only demale mice were used d before when using the described model (Stacey et al.)
Field-collected samples	No field collected samples were used in this study.
Ethics oversight	The in-vivois section of this study was approved by the Authority for Biological and Biomedical Models at Hadasssah Medical Center in Jerusalem, Israel (Approval number: MD1815519\3, MD2217957\4)

Note that full information on the approval of the study protocol must also be provided in the manuscript.

Flow Cytometry

Plots

Confirm that:

☒ The axis labels state the marker and fluorochrome used (e.g. CD4-FITC).

☒ The axis scales are clearly visible. Include numbers along axes only for bottom left plot of group (a 'group' is an analysis of identical markers).

☒ All plots are contour plots with outliers or pseudocolor plots.

☒ A numerical value for number of cells or percentage (with statistics) is provided.

Methodology

Sample preparation	Extraction of immune cells from skin biopsies was performed using the protocol of Lou et al. 55, with some modifications to the Dispase II concentration and incubation time. Briefly, 1 cm × 1 cm of skin was taken from the inflammatory lesions of sacrificed mice. The skin was washed in Hanks' Balanced Salt Solution (HBSS) three times, cut into four diagonally and incubated with 8 mg/ml Dispase II (Merck, Kenilworth, New Jersey) for 12 h. Following dermis and epidermis separation, the dermis was cut and put in 3.5 ml of Dermis Dissociation Buffer composed of 100 µg/mL of DNASE I (Merck) and 1 mg/ml collagenase P (Merck, Kenilworth New Jersey) in Dulbecco's Modified Eagle Medium (DMEM/high glucose) (Merck, Kenilworth New Jersey, USA) for 1 h. Suspensions were passed through a 40-µm strainer into a 50 ml tube and rinsed again in 12 ml of DMEM with 10% Fetal bovine serum (FBS) (Merck, Kenilworth, New Jersey) in 15 ml tubes, centrifuged at 400 g for 5 min at 4?, supplied with 2 ml of staining buffer 55 composed of PBS with 2% Fetal calf serum (FBS (Merck, Kenilworth, New Jersey, USA), and fixated with 250 µl BD Cytofix™ (BD Biosciences, Franklin Lakes, New Jersey, USA).<br>Single-cell suspensions were incubated and labeled with the following antibodies obtained from BioLegend (San Diego, CA, USA) at a 1:100 dilution: CD115 (AFS98), CD45 (30-f11), CD64 (X54-5/7.1), CD11b (M1/70), LY6C (HK1.4), LY6G (1A8), and Zombie UV™ for dead cell exclusion. Following membrane staining, the cells were fixed using a Fixation/Permeabilization Solution Kit (BD) according to the manufacturer's instructions.
Instrument	Flow cytometry was performed using Cytek® Aurora (Cytek, Fremont, CA, USA).
Software	Data were analyzed offline using FlowJo 10.7.2 (BD Biosciences, Franklin Lakes, New Jersey, USA).
Cell population abundance	SSC-H, FSC-H population abundance averaged 17.4% with a Standard deviation (SD) of 9.4% in the Sham injected group, 18.7% SD 5.3% in the Phage control group, 50.9% SD 20.0% in the Infected untreated group, and 58.0% SD 16.9% in the Phage treated group.<br>Single cells averaged 72.54% with a SD of 6.8% in the Sham injected group, 71.7% SD 3.8% in the Phage control group, 64.8% SD 6.4% in the Infected untreated group, and 54.3% SD 14.2% in the Phage treated group.<br>Live cells averaged 94.1% with a SD of 5.7% in the Sham injected group, 91.3% SD 10.0% in the Phage control group, 84.6% SD 4.7% in the Infected untreated group, and 91.3% SD 3.95% in the Phage treated group.<br>CD11b+, CD45+ cells averaged 0.7% with a SD of 0.3% in the Sham injected group, 1.5% SD 3.4% in the Phage control group, 39% SD 20.1% in the Infected untreated group, and 26.6% SD 13.2% in the Phage treated group.<br>Ly6G+, CD64- cells averaged 4.8% with a SD of 1.9% in the Sham injected group, 6.8% SD 5.5% in the Phage control group, 48.4% SD 20.0% in the Infected untreated group, and 23.6% SD 14.3% in the Phage treated group.<br>CD64+, Ly6G- cells averaged 7.3% with a SD of 4.0% in the Sham injected group, 12.6% SD 8.4% in the Phage control group, 22.1% SD 11.2% in the Infected untreated group, and 36.3% SD 15.6% in the Phage treated group.
Gating strategy	Gating strategy for both cell population is described in Supplemental Figure S8B. Cells were first gated on forward scatter (FSC-A) and side scatter (SSC-A) to exclude debris, based on the relative size and complexity of the cells. Events with lower forward and side scatter levels are found at the bottom left corner of the dot plot, representing the debris, while our cells of interest would have higher values. Then single cells were gated using FSC-H and FSC-A. The distribution between the height and the area can be used to identify doublets because they will have double the area values of single cells, while the height is approximately the same. Viable cells were gated as Zombie negative. Myeloid leukocytes were gated as CD45+ and CD11b+. Neutrophils were identified as Ly6G positive, CD64 negative while Macrophages were CD64 positive, Ly6G negative

☒ Tick this box to confirm that a figure exemplifying the gating strategy is provided in the Supplementary Information.
